# Whole exome sequencing implicates eye development, the unfolded protein response and plasma membrane homeostasis in primary open-angle glaucoma

**DOI:** 10.1371/journal.pone.0172427

**Published:** 2017-03-06

**Authors:** Tiger Zhou, Emmanuelle Souzeau, Shiwani Sharma, John Landers, Richard Mills, Ivan Goldberg, Paul R. Healey, Stuart Graham, Alex W. Hewitt, David A. Mackey, Anna Galanopoulos, Robert J. Casson, Jonathan B. Ruddle, Jonathan Ellis, Paul Leo, Matthew A. Brown, Stuart MacGregor, David J. Lynn, Kathryn P. Burdon, Jamie E. Craig

**Affiliations:** 1 Flinders University, Department of Ophthalmology, Bedford Park, South Australia, Australia; 2 University of Sydney Discipline of Ophthalmology, Sydney, Australia; 3 Glaucoma Unit, Sydney Eye Hospital, Sydney, Australia; 4 Centre for Vision Research, Westmead Institute for Medical Research, University of Sydney, Sydney, Australia; 5 University of Tasmania Menzies Institute for Medical Research, Hobart, Australia; 6 University of Western Australia Centre for Ophthalmology and Visual Science, Lions Eye Institute, Perth, Australia; 7 University of Adelaide, Discipline of Ophthalmology & Visual Sciences, Adelaide, Australia; 8 Centre for Eye Research Australia, Royal Victorian Eye and Ear Hospital, Melbourne, Australia; 9 University of Queensland Diamantina Institute, Translational Research Institute, Princess Alexandra Hospital, Woolloongabba, Australia; 10 Statistical Genetics, QIMR Berghofer Medical Research Institute, Royal Brisbane Hospital, Brisbane, Australia; 11 EMBL Australia Group, Infection & Immunity Theme, South Australian Medical and Health Research Institute, Adelaide, Australia; 12 Flinders University, School of Medicine, Adelaide, Australia; National Eye Institute, UNITED STATES

## Abstract

**Purpose:**

To identify biological processes associated with POAG and its subtypes, high-tension (HTG) and normal-tension glaucoma (NTG), by analyzing rare potentially damaging genetic variants.

**Methods:**

A total of 122 and 65 unrelated HTG and NTG participants, respectively, with early onset advanced POAG, 103 non-glaucoma controls and 993 unscreened ethnicity-matched controls were included in this study. Study participants without myocilin disease-causing variants and non-glaucoma controls were subjected to whole exome sequencing on an Illumina HiSeq2000. Exomes of participants were sequenced on an Illumina HiSeq2000. Qualifying variants were rare in the general population (MAF < 0.001) and potentially functionally damaging (nonsense, frameshift, splice or predicted pathogenic using SIFT or Polyphen2 software). Genes showing enrichment of qualifying variants in cases were selected for pathway and network analysis using InnateDB.

**Results:**

POAG cases showed enrichment of rare variants in camera-type eye development genes (p = 1.40×10–7, corrected p = 3.28×10–4). Implicated eye development genes were related to neuronal or retinal development. HTG cases were significantly enriched for key regulators in the unfolded protein response (UPR) (p = 7.72×10–5, corrected p = 0.013). The UPR is known to be involved in myocilin-related glaucoma; our results suggest the UPR has a role in non-myocilin causes of HTG. NTG cases showed enrichment in ion channel transport processes (p = 1.05×10–4, corrected p = 0.027) including calcium, chloride and phospholipid transporters involved in plasma membrane homeostasis. Network analysis also revealed enrichment of the MHC Class I antigen presentation pathway in HTG, and the EGFR1 and cell-cycle pathways in both HTG and NTG.

**Conclusion:**

This study suggests that mutations in eye development genes are enriched in POAG. HTG can result from aberrant responses to protein misfolding which may be amenable to molecular chaperone therapy. NTG is associated with impaired plasma membrane homeostasis increasing susceptibility to apoptosis.

## Introduction

Primary open-angle glaucoma (POAG) is a leading cause of irreversible blindness worldwide.[1] Epidemiological evidence has demonstrated a strong genetic component to POAG with a heritability of 0.81[2] and a 9.2-fold familial increase in disease risk among first-degree relatives of an affected individual.[3] Historically, POAG was thought to be solely a disease of raised intraocular pressure (IOP); however, this concept does not capture the full spectrum of the disease.[4, 5] Wide phenotypic heterogeneity exists within the disorder—despite the majority of patients exhibiting elevated IOP (high-tension glaucoma (HTG)), many others develop vision loss with no recorded elevation in their IOP (normal-tension glaucoma (NTG)). This is a reflection of the complex gene-environment interactions which drive the pathophysiology. Efforts to decipher the genetic complexity of POAG began in the 1990s with linkage studies on large affected families with phenotypic homogeneity. Certain high penetrance mutations with Mendelian inheritance in *myocilin* (*MYOC*), *optineurin* (*OPTN*) and c*ytochrome P450 family 1 subfamily B polypeptide 1* (*CYP1B1*), and copy number variations of *TANK-binding kinase 1* (*TBK1*) were discovered.[6] Mutations in *MYOC* and *CYP1B1* are causative for HTG, [7, 8] while mutations in *OPTN* and copy-number variations of *TBK1* cause NTG.[4, 5, 9] Despite these successes, highly penetrant Mendelian mutations in genes discovered to date only account for around 5% of all cases of POAG.[6]

The quest to explain the remaining missing heritability has continued during the era of genome-wide association studies (GWAS) using DNA microarray technology. Assuming a common disease, common variant model, this approach has been successful with several disease associated genes being discovered.[10, 11] However, the greatest risk effect of any disease associated single nucleotide polymorphism (SNP) is substantially less than 2 fold in magnitude.[10, 12] Many people without POAG also carry disease-associated alleles while never developing the condition, indicating that these SNPs are associated risk factors for POAG, but are not sufficient to cause disease. SNP microarrays are less suited to the evaluation of rare variants, which may account for a significant portion of the missing heritability.[13] Next-generation sequencing offers new ways to identify rare disease-associated variants with fewer restrictions than traditional linkage studies, which generally require large pedigrees. Some rare variants are likely to have larger effect sizes than common variants[14] and thus are more likely to initiate disease. In terms of clinical application, rare variants may have much greater positive predictive values than associated SNPs from GWAS. The drawback to rare variant analysis is the need for large sample sizes potentially in the magnitude of thousands to achieve statistical significance at a genome-wide level for the discovery a single causative gene.[15] Using even the economical next-generation sequencing technique of whole exome sequencing (WES), the current costs and bioinformatics challenges of this venture are not trivial.

Systems-medicine approaches, which employ network and pathway analysis methods, are an emerging tool to identify signatures of rare variant-disease associations that would not be identifiable in gene-by-gene based analyses. Recent successes have been achieved combining whole exome sequencing and pathway analysis in schizophrenia[16] and amyotrophic lateral sclerosis.[17] In the field of glaucoma research, no such WES studies have been published. The current study investigates the underlying biological mechanisms in POAG pathogenesis and its subtypes utilizing pathway and network analysis of rare variant signals from whole exome sequencing.

## Methods

This prospective case control study was performed under the principles of the revised Declaration of Helsinki and the Australian National Health and Medical Research Council (NHMRC) statement of ethical conduct in research involving humans. Ethical approval was obtained from the Southern Adelaide and Flinders University Clinical Research Ethics Committee. Written informed consent was obtained from all study participants for the use and storage of DNA for research purposes.

### Participants

**Fig 1:**
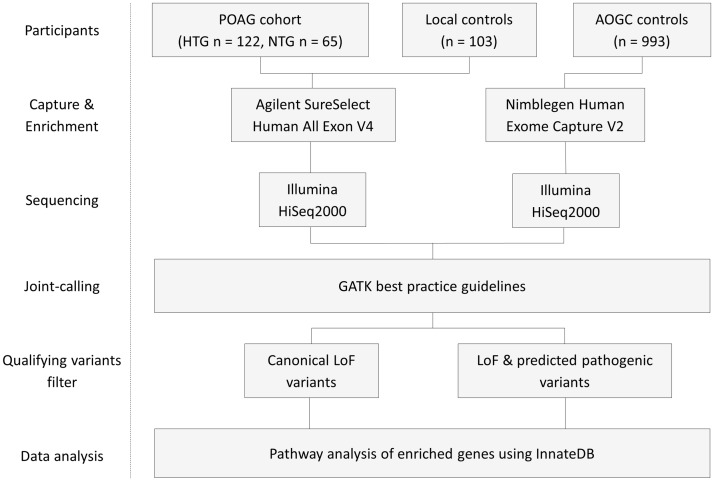
Experimental flowchart. POAG = primary open-angle glaucoma, HTG = high-tension glaucoma, NTG = normal-tension glaucoma, LoF = loss of function.

All peripheral blood samples for this study were collected as a part of the Australian and New Zealand Registry of Advanced Glaucoma (ANZRAG).[18] DNA was extracted from peripheral blood samples using the QIAamp^®^ DNA blood kit (Qiagen, Hilden, Germany) following the manufacturer’s protocol. Inclusion criteria for ANZRAG have been previously described.[18] Briefly, participants included in the study had severe glaucoma defined by glaucomatous visual field loss involving at least 2 of the 4 central fixation squares and a pattern standard deviation of less than 0.5% on a reliable Humphrey 24–2 field (Carl Zeiss, Dublin, CA), or a mean deviation of at most -22 dB in the worst affected eye. Participants who had no recorded visual field score were only included if their best-corrected visual acuity was worse than 20/200 with clinical signs consistent with severe glaucomatous damage. All participants also had demonstrated glaucomatous visual field loss in the less affected eye, with corresponding neuro-retinal rim thinning. After satisfying the visual field criteria, participants with the youngest age of diagnosis (mean = 44.4 years, SD = 10.4 years) were selected for whole exome sequencing and inclusion in this study. Study participants were divided by IOP into HTG and NTG for analysis (Fig 1). NTG was defined as having a maximum recorded untreated IOP of less than 22 mmHg with the remainder of participants designated as HTG. Participants in ANZRAG with known disease-causing mutations in *MYOC*, identified by direct sequencing prior to the current study[18], were excluded from whole exome sequencing. Local controls were examined to ensure absence of clinically evident glaucoma or glaucoma associated phenotype including cupping of the optic disc and elevated IOP. A larger unscreened control cohort from the Australian Osteoporosis Genetics Consortium (AOGC) was also included for analysis. These controls were female participants with high or low bone mass who were otherwise self-reported to be healthy.

### Whole exome sequencing and calling

Whole exome sequencing was completed using exon capture and enrichment with SureSelect Human All Exon V4 (Agilent, Santa Clara) kit as per the manufacturer’s protocol. DNA libraries were sequenced on Illumina^®^ HiSeq2000 with 100bp paired-end reads employing Macrogen^®^ Next Generation Sequencing Services. Local glaucoma-free controls were sequenced using the same capture, enrichment and platform to serve as both technical and phenotypic controls. AOGC exome data was captured with Nimblegen Human Exome Capture V2 (Roche, Basel, Switzerland), and sequenced on the HiSeq2000 (Illumina, San Diego, USA) as described previously.[19] Raw experimental data were called jointly with AOGC controls to allow a greater level of quality control across exome capture platforms. Alignment of raw reads was performed with the human genome build hg19 using novoalign (version 3.02.08). Picard's MarkDuplicates (version 1.124) software was used to filter duplicate reads. The Genome Analysis Toolkit[20] (GATK version 3.2–2) was used to conduct local indel realignment and base quality recalibration. Single nucleotide variants (SNV) and small indels were called with the UnifiedGenotyper module in GATK and variant quality scores were recalibrated according to the GATK "Best Practices Guidelines".[21] Public domain databases including refGene, SIFT, PolyPhen2 HumVAR, Exome Sequencing Project (ESP), 1000 Genomes and Exome Aggregation Consortium (ExAC) were utilized to annotate called variants using the ANNOVAR[22] software.

### Data analysis

Post-sequencing data processing and filtering was performed using in-house UNIX scripts (available on request). In order to focus on the influence of rare coding mutations, multi-stage variant filtering was performed to remove all non-coding variants, followed by all common variants and variants predicted not to be damaging by both the SIFT[23] and PolyPhen-2 HumVAR[24] software. The HumVAR version of PolyPhen-2 has a lower false positive rate and was chosen for high sensitivity of its predictions. Variants with minor allele frequency (MAF) greater than or equal to 0.1% in dbSNP v142 (www.ncbi.nlm.nih.gov/SNP/), NHLBI GO Exome Sequencing Project (ESP) v2 (evs.gs.washington.edu/), 1000 genomes v2014 (http://www.1000genomes.org/) or ExAC v3 (http://exac.broadinstitute.org/) public domain databases were defined as common. Rare canonical LoF variants in exonic regions (i.e. nonsense, splice site and frameshift mutations) were not subjected to pathogenicity filtering. The stringent control MAF cut-off of 0.1% within reference public domain databases was used in this study to limit the findings to truly rare and high penetrant variants. Typically, more common variant would have been detectable by previous GWAS.[10] PLINK[25] was used to calculate allele frequencies and perform the final quality control filtering based on Hardy Weinberg Equilibrium (p > 0.05) and internal MAF (< 0.01).

**Fig 2:**
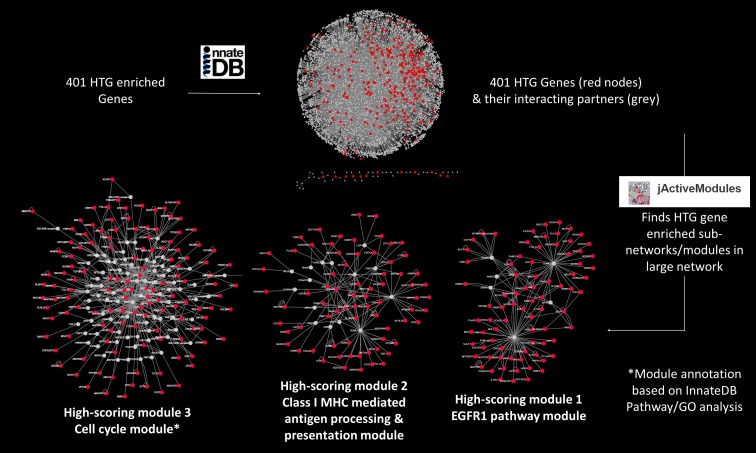
Flowchart showing network analysis using InnateDB using High-Tension Glaucoma (HTG) enriched genes as an example.

Publicly available Exome Aggregation Consortium (ExAC)[26] v3 data were annotated using the ANNOVAR pipeline and filtered using the in-house UNIX scripts. The non-Finnish European subgroup in ExAC was used for MAF filtering as the closest approximation of the population ethnicity in the current study cohort. The study cohort was divided into HTG, NTG and all POAG for analysis. Mutation burden was calculated per gene for each cohort by dividing the sum of minor allele counts for all qualifying variants by the average number of captured alleles for those variants, thus adjusting for capture rate. Furthermore, two hierarchies of variant analysis were applied, the first using only canonical LoF variants and the second utilizing both canonical LoF and predicted pathogenic variants, henceforth referred to as the LoF and predicted pathogenic models respectively. Odds ratios (OR) of the mutation burden between the case cohorts and each control cohort (local only, local plus AOGC and public ExAC data) were calculated. Genes lacking qualifying variants in the 103 screened local controls and showing enrichment of rare variants based on OR of mutation burden were selected for Gene Ontology, pathway and network analysis using InnateDB[27] (www.innatedb.com) for each comparison between case cohorts (HTG, NTG and all POAG) and all control groups. InnateDB is a publicly available platform incorporating major public domain pathway databases (including KEGG, Reactome, PID, Netpath and INOH). The database contains all human and mouse genes with their associated pathways and interactions. There is also improved annotation of the innate immunity interactome via manual curation. Genes that contained any qualifying variant in the 103 screened controls were excluded from the analysis to account for the unscreened nature of AOGC controls and variable capture between local and AOGC participants. Hypergeometric distribution tests implemented in InnateDB were used to identify statistically enriched pathways among genes enriched for rare variants in POAG, and in the HTG and NTG sub-groups independently. P-values were adjusted using the Benjamini and Hochberg method.[27] Network biology is a rapidly developing area of research, which recognises that biological processes are not chiefly controlled by individual proteins or discrete, unconnected linear pathways but rather by a complex system-level network of molecular interactions.[28] InnateDB[27] was used to construct two different networks of the experimentally validated molecular interactions that are annotated to occur between genes enriched in HTG or NTG (or the encoded products of those genes) and their first neighbour interactors. Redundant edges, self-interactions and interactions involving the highly promiscuous interactor ubiquitin C (UBC) were removed from the network. The resulting network was visualized using Cytoscape v3.4.0.[29] The networks were analyzed using the jActiveModules plugin[30] to identify high-scoring sub-networks in the larger networks that were both densely connected and enriched in either NTG or HTG associated genes (Fig 2). The parameters for the analysis were: the number of modules = 5; overlap threshold = 0.3 and search depth = 2. This type of analysis can aid in the identification of functionally relevant groups of enriched genes that may be acting in concert. High-scoring enriched sub-networks were identified and analyzed using the InnateDB ontology and pathway analysis tools to investigate whether these sub-networks were enriched in particular pathway components or functional gene categories.

## Results

Whole exome capture and massively parallel sequencing was conducted on 122 unrelated participants with HTG, 65 with NTG and 103 controls without glaucoma (Fig 1). Twenty-four *MYOC* positive participants met the inclusion criteria but were excluded from exome sequencing as the cause of their POAG was known. Variants were called with 993 AOGC unscreened controls, hence the total number of controls in this study was 1096. Age of diagnosis in the HTG group was significantly younger (p < 0.001) than in the NTG group (Table 1). Otherwise there were no significant clinical differences between the two POAG subgroups. A total of 14,783 genes contained predicted pathogenic variants and 6087 of these genes contained canonical LoF variants in either case or control cohorts.

**Table 1:** Clinical detail of POAG participants. The Mann-Whitney U test was used to assess statistical significance. HTG = high-tension glaucoma, NTG = normal-tension glaucoma, IOP = intraocular pressure, MD = mean deviation, CDR = cup-to-disc ratio, CCT = central corneal thickness.

Group	HTG		NTG		P-value
	Mean	SD	Mean	SD	
IOP (mmHg)	31.5	0.74	18.1	0.35	-
MD (dB)	-18.16	0.84	-16.35	1.05	0.183
CDR	0.901	0.009	0.894	0.014	0.631
CCT (micron)	525.0	4.15	516.5	4.97	0.088
Age at diagnosis (Years)	42.5	0.89	47.9	1.29	<0.001

Sequencing quality in both the glaucoma case and local control cohorts was high. A mean of 99.4% of all reads were mappable to the reference human genome hg19 with more than 44M on-target reads per sample. All targeted exonic regions were well covered with 97.9% of targets reaching at least 10-fold coverage and a mean depth of 73 reads per target base. The AOGC controls had a lower mean depth of 24 reads per target base and 10-fold coverage of 75.1% of targets. The number of coding region SNPs and indels per sequenced sample was similar to published exome data from 1000 Genome Project[31] at 19,605 and 465, respectively. The average variant per sample for each filtering step is shown in Table 2.

**Table 2:** Mean number of variants remaining at each stage of post-sequencing filtering.

All called variants per participant		
	Cases	Controls
Single nucleotide variants	66318	66181
Indels	6057	6017
**Filtered coding variants**		
Single nucleotide variants	19591	19632
Indels	466	462
**Filtered qualifying variants**		
Predicted pathogenic model	75.3	75.7
Canonical loss of function model	9.0	8.9

**Fig 3:**
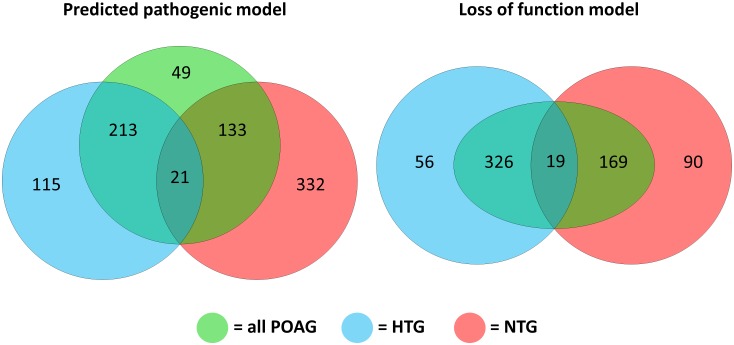
Venn diagram showing number of genes enriched in high-tension glaucoma, normal-tension glaucoma and all primary open-angle glaucoma cohorts compared with each of the control cohorts (local, AOGC and ExAC).

For the predicted pathogenic model, the mean number of qualifying variants (mutational burden) was not different between cases and controls (local and AOGC) at 75.3 per participant in the case cohort and 75.7 per participant in the control cohort (p > 0.05). Similarly, for the canonical LoF only model, the mean number of qualifying variants (mutational burden) was not different between cases and controls at 9.0 per participant in the case cohort compared to 8.9 per participant in the control cohort (p > 0.05). Genes were designated as enriched if the ORs between the case cohort and control cohort (local and AOGC) as well as the case cohort and public domain non-Finnish European ExAC cohort were greater than 5 for the LoF Model and OR greater than 4 for the predicted pathogenic model. These OR thresholds for variant enrichment were selected for the pathways analysis, because they generated an optimal number of genes for inclusion in the pathway analysis. The Venn diagram in Fig 3 illustrates the number of enriched genes included in pathway analysis from HTG, NTG and combined POAG cases, and the degree of overlap between these gene lists. The detailed gene lists are presented in S1 to S6 Tables.

Gene Ontology analysis under a predicted pathogenic model showed significant over-representation of rare variants in camera-type eye development genes in all POAG cases combined—Gene Ontology Accession GO:0043010 (p = 5.36×10^−7^, corrected p = 1.1×10^−3^). Eleven enriched genes were included in this category (Table 3), with predicted pathogenic mutations in these genes present in 10.16% (19/187) of all POAG cases and 0.73% (12/1096) of all controls (OR = 10.22 (4.87–21.43), p = 1.59×10^−9^). Negative regulation of cardiac muscle cell apoptotic process—GO:0010667 (p = 1.27×10^−5^, corrected p = 0.015) was the other significantly enriched Gene Ontology term. This category contained only four enriched genes—*HAND2*, *NKX2-5*, *PDPK1* and *SFRP2*. A similar Gene Ontology analysis using the LoF model in all POAG cases combined failed to highlight any significantly over-represented terms.

**Table 3:** Significantly enriched Gene Ontology and biological pathways in POAG and its sub-types. LoF = Loss of function, OR = Odds ratio, CI = confidence interval.

Biological mechanism	P-value	Corrected p-value	Total genes in pathway	OR(95% CI)	Enriched genes (cases vs controls)
**POAG (predicted pathogenic) gene ontology**					
Camera-type eye development	1.40×10^−7^	3.28×10^−4^	67	10.22(4.87–21.43)	*CRYBA4; GAS1; GJA8; HES5; MAB21L2; NEUROD4; NR2E1; PAX6; RXRA; SLC25A25; VAX1*
Negative regulation of cardiac muscle cell apoptotic process	1.27×10^−7^	0.015	8	15.03 (2.89–78.04)	*HAND2; NKX2-5; PDPK1; SFRP2*
**HTG (LoF) pathway analysis**					
IRE1alpha activates chaperones	7.72×10^−5^	0.013	50	76.84 (9.53–619.91)	*ACADVL; KDELR3; SHC1; SRPRB; SYVN1; TATDN2; TPP1*
XBP1(S) activates chaperone genes	5.90×10^−5^	0.019	48	76.84 (9.53–619.91)	*ACADVL; KDELR3; SHC1; SRPRB; SYVN1; TATDN2; TPP1*
Unfolded Protein Response (UPR)	2.92×10^−4^	0.032	81	87.21(10.95–694.66)	*ACADVL; EXOSC3; KDELR3; SHC1; SRPRB; SYVN1; TATDN2; TPP1*
**NTG (LoF) pathway analysis**					
Ion channel transport	1.05×10^−4^	0.027	169	17.93 (7.30–44.03)	*ATP2C2; ATP8B4; ATP9A; ATP9B; BEST3; CLCN1; GABRR2; TRPC3; TRPM8; TRPV1*

No established biological pathway or gene ontology term was significantly enriched under the predicted pathogenic model for HTG and NTG. However, in the HTG dataset, LoF mutations were significantly enriched in key regulators in the unfolded protein response (UPR) pathway—Reactome Accession R-HSA-381119 (p = 2.92×10^−4^, corrected p = 0.032) (Table 3). The other significant pathways were “IRE1alpha activates chaperones” (Reactome:R-HSA-381070) and “XBP1(S) activates chaperone genes” (Reactome:R-HSA-381038). The XBP1(S) pathway is a subgroup of the IRE1alpha signaling pathway, which itself is a main component of the UPR. LoF mutations in the eight identified UPR genes were present in 7.37% (9/122) of all HTG cases compared to 0.82% (1/1096) of all controls (OR = 87.21 (10.95–694.66), p = 7.08×10^−9^).

The only significantly over-represented pathway in the NTG cohort was ion channel transport—Reactome: R-HSA-983712 (p = 1.05×10^−4^, corrected p = 0.027) (Table 3). Several classes of transporters were included in this classification including calcium, chloride and phospholipid transporters involved in transmembrane potential maintenance and homeostasis. LoF mutations in the ten identified ion channel transport genes were present in 15.38% (10/65) of the NTG cohort and 0.91% (11/1096) of local controls (OR = 17.93 (7.30–44.03), p = 3.28×10^−8^). Mutations in genes of all three significantly enriched pathways were carried by 19.25% (36/187) of POAG cases as well as 2.19% (24/1096) of all controls (OR = 10.65 (6.18–18.34), p = 6.01×10^−17^) (Table 3).

**Fig 4:**
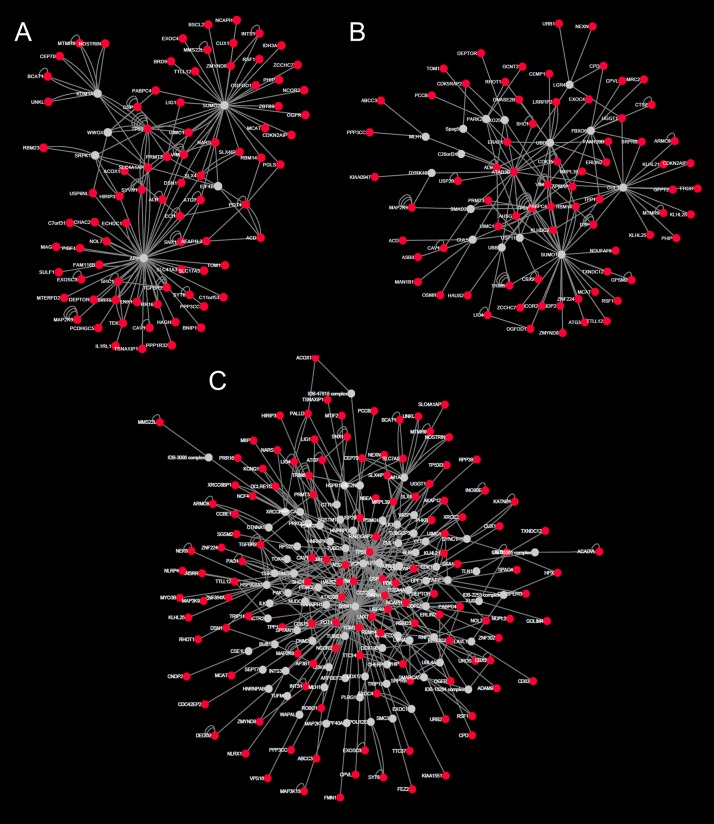
Major sub-networks/modules enriched in the high-tension glaucoma cohort. A: module 1 genes were significantly enriched for the *EGFR1* pathway. B: module 2 genes were significantly enriched for the Class I MHC mediated antigen processing & presentation pathway. C: module 3 genes were significantly enriched for the cell cycle pathway.

**Fig 5:**
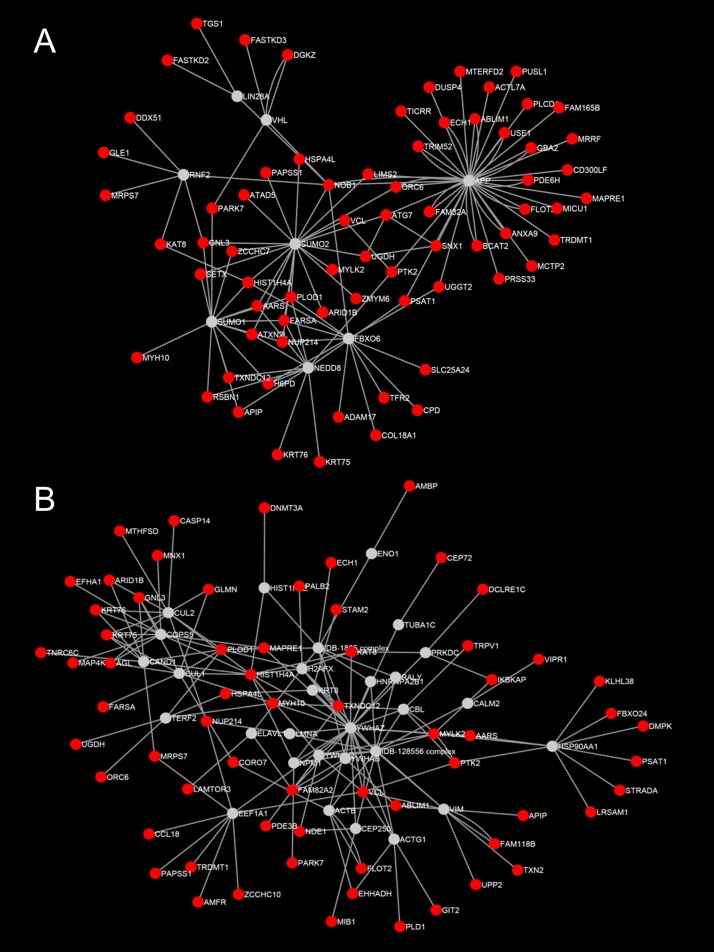
Major sub-networks/modules enriched in normal-tension glaucoma cohort. A: module 1 genes were not significantly enriched for any known biological pathways. B: module 2 genes were significantly enriched for the *EGFR1* and cell cycle pathways.

InnateDB.com[27] was used to construct the HTG and NTG networks representing the annotated molecular interactions between HTG or NTG enriched genes (or the encoded products of those genes) and their first neighbor interactors (i.e. those genes, proteins or RNAs that are annotated by InnateDB to interact directly with the enriched genes). The HTG network consisted of 5196 nodes and 10524 edges (S1 Fig). The NTG network consisted of 3748 nodes and 7134 edges (S2 Fig). Sub-network analysis of the HTG network identified 3 high-scoring modules (Fig 4): HTG module 1 consisted of 87 nodes and 178 edges; HTG module 2 consisted of 88 nodes and 161 edges and HTG module 3 consisted of 210 nodes and 488 edges. Pathway analysis revealed that the top ranked pathways associated with genes in HTG module 1, 2 and 3 was the *EGFR1* pathway (FDR < 0.01), the Class I major histocompatibility complex (MHC) mediated antigen processing & presentation pathway (FDR < 0.01), and the cell cycle pathway (FDR = 4.2×10^−9^), respectively. Sub-network analysis of the NTG network identified two major high-scoring modules (Fig 5): NTG module 1 (78 nodes and 123 edges) and NTG module 2 (94 nodes and 163 edges). No specific pathways were identified as being statistically enriched among genes in NTG module 1. Module 2 was identified, however, as being enriched in genes in the *EGFR1* pathway and in cell cycle related genes (FDR < 0.01) suggesting that similar processes may be involved in both NTG and HTG.

## Discussion

Using a rare variant approach, this study identified several biological processes which likely contribute to pathogenesis of POAG. IOP data from the ANZRAG database allowed for sub-analysis to further distinguish its role in POAG. NTG was distinguished from HTG purely on the basis of an arbitrary IOP cut-off often used in the literature (<22mmHg) without consideration of other potential phenotypic discriminators. More participants with HTG satisfied the inclusion criteria of advanced glaucoma. The two POAG subgroups had similar clinical parameters with the exception of IOP and age at diagnosis. HTG was diagnosed earlier than NTG in participants included in this study. This may be a reflection of a more rapid disease progression seen in HTG. However, the difference may be due to recruitment bias as IOP is the most accessible ocular parameter in glaucoma diagnosis. As such, it is likely that HTG is detected and diagnosed earlier in the disease course than NTG, resulting in a difference in the age at diagnosis.

### Primary open-angle glaucoma enriched genes

Previous studies have verified the contribution of *CYP1B1*, a gene that causes congenital glaucoma with high IOP, to juvenile and adult-onset POAG in various populations including Asian,[32] Australian[33] and Middle Eastern[34] ethnicities. Here we report that genes involved in camera-type eye development that are significantly enriched for rare variants in POAG, a condition which is intimately linked to congenital glaucoma. *GJA8* and *CRYBA4* are both crystalline lens-associated genes implicated in the formation of cataract. While it is well known that cataracts can contribute to the pathogenesis of angle-closure glaucoma, pathway analyses with GWAS SNPs[35] have identified associated SNPs in genes *CDK4PS*, *NFYAP1*, and *LGMNP1* shared between the POAG and cataract phenotypes, suggesting a potential genetic connection between these conditions. *GJA8* has been linked to ocular developmental abnormalities of microcornea[36] and microphthalmia[37], both of which may be related to glaucoma. The qualifying variants found in our study were different to the variants associated with microcornea and microphthalmia and unsurprisingly so, as our participants were screened to ensure the absence of any other ocular co-morbidity. Furthermore, one *GJA8* variant (p.(Asn190Ser)) has been reported in POAG cases and two *CRYBA4* variants (p.(Ser128Phe) and p.(Glu138Gly)) reported in primary angle-closure glaucoma in a Chinese cohort of 257 participants[38]. Other identified eye development genes have roles in neuronal and/or anterior segment development. Certain genes in the eye development ontology such as *PAX6* (Peter’s anomaly), *VAX1* (microphthalmia) and *MAB21L2* (syndromic microphthalmia) are linked to glaucoma-associated congenital ocular pathologies (www.omim.org). GWAS have shown that common variants near *RXRA*[39] are associated with central corneal thickness and *PAX6*[40] with optic disc area in various ethnicities including Caucasians. *PAX6* mutations cause aniridia which has a strong association with glaucoma development.[41] All mutations highlighted by the predicted pathogenic model are heterozygous and may represent a subtle form of congenital disease that only becomes observable in adulthood. The current results suggest that congenital glaucoma, whether *CYP1B1* related or not, and early adult-onset POAG may be different manifestations of the same disease continuum with contrasting severity.

### High-tension glaucoma enriched genes

The UPR and sub-classifications of this pathway were the only group of significantly enriched genes detected in the HTG cohort. A candidate gene study of common SNPs within UPR genes also revealed an association with POAG in general.[42] This pathway is involved in the pathogenesis of *myocilin* glaucoma,[43, 44] a form of POAG with exceedingly high IOP. Under normal physiological conditions, Myocilin protein is cleaved within the endoplasmic reticulum (ER) of trabecular meshwork (TM) cells and secreted into the aqueous humor to mediate cell adhesion and migration.[45] *MYOC* mutants form heterodimers with the wildtype protein that are less soluble and therefore retained within the ER.[46] The biological cascade that follows from such an accumulation of misfolded proteins activates the UPR and has been established in in-vitro human TM cells[44] and *in-vivo* transgenic *Drosophila* model overexpressing mutant *Myoc*.[43] When invoked, the effects of UPR can be summarized into three main actions that counter ER stress via three sensor proteins—IRE1, ATF6 and PERK.[45] One compensatory response is to lessen protein production via PERK-mediated inhibition of all mRNA translation. Concurrently, molecular chaperone transcription is stimulated via IRE1 and ATF6 signaling, which leads to increased solubility of misfolded proteins. IRE1 activation also induces translation of proteins involved in ER-associated protein degradation to lower the mutant protein load. If all compensatory mechanisms are overwhelmed by the accumulation of misfolded proteins, as in the case of *MYOC* mutants, then apoptosis is triggered via ATF6 and PERK signaling amongst others. Apoptosis of TM cells is recognized to contribute to IOP elevation and leads to the development of POAG.[47]

All but one of the UPR LoF mutations found in the HTG cohort are in genes involved in the IRE1 signaling pathway. The *EXOSC3* gene, while not a component of the IRE1 signaling, has a complementary role and is involved in ribonucleic acid degradation. The potential consequences of these mutations include a reduced rate of chaperone production and ER-associated protein degradation that are crucial to curtailing ER stress. Suppression of IRE1 signaling would lead to an unchecked accumulation of misfolded proteins, driving upregulation of ATF6 and PERK signaling, both of which initiate apoptosis. Molecular chaperones provide a feasible targeted therapy for managing HTG due to the ease of application. Two such substances, phenylbutyrate (PBA)[48, 49] and trimethylamine *N*-oxide (TMAO),[50] have been examined and found to be efficacious in treating *MYOC* mutants in-vitro and in animal models. Both PBA[48] and TMAO[50] were successful in improving *MYOC* mutant protein folding, solubility and in turn cell survival in transfected human TM cells. Despite having normally functioning UPR pathways, *Myoc* mutant transgenic mice develop POAG like their human counterparts due to an overwhelming misfolded protein load. PBA has displayed *in-vivo* efficacy in lowering IOP and increasing TM cell survival when administered orally[49] and topically[51] in these transgenic mice.

We have shown that rare LoF mutations in UPR genes are associated with glaucoma in a cohort of advanced HTG patients. These findings suggest that functional deficiencies in the UPR mechanism would render it incapable of clearing misfolded proteins that are generated in normal cellular metabolism even in the absence of any extraneous load such as that from *MYOC* mutants. Our findings extend the relevance of the UPR pathway and the therapeutic potential of topical molecular chaperones to include non-*MYOC*-related HTG given that all cases with pathogenic *MYOC* mutations were excluded from this study. When excluded *MYOC* positive participants are taken into account, a total of 22.6% (33 out of 146) of all HTG may be related to protein misfolding, and hence potentially amenable to molecular chaperone therapy.

### Normal-tension glaucoma enriched genes

The maintenance of transmembrane ion gradient is essential for the health and functioning of neurons such as retinal ganglion cells (RGC). Neuronal cell death can be triggered by large disruptions to this electrochemical balance as seen in the example of glutamate-associated excitotoxicity.[52] Previous experimental studies have demonstrated that addition of glutamate to retina in animal models triggers apoptosis via an intracellular calcium surge.[52] Furthermore, intracellular calcium itself can trigger neuronal apoptosis via calcineurin activation, endonuclease-mediated DNA degradation, reactive oxygen species generation by phospholipases and loss of phospholipid asymmetry via inhibition of aminophospholipid translocase.[53] Therefore, inadequate maintenance of calcium concentration and transmembrane ion balance could be a cause of RGC apoptosis in glaucoma.

The ten genes with LoF mutations in the NTG cohort consisted of transporters of a range of substrates including chloride, phospholipid, calcium and other cations. *ATP2C2*, *TRPC3*, *TRPM8* and *TRPV1* are calcium and cation channels. *TRPV1* knockout mice exhibit increased RGC susceptibility and enhanced axonal degeneration following IOP elevation.[54] Conversely, activation of *TRPV1* may protect against NMDA-induced calcium-mediated RGC apoptosis.[55] *BEST3*, *CLCN1* and *GABRR2* are chloride channels. Their involvement in the homeostasis of transmembrane electrochemical potential may contribute to suppression of voltage-gated calcium channels thereby increasing resistance to intracellular calcium surge. *ATP8B4*, *ATP9A* and *ATP9B* are active transporters of phospholipid molecules. These three genes belong to the family of aminophospholipid translocases responsible for internalizing aminophospholipid phosphatidylserine.[56] In normal cells, phospholipid asymmetry is maintained such that phosphatidylserine is almost exclusively on the intracellular side of the phospholipid bilayer by aminophospholipid translocases. Physiologic externalization of phosphatidylserine occurs in the neural retina and the process of phosphatidylserine-mediated phagocytosis has recently been shown to be the key mechanism for the diurnal recycling of photoreceptor outer segments in the retina in a mouse model.[57] Dysfunction of these translocases or their suppression by abundant intracellular calcium disrupts the phospholipid asymmetry and may incorrectly mark the affected cell for phagocytosis.[53] Knockout of an aminophospholipid translocase in the same family as the transporters identified in this study (*ATP8A2*) causes increased phagocytosis and reduced viability of photoreceptor cells in the mouse.[58] The findings of the current study shed light on the possible role of ion gradient and plasma membrane asymmetry homeostasis in regulating retinal ganglion cell survival.

### Sub-network analysis enriched pathways

Network analysis of HTG and NTG enriched genes revealed three significantly associated pathways: HTG with Class I MHC antigen processing and presentation; both HTG and NTG with *EGFR1* and cell cycle pathways. It is worth noting that these pathways were not identified as statistically significant in the pathway analysis of all HTG or NTG genes, highlighting the power of the network biology approach to uncover signatures in the data that otherwise would be overlooked. Various immune response pathways have been implicated in the pathogenesis of POAG.[59] Class I MHC antigen presentation on the surface of a cell triggers its apoptosis via activation of cytotoxic T lymphocytes. However, MHC class I molecules are only expressed on the plasma membrane of neurons in the ONH under inflammatory conditions and not under normal physiological conditions.[59] This mechanism may be important in HTG as elevated IOP may subject the ONH ganglion cells to inflammation and the expression of MHC class I molecules. Therefore any abnormalities in MHC class I presentation in HTG patients may be a crucial trigger of their RGC apoptosis. Cell cycle pathways are often regarded as central to the cascade of RGC death in POAG.[60] Our GWAS hits of *TMCO1* and *CDKN2B-AS1* in glaucoma are both genes related to the cell cycle.[10] Additionally, functional experimental studies have demonstrated that cell cycle genes are the most up-regulated genes in animal models of ONH damage via elevated IOP and ON crush injury.[60] These findings suggest that cell cycle pathways are involved in both HTG and NTG as supported by the outcomes of our network analysis. The role of *EGFR1* in glaucoma is as yet unknown, but it is well studied in human cancers and linked to cell cycle, proliferation and survival.[61] Based on our results in both HTG and NTG, *EGFR1* may be implicated in glaucoma pathogenesis via its influence on RGC survival.

### Experimental design

The main limitation of this study design is the relatively small sample size. While this sample is underpowered to detect significant single gene effects, phenotypic enrichment for severe disease and precise endophenotype characterization in this study allowed for significant findings using a system-based analysis approach. Extreme phenotypic enrichment is a great advantage of the ANZRAG database and this study, which has served well in past GWAS discoveries with relatively few samples.[10, 11] A technical limitation of this study is the variable capture between our experimental data, jointly called AOGC and public domain ExAC data. Joint-calling of local and AOGC data removed much experimental artefact that may contribute to false positives. Sequencing-related inconsistencies persisted due to incomplete coverage at some regions in the AOGC cohort that were covered well in the cases and local controls. The analysis took this into consideration by correcting for capture rate. Public domain ExAC controls were utilized as a secondary check to further limit false discoveries. Moreover, the conservative step of requiring consensus of odds ratios between cases and all controls for pathway analysis was implemented to minimize type-I errors. All measures aimed at reducing type-I error likely resulted in reduced power in the analysis. However, the robustness of system-levels analysis was able to overcome this limitation and achieve sufficient power for the detection of three biologically plausible pathways of importance in POAG. Our findings warrant further functional investigation and replication in an independent cohort of POAG cases, which was beyond the scope of this study.

In this study, rare variant investigation using whole exome sequencing has highlighted key mechanisms that contribute to glaucoma pathogenesis, complementing many decades of linkage and candidate functional work. Differing biologic mechanisms may underlie POAG with varying IOP characteristics although considerable overlap also exists. POAG may arise from abnormalities in ocular development that increase susceptibility to disease later in life with cell cycle pathways likely playing a major role. HTG is significantly associated with mutations in the UPR pathway that neutralizes protein misfolding and abnormal Class I MHC antigen presentation. Potential therapeutic chaperones targeting UPR pathway have shown promising results in in-vitro and animal *in-vivo* experiments. Mutations in ion channel transport genes significantly predispose to the development of NTG. Both pathways warrant replication in subsequent studies and ultimately further functional investigation in human POAG cohorts. Future studies with a larger whole exome sequenced cohort may be able to isolate single genes that contain rare variants associated with POAG.

## Supporting information

S1 Fig Network of all high-tension glaucoma enriched genes showing interaction between the enriched genes and their first neighbor interactors. (PDF)

S2 Fig Network of all normal-tension glaucoma enriched genes showing interaction between the enriched genes and their first neighbor interactors. (PDF)

S1 Table List of enriched genes for POAG cohort under a predicted pathogenic model. (PDF)

S2 Table List of enriched genes for POAG cohort under a loss of function model. (PDF)

S3 Table List of enriched genes for high-tension glaucoma cohort under a predicted pathogenic model. (PDF)

S4 Table List of enriched genes for high-tension glaucoma cohort under a loss of function model. (PDF)

S5 Table List of enriched genes for normal-tension glaucoma cohort under a predicted pathogenic model. (PDF)

S6 Table List of enriched genes for normal-tension glaucoma cohort under a loss of function model. (PDF)

## References

[1] Pascolini D, Mariotti SP. Global estimates of visual impairment: 2010. The British journal of ophthalmology. 2012;96(5):614–8. Epub 2011/12/03. 10.1136/bjophthalmol-2011-300539 22133988

[2] Charlesworth J, Kramer PL, Dyer T, Diego V, Samples JR, Craig JE, et al The path to open-angle glaucoma gene discovery: endophenotypic status of intraocular pressure, cup-to-disc ratio, and central corneal thickness. Investigative ophthalmology & visual science. 2010;51(7):3509–14. Epub 2010/03/20. 20237253 10.1167/iovs.09-4786 PMC2904007

[3] Wolfs RC, Klaver CC, Ramrattan RS, van Duijn CM, Hofman A, de Jong PT. Genetic risk of primary open-angle glaucoma. Population-based familial aggregation study. Archives of ophthalmology. 1998;116(12):1640–5. Epub 1998/12/31. 9869795 10.1001/archopht.116.12.1640

[4] Fingert JH, Robin AL, Stone JL, Roos BR, Davis LK, Scheetz TE, et al Copy number variations on chromosome 12q14 in patients with normal tension glaucoma. Human molecular genetics. 2011;20(12):2482–94. Epub 2011/03/31. 10.1093/hmg/ddr123 21447600 PMC3098731

[5] Rezaie T, Child A, Hitchings R, Brice G, Miller L, Coca-Prados M, et al Adult-onset primary open-angle glaucoma caused by mutations in optineurin. Science (New York, NY). 2002;295(5557):1077–9. Epub 2002/02/09. 10.1126/science.1066901 11834836

[6] Gemenetzi M, Yang Y, Lotery AJ. Current concepts on primary open-angle glaucoma genetics: a contribution to disease pathophysiology and future treatment. Eye (London, England). 2012;26(3):355–69. Epub 2011/12/17. 10.1038/eye.2011.309 PMC3298987 22173078

[7] Stone EM, Fingert JH, Alward WL, Nguyen TD, Polansky JR, Sunden SL, et al Identification of a gene that causes primary open angle glaucoma. Science (New York, NY). 1997;275(5300):668–70. Epub 1997/01/31. 10.1126/science.275.5300.668 9005853

[8] Stoilov I, Akarsu AN, Sarfarazi M. Identification of three different truncating mutations in cytochrome P4501B1 (CYP1B1) as the principal cause of primary congenital glaucoma (Buphthalmos) in families linked to the GLC3A locus on chromosome 2p21. Human molecular genetics. 1997;6(4):641–7. Epub 1997/04/01. 9097971 10.1093/hmg/6.4.641

[9] Awadalla MS, Fingert JH, Roos BE, Chen S, Holmes R, Graham SL, et al Copy number variations of TBK1 in Australian patients with primary open-angle glaucoma. American journal of ophthalmology. 2015;159(1):124–30.e1. Epub 2014/10/07. 10.1016/j.ajo.2014.09.044 25284765 PMC4355400

[10] Burdon KP, Macgregor S, Hewitt AW, Sharma S, Chidlow G, Mills RA, et al Genome-wide association study identifies susceptibility loci for open angle glaucoma at TMCO1 and CDKN2B-AS1. Nature genetics. 2011;43(6):574–8. Epub 2011/05/03. 10.1038/ng.824 21532571

[11] Gharahkhani P, Burdon KP, Fogarty R, Sharma S, Hewitt AW, Martin S, et al Common variants near ABCA1, AFAP1 and GMDS confer risk of primary open-angle glaucoma. Nature genetics. 2014;46(10):1120–5. Epub 2014/09/01. 10.1038/ng.3079 25173105 PMC4177327

[12] Mackey DA, Hewitt AW. Genome-wide association study success in ophthalmology. Current opinion in ophthalmology. 2014;25(5):386–93. Epub 2014/07/12. 10.1097/ICU.0000000000000090 25014751

[13] Manolio TA, Collins FS, Cox NJ, Goldstein DB, Hindorff LA, Hunter DJ, et al Finding the missing heritability of complex diseases. Nature. 2009;461(7265):747–53. Epub 2009/10/09. 10.1038/nature08494 19812666 PMC2831613

[14] Bansal V, Libiger O, Torkamani A, Schork NJ. Statistical analysis strategies for association studies involving rare variants. Nature reviews Genetics. 2010;11(11):773–85. Epub 2010/10/14. 10.1038/nrg2867 20940738 PMC3743540

[15] Gorlov IP, Gorlova OY, Sunyaev SR, Spitz MR, Amos CI. Shifting paradigm of association studies: value of rare single-nucleotide polymorphisms. American journal of human genetics. 2008;82(1):100–12. Epub 2008/01/09. 10.1016/j.ajhg.2007.09.006 18179889 PMC2253956

[16] Purcell SM, Moran JL, Fromer M, Ruderfer D, Solovieff N, Roussos P, et al A polygenic burden of rare disruptive mutations in schizophrenia. Nature. 2014;506(7487):185–90. Epub 2014/01/28. 10.1038/nature12975 24463508 PMC4136494

[17] Cirulli ET, Lasseigne BN, Petrovski S, Sapp PC, Dion PA, Leblond CS, et al Exome sequencing in amyotrophic lateral sclerosis identifies risk genes and pathways. Science (New York, NY). 2015. Epub 2015/02/24. 10.1126/science.aaa3650 PMC4437632 25700176

[18] Souzeau E, Goldberg I, Healey PR, Mills RA, Landers J, Graham SL, et al Australian and New Zealand Registry of Advanced Glaucoma: methodology and recruitment. Clinical & experimental ophthalmology. 2012;40(6):569–75. Epub 2011/12/17. 22171965 10.1111/j.1442-9071.2011.02742.x

[19] Estrada K, Styrkarsdottir U, Evangelou E, Hsu YH, Duncan EL, Ntzani EE, et al Genome-wide meta-analysis identifies 56 bone mineral density loci and reveals 14 loci associated with risk of fracture. Nature genetics. 2012;44(5):491–501. Epub 2012/04/17. 10.1038/ng.2249 22504420 PMC3338864

[20] McKenna A, Hanna M, Banks E, Sivachenko A, Cibulskis K, Kernytsky A, et al The Genome Analysis Toolkit: a MapReduce framework for analyzing next-generation DNA sequencing data. Genome research. 2010;20(9):1297–303. Epub 2010/07/21. 10.1101/gr.107524.110 20644199 PMC2928508

[21] Van der Auwera GA, Carneiro MO, Hartl C, Poplin R, Del Angel G, Levy-Moonshine A, et al From FastQ data to high confidence variant calls: the Genome Analysis Toolkit best practices pipeline. Curr Protoc Bioinformatics. 2013;11(1110):11.0.1-.0.33. Epub 2014/11/29. 10.1002/0471250953.bi1110s43 PMC4243306 25431634

[22] Wang K, Li M, Hakonarson H. ANNOVAR: functional annotation of genetic variants from high-throughput sequencing data. Nucleic acids research. 2010;38(16):e164 Epub 2010/07/06. 10.1093/nar/gkq603 20601685 PMC2938201

[23] Kumar P, Henikoff S, Ng PC. Predicting the effects of coding non-synonymous variants on protein function using the SIFT algorithm. Nat Protoc. 2009;4(7):1073–81. Epub 2009/06/30. 10.1038/nprot.2009.86 19561590

[24] Adzhubei IA, Schmidt S, Peshkin L, Ramensky VE, Gerasimova A, Bork P, et al A method and server for predicting damaging missense mutations. Nat Methods. 2010;7(4):248–9. Epub 2010/04/01. 10.1038/nmeth0410-248 20354512 PMC2855889

[25] Purcell S, Neale B, Todd-Brown K, Thomas L, Ferreira MA, Bender D, et al PLINK: a tool set for whole-genome association and population-based linkage analyses. American journal of human genetics. 2007;81(3):559–75. Epub 2007/08/19. 10.1086/519795 17701901 PMC1950838

[26] Lek M, Karczewski KJ, Minikel EV, Samocha KE, Banks E, Fennell T, et al Analysis of protein-coding genetic variation in 60,706 humans. Nature. 2016;536(7616):285–91. Epub 2016/08/19. 10.1038/nature19057 27535533 PMC5018207

[27] Lynn DJ, Winsor GL, Chan C, Richard N, Laird MR, Barsky A, et al InnateDB: facilitating systems-level analyses of the mammalian innate immune response. Mol Syst Biol. 2008;4:218 Epub 2008/09/04. 10.1038/msb.2008.55 18766178 PMC2564732

[28] Charitou T, Bryan K, Lynn DJ. Using biological networks to integrate, visualize and analyze genomics data. Genet Sel Evol. 2016;48:27 10.1186/s12711-016-0205-1 27036106 PMC4818439

[29] Shannon P, Markiel A, Ozier O, Baliga NS, Wang JT, Ramage D, et al Cytoscape: a software environment for integrated models of biomolecular interaction networks. Genome research. 2003;13(11):2498–504. 10.1101/gr.1239303 14597658 PMC403769

[30] Ideker T, Ozier O, Schwikowski B, Siegel AF. Discovering regulatory and signalling circuits in molecular interaction networks. Bioinformatics. 2002;18 Suppl 1:S233–40. 12169552 10.1093/bioinformatics/18.suppl_1.s233

[31] Abecasis GR, Auton A, Brooks LD, DePristo MA, Durbin RM, Handsaker RE, et al An integrated map of genetic variation from 1,092 human genomes. Nature. 2012;491(7422):56–65. Epub 2012/11/07. 10.1038/nature11632 23128226 PMC3498066

[32] Su CC, Liu YF, Li SY, Yang JJ, Yen YC. Mutations in the CYP1B1 gene may contribute to juvenile-onset open-angle glaucoma. Eye (London, England). 2012;26(10):1369–77. Epub 2012/08/11. 10.1038/eye.2012.159 PMC3470042 22878448

[33] Souzeau E, Hayes M, Zhou T, Siggs OM, Ridge B, Awadalla MS, et al Occurrence of CYP1B1 Mutations in Juvenile Open-Angle Glaucoma With Advanced Visual Field Loss. JAMA Ophthalmol. 2015;133(7):826–33. Epub 2015/05/08. 10.1001/jamaophthalmol.2015.0980 25950505

[34] Abu-Amero KK, Morales J, Aljasim LA, Edward DP. CYP1B1 Mutations are a Major Contributor to Juvenile-Onset Open Angle Glaucoma in Saudi Arabia. Ophthalmic genetics. 2013. Epub 2013/10/09. 10.3109/13816810.2013.841961 24099281

[35] Hu T, Darabos C, Cricco ME, Kong E, Moore JH. Genome-wide genetic interaction analysis of glaucoma using expert knowledge derived from human phenotype networks. Pac Symp Biocomput. 2015:207–18. Epub 2015/01/17. 25592582 PMC4299930

[36] Hu S, Wang B, Zhou Z, Zhou G, Wang J, Ma X, et al A novel mutation in GJA8 causing congenital cataract-microcornea syndrome in a Chinese pedigree. Molecular vision. 2010;16:1585–92. Epub 2010/09/02. 20806042 PMC2927419

[37] Xia CH, Chang B, Derosa AM, Cheng C, White TW, Gong X. Cataracts and microphthalmia caused by a Gja8 mutation in extracellular loop 2. PloS one. 2012;7(12):e52894 Epub 2013/01/10. 10.1371/journal.pone.0052894 23300808 PMC3530494

[38] Huang X, Xiao X, Jia X, Li S, Li M, Guo X, et al Mutation analysis of the genes associated with anterior segment dysgenesis, microcornea and microphthalmia in 257 patients with glaucoma. International journal of molecular medicine. 2015;36(4):1111–7. Epub 2015/08/28. 10.3892/ijmm.2015.2325 26310487

[39] Lu Y, Vitart V, Burdon KP, Khor CC, Bykhovskaya Y, Mirshahi A, et al Genome-wide association analyses identify multiple loci associated with central corneal thickness and keratoconus. Nature genetics. 2013;45(2):155–63. Epub 2013/01/08. 10.1038/ng.2506 23291589 PMC3720123

[40] Springelkamp H, Mishra A, Hysi PG, Gharahkhani P, Hohn R, Khor CC, et al Meta-analysis of Genome-Wide Association Studies Identifies Novel Loci Associated With Optic Disc Morphology. Genetic epidemiology. 2015;39(3):207–16. Epub 2015/01/30. 10.1002/gepi.21886 25631615 PMC4480365

[41] Jordan T, Hanson I, Zaletayev D, Hodgson S, Prosser J, Seawright A, et al The human PAX6 gene is mutated in two patients with aniridia. Nature genetics. 1992;1(5):328–32. Epub 1992/08/01. 10.1038/ng0892-328 1302030

[42] Carbone MA, Chen Y, Hughes GA, Weinreb RN, Zabriskie NA, Zhang K, et al Genes of the unfolded protein response pathway harbor risk alleles for primary open angle glaucoma. PloS one. 2011;6(5):e20649 Epub 2011/06/10. 10.1371/journal.pone.0020649 21655191 PMC3105107

[43] Carbone MA, Ayroles JF, Yamamoto A, Morozova TV, West SA, Magwire MM, et al Overexpression of myocilin in the Drosophila eye activates the unfolded protein response: implications for glaucoma. PloS one. 2009;4(1):e4216 Epub 2009/01/17. 10.1371/journal.pone.0004216 19148291 PMC2615221

[44] Yam GH, Gaplovska-Kysela K, Zuber C, Roth J. Aggregated myocilin induces russell bodies and causes apoptosis: implications for the pathogenesis of myocilin-caused primary open-angle glaucoma. The American journal of pathology. 2007;170(1):100–9. Epub 2007/01/04. 10.2353/ajpath.2007.060806 17200186 PMC1762699

[45] Anholt RR, Carbone MA. A molecular mechanism for glaucoma: endoplasmic reticulum stress and the unfolded protein response. Trends Mol Med. 2013;19(10):586–93. Epub 2013/07/24. 10.1016/j.molmed.2013.06.005 23876925 PMC3795998

[46] Gobeil S, Rodrigue MA, Moisan S, Nguyen TD, Polansky JR, Morissette J, et al Intracellular sequestration of hetero-oligomers formed by wild-type and glaucoma-causing myocilin mutants. Investigative ophthalmology & visual science. 2004;45(10):3560–7. Epub 2004/09/29. 15452063 10.1167/iovs.04-0300

[47] Sacca SC, Pulliero A, Izzotti A. The dysfunction of the trabecular meshwork during glaucoma course. J Cell Physiol. 2015;230(3):510–25. Epub 2014/09/13. 10.1002/jcp.24826 25216121

[48] Yam GH, Gaplovska-Kysela K, Zuber C, Roth J. Sodium 4-phenylbutyrate acts as a chemical chaperone on misfolded myocilin to rescue cells from endoplasmic reticulum stress and apoptosis. Investigative ophthalmology & visual science. 2007;48(4):1683–90. Epub 2007/03/29. 17389500 10.1167/iovs.06-0943

[49] Zode GS, Kuehn MH, Nishimura DY, Searby CC, Mohan K, Grozdanic SD, et al Reduction of ER stress via a chemical chaperone prevents disease phenotypes in a mouse model of primary open angle glaucoma. J Clin Invest. 2011;121(9):3542–53. Epub 2011/08/09. 10.1172/JCI58183 21821918 PMC3163970

[50] Jia LY, Gong B, Pang CP, Huang Y, Lam DS, Wang N, et al Correction of the disease phenotype of myocilin-causing glaucoma by a natural osmolyte. Investigative ophthalmology & visual science. 2009;50(8):3743–9. Epub 2009/02/24. 19234343 10.1167/iovs.08-3151

[51] Zode GS, Bugge KE, Mohan K, Grozdanic SD, Peters JC, Koehn DR, et al Topical ocular sodium 4-phenylbutyrate rescues glaucoma in a myocilin mouse model of primary open-angle glaucoma. Investigative ophthalmology & visual science. 2012;53(3):1557–65. Epub 2012/02/14. 22328638 10.1167/iovs.11-8837 PMC3339917

[52] Almasieh M, Wilson AM, Morquette B, Cueva Vargas JL, Di Polo A. The molecular basis of retinal ganglion cell death in glaucoma. Progress in retinal and eye research. 2012;31(2):152–81. Epub 2011/12/14. 10.1016/j.preteyeres.2011.11.002 22155051

[53] Orrenius S, Zhivotovsky B, Nicotera P. Regulation of cell death: the calcium-apoptosis link. Nat Rev Mol Cell Biol. 2003;4(7):552–65. Epub 2003/07/03. 10.1038/nrm1150 12838338

[54] Ward NJ, Ho KW, Lambert WS, Weitlauf C, Calkins DJ. Absence of transient receptor potential vanilloid-1 accelerates stress-induced axonopathy in the optic projection. The Journal of neuroscience: the official journal of the Society for Neuroscience. 2014;34(9):3161–70. Epub 2014/02/28. 24573275 10.1523/JNEUROSCI.4089-13.2014 PMC3935081

[55] Sakamoto K, Kuroki T, Okuno Y, Sekiya H, Watanabe A, Sagawa T, et al Activation of the TRPV1 channel attenuates N-methyl-D-aspartic acid-induced neuronal injury in the rat retina. Eur J Pharmacol. 2014;733:13–22. Epub 2014/04/08. 10.1016/j.ejphar.2014.03.035 24704373

[56] Takatsu H, Baba K, Shima T, Umino H, Kato U, Umeda M, et al ATP9B, a P4-ATPase (a putative aminophospholipid translocase), localizes to the trans-Golgi network in a CDC50 protein-independent manner. The Journal of biological chemistry. 2011;286(44):38159–67. Epub 2011/09/15. 10.1074/jbc.M111.281006 21914794 PMC3207472

[57] Ruggiero L, Connor MP, Chen J, Langen R, Finnemann SC. Diurnal, localized exposure of phosphatidylserine by rod outer segment tips in wild-type but not Itgb5-/- or Mfge8-/- mouse retina. Proceedings of the National Academy of Sciences of the United States of America. 2012;109(21):8145–8. Epub 2012/05/09. 10.1073/pnas.1121101109 22566632 PMC3361434

[58] Coleman JA, Zhu X, Djajadi HR, Molday LL, Smith RS, Libby RT, et al Phospholipid flippase ATP8A2 is required for normal visual and auditory function and photoreceptor and spiral ganglion cell survival. Journal of cell science. 2014;127(Pt 5):1138–49. Epub 2014/01/15. 10.1242/jcs.145052 24413176 PMC3937779

[59] Rieck J. The pathogenesis of glaucoma in the interplay with the immune system. Investigative ophthalmology & visual science. 2013;54(3):2393–409. Epub 2013/03/30. 23539162 10.1167/iovs.12-9781

[60] Jakobs TC. Differential gene expression in glaucoma. Cold Spring Harb Perspect Med. 2014;4(7):a020636 Epub 2014/07/06. 10.1101/cshperspect.a020636 24985133 PMC4066643

[61] Fromm JA, Johnson SA, Johnson DL. Epidermal growth factor receptor 1 (EGFR1) and its variant EGFRvIII regulate TATA-binding protein expression through distinct pathways. Molecular and cellular biology. 2008;28(20):6483–95. Epub 2008/08/20. 10.1128/MCB.00288-08 18710943 PMC2577416

