# Supplementary material for: Whole exome sequencing implicates eye development, the unfolded protein response and plasma membrane homeostasis in primary open-angle glaucoma
Source: PLoS One. 2017 Mar 6;12(3):e0172427. doi: 10.1371/journal.pone.0172427 (PMC5338784; doi:10.1371/journal.pone.0172427)
Supplement: S1 Fig — (PDF) [file pone.0172427.s001.pdf]

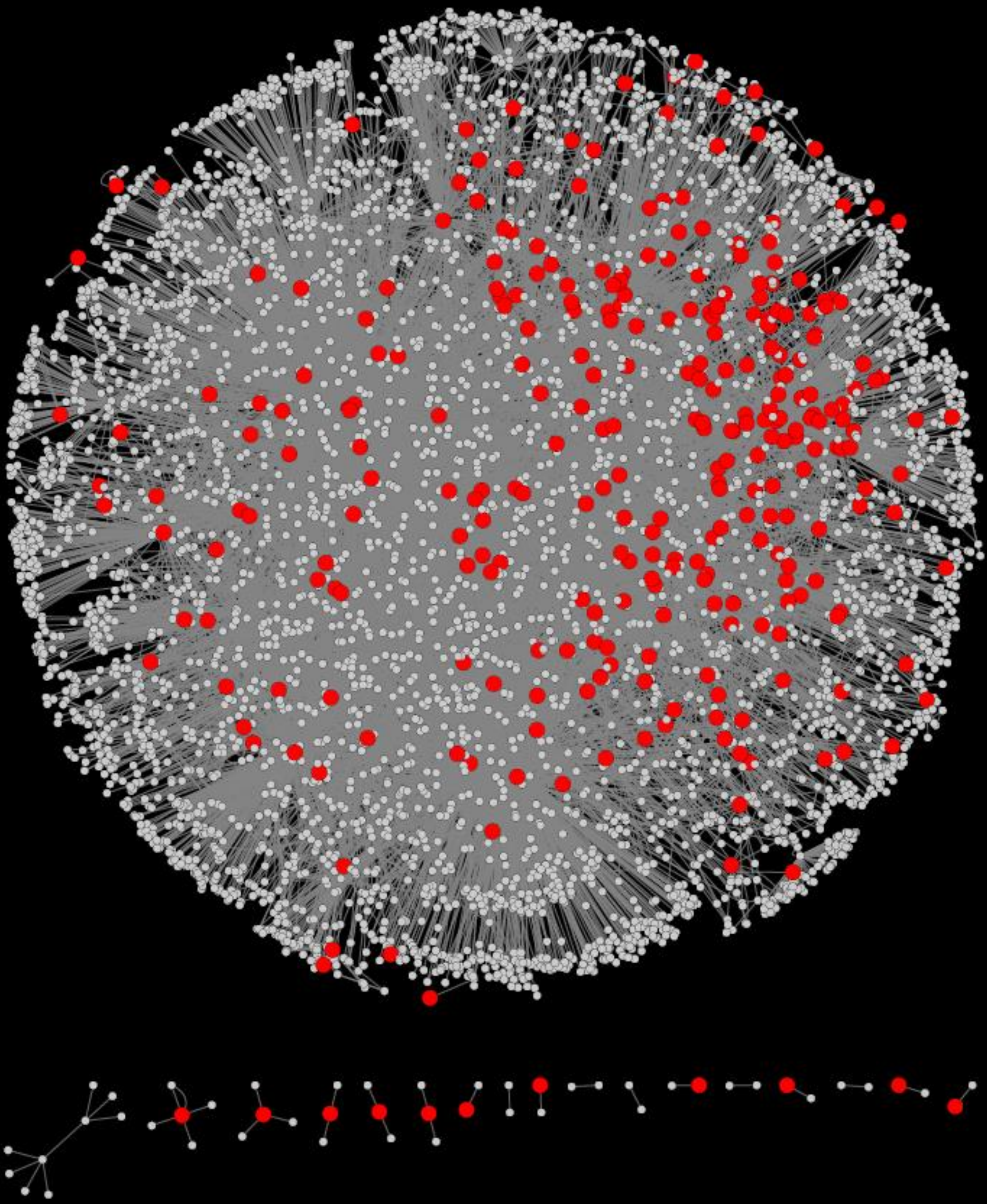

S1 Fig: Network of all high-tension glaucoma enriched genes showing interaction between the enriched genes and their first neighbor interactors
