# Supplementary material for: Whole exome sequencing implicates eye development, the unfolded protein response and plasma membrane homeostasis in primary open-angle glaucoma
Source: PLoS One. 2017 Mar 6;12(3):e0172427. doi: 10.1371/journal.pone.0172427 (PMC5338784; doi:10.1371/journal.pone.0172427)
Supplement: S2 Fig — (PDF) [file pone.0172427.s002.pdf]

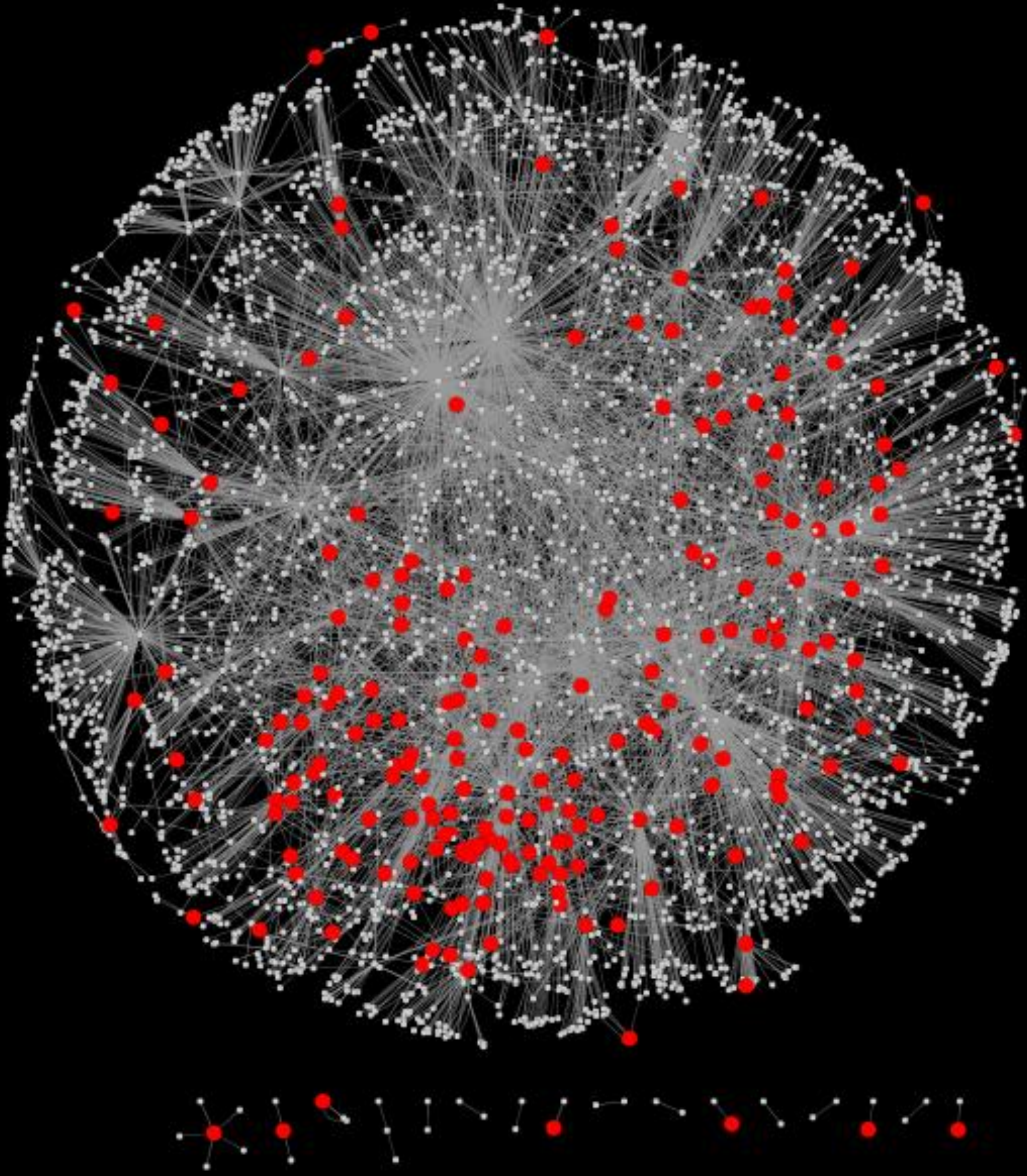

S2 Fig: Network of all normal-tension glaucoma enriched genes showing interaction between the enriched genes and their first neighbor interactors
