# Supplementary material for: Whole exome sequencing implicates eye development, the unfolded protein response and plasma membrane homeostasis in primary open-angle glaucoma
Source: PLoS One. 2017 Mar 6;12(3):e0172427. doi: 10.1371/journal.pone.0172427 (PMC5338784; doi:10.1371/journal.pone.0172427)
Supplement: S1 Table — (PDF) [file pone.0172427.s003.pdf]

S1 Table: List of enriched genes for POAG cohort under a predicted pathogenic model

Headings:
Gene: HGNC gene name
POAG: Number of cases in POAG cohort
CTRL: Number of cases in local and AOGC controls
POAG CTRL OR (95%CI): Odds ratio of POAG cohort compared to controls
POAG NFE OR (95% CI): Odds ratio of POAG cohort compared to non-Finnish European ExAC public domain data

Gene	POAG	CTRL	POAG CTRL OR (95%CI)	POAG NFE OR (95% CI)
ABHD6	1	0	Inf	4.16 (0.57-30.34)
ACBD5	2	2	5.86 (0.82-41.89)	4.76 (1.16-19.55)
ACD	5	2	13.31 (2.56-69.11)	4 (1.63-9.82)
ACTA1	1	0	Inf	8 (1.07-59.64)
AES	1	0	Inf	8.21 (1.07-62.79)
AGO1	1	1	5.88 (0.37-94.36)	5.07 (0.69-37.21)
AGPAT3	1	1	5.55 (0.35-89.06)	5.01 (0.68-36.76)
AGPAT6	1	1	5.88 (0.37-94.45)	4.15 (0.57-30.29)
AKTIP	1	1	5.88 (0.37-94.45)	4.69 (0.64-34.36)
ALDOC	3	4	4.44 (0.99-20.01)	5.51 (1.73-17.55)
AMMECR1L	1	0	Inf	11.12 (1.47-84.26)
ANAPC10	1	0	Inf	13.78 (1.79-105.87)
ANGPT2	3	2	8.91 (1.48-53.7)	7.43 (2.32-23.78)
ANKRD34A	1	0	Inf	5.27 (0.72-38.81)
ANKRD46	1	0	Inf	9.76 (1.29-73.74)
AP1AR	3	4	4.44 (0.99-20.02)	6.32 (1.98-20.19)
AP1S3	1	1	5.88 (0.37-94.45)	5.19 (0.71-38.11)
APOH	2	2	5.91 (0.83-42.19)	5.34 (1.3-21.95)
APOM	1	0	Inf	6.41 (0.87-47.41)
ARHGAP25	2	2	5.91 (0.83-42.2)	5.69 (1.38-23.44)
ARL14EPL	3	0	Inf	5.83 (1.38-24.58)
ARL5B	1	1	5.88 (0.37-94.45)	6.59 (0.89-48.79)
ATF2	1	1	5.88 (0.37-94.41)	5.01 (0.68-36.77)
ATG3	2	0	Inf	7.6 (1.83-31.53)
ATP5I	1	1	5.27 (0.33-84.7)	4.44 (0.61-32.51)
ATP6AP1L	1	0	Inf	5.11 (0.7-37.52)
ATP6V1G1	1	1	5.88 (0.37-94.36)	5.23 (0.7-39.31)
B3GNT7	1	0	Inf	4.65 (0.63-34.07)
BAD	3	1	13.73 (1.42-132.73)	4.14 (1.3-13.14)
BHLHA9	1	0	Inf	Inf
BLOC1S4	1	0	Inf	7.42 (0.98-56.49)
BMPR1A	2	1	11.83 (1.07-131.1)	5.06 (1.23-20.78)
BPIFA1	1	0	Inf	3.94 (0.54-28.77)
BTBD3	1	1	5.11 (0.32-82.11)	4.13 (0.57-30.16)
BZW2	1	0	Inf	6.3 (0.85-46.64)
C10orf95	2	0	Inf	25.25 (5.56-114.73)
C11orf86	1	1	5.46 (0.34-87.72)	17.98 (1.12-288.68)
C11orf91	1	0	Inf	3.89 (0.35-43.14)
C15orf48	1	1	5.88 (0.37-94.36)	12.48 (1.63-95.4)
C17orf105	1	1	5.88 (0.37-94.45)	7.51 (0.78-72.51)
C1orf226	1	1	5.84 (0.36-93.8)	4.03 (0.55-29.73)
C1orf52	1	1	5.15 (0.32-82.63)	6.66 (0.9-49.49)
C20orf62	3	0	Inf	8 (1.9-33.74)
C3orf14	2	1	11.82 (1.07-131.04)	18.77 (4.34-81.17)
C4A	2	0	Inf	25.18 (5.68-111.56)
C4orf32	2	0	Inf	8.97 (2.13-37.79)
C6orf120	2	0	Inf	6.7 (1.61-27.86)
C6orf52	1	0	Inf	7.89 (0.82-76.2)
C8orf37	1	0	Inf	5.1 (0.7-37.45)

CA13	2	2	5.89 (0.82-42.09)	4.33 (1.06-17.74)
CACNG4	1	1	5.74 (0.36-92.25)	5.58 (0.76-41.1)
CADM2	1	0	Inf	8.08 (1.08-60.28)
CALU	1	1	5.88 (0.37-94.45)	5.08 (0.69-37.38)
CAMK1	3	2	8.91 (1.48-53.68)	4.22 (1.33-13.38)
CATSPER3	3	3	5.93 (1.19-29.63)	4.61 (1.45-14.64)
CBFB	1	0	Inf	9.44 (1.26-70.85)
CBLL1	2	1	11.83 (1.07-131.1)	10.87 (2.59-45.65)
CCDC179	1	0	Inf	Inf
CCDC6	1	1	5.88 (0.37-94.45)	5.27 (0.72-38.75)
CCDC94	1	1	5.85 (0.36-93.93)	5.31 (0.72-39.2)
CCRN4L	1	0	Inf	5.59 (0.76-41.14)
CCSAP	1	0	Inf	7.23 (0.97-54.05)
CDC23	1	1	5.88 (0.37-94.45)	7.11 (0.96-52.78)
CDKN2AIP	3	1	17.84 (1.85-172.41)	9.5 (2.94-30.65)
CEBPD	1	0	Inf	8.96 (1.18-68.19)
CHAMP1	2	2	5.91 (0.83-42.2)	13.83 (3.26-58.69)
CHMP1B	1	0	Inf	5.9 (0.79-43.75)
CHP1	1	0	Inf	19.88 (2.51-157.71)
CHRAC1	1	0	Inf	16.68 (2.1-132.36)
CHST2	2	1	8.55 (0.77-94.81)	10.5 (2.48-44.47)
CLCN3	2	1	11.83 (1.07-131.1)	4.54 (1.11-18.62)
CLEC4D	2	2	5.91 (0.83-42.19)	9.49 (2.27-39.65)
CLPSL2	2	1	10.52 (0.95-116.57)	11.72 (2.68-51.34)
CLVS1	1	0	Inf	7.74 (1.04-57.6)
COQ10B	1	0	Inf	6.79 (0.92-50.29)
COQ5	2	1	11.82 (1.07-130.98)	4.25 (1.04-17.42)
COX17	1	0	Inf	42.21 (4.7-379.45)
COX5A	2	0	Inf	5.62 (1.37-23.14)
COX6A1	1	1	5.31 (0.33-85.3)	5.19 (0.71-38.15)
CPLX2	1	1	5.32 (0.33-85.39)	7.28 (0.97-54.65)
CPOX	2	1	4.68 (0.42-51.91)	4.83 (1.18-19.84)
CREG1	1	1	5.88 (0.37-94.36)	4.73 (0.64-34.67)
CRIPT	1	1	5.88 (0.37-94.45)	5.22 (0.71-38.3)
CRYBA4	3	2	8.9 (1.48-53.66)	7.39 (2.31-23.66)
CST3	1	0	Inf	12.24 (1.52-98.35)
CST6	2	2	4.16 (0.58-29.7)	60.24 (11.61-312.48)
CST7	2	2	5.53 (0.77-39.51)	10.9 (2.6-45.75)
CTDSP1	2	0	Inf	12.96 (3.05-55.1)
CTXN3	1	1	5.88 (0.37-94.45)	13.79 (1.79-105.95)
CYP46A1	1	1	5.88 (0.37-94.45)	6.33 (0.85-47.06)
DCAF5	4	2	11.95 (2.17-65.69)	6.08 (2.22-16.66)
DCD	1	1	5.88 (0.37-94.45)	4.57 (0.62-33.66)
DCK	1	0	Inf	7.4 (1-54.96)
DCTN3	1	0	Inf	5.27 (0.72-38.8)
DCTN5	1	0	Inf	9.23 (1.22-69.69)
DDRGK1	3	0	Inf	4.22 (1.33-13.41)
DEFB135	3	2	8.91 (1.48-53.69)	8.43 (2.63-27.09)
DESI2	1	0	Inf	19.13 (2.41-151.74)
DGCR6L	3	3	4.12 (0.82-20.58)	5.16 (1.62-16.45)

DNAJA2	1	1	5.88 (0.37-94.36)	4.44 (0.61-32.43)
DNAJC11	2	1	11.82 (1.07-131.01)	4.75 (1.16-19.49)
DPF2	1	1	5.88 (0.37-94.45)	5.66 (0.77-41.64)
DPM1	1	1	5.88 (0.37-94.36)	5.59 (0.76-41.12)
DRG1	1	0	Inf	8.94 (1.19-66.97)
DYDC1	2	1	11.83 (1.07-131.1)	14.29 (3.36-60.77)
DYNC1I1	2	2	5.91 (0.83-42.2)	5.06 (1.23-20.81)
DYNLRB1	1	0	Inf	16.33 (2.08-128.25)
EBF3	2	2	5.89 (0.82-42.05)	7.13 (1.72-29.56)
ECHDC3	3	1	11.69 (1.21-113.04)	4.4 (1.39-13.96)
EEF1E1	1	0	Inf	9.64 (1.28-72.81)
EGFL8	2	2	5.53 (0.77-39.53)	4.02 (0.98-16.47)
EID1	1	0	Inf	8.26 (1.11-61.74)
EIF2S1	1	0	Inf	6.37 (0.86-47.08)
EIF4H	1	0	Inf	4.38 (0.6-32)
EMID1	2	0	Inf	5.16 (1.25-21.28)
ERICH2	3	0	Inf	13.84 (2.77-69.03)
ERLIN2	2	0	Inf	12.85 (3.04-54.32)
EVI2A	1	0	Inf	3.96 (0.54-28.86)
FAM110A	2	0	Inf	5.32 (1.28-22.06)
FAM131A	2	2	5.64 (0.79-40.27)	4.44 (1.08-18.23)
FAM181B	1	0	Inf	5.23 (0.69-39.47)
FAM32A	2	2	5.91 (0.83-42.19)	9.61 (2.24-41.18)
FAM89B	1	0	Inf	9.04 (1.2-67.89)
FBXO21	2	1	4.71 (0.42-52.26)	6.34 (1.53-26.27)
FBXO22	2	1	10.54 (0.95-116.79)	6.03 (1.46-24.96)
FBXW12	3	3	5.93 (1.19-29.62)	4.73 (1.49-15.03)
FGF9	1	0	Inf	16.26 (2.09-126.62)
FGL2	3	4	4.44 (0.99-20)	5.79 (1.82-18.45)
FNTA	1	1	5.88 (0.37-94.41)	5.5 (0.75-40.61)
FOXD2	1	0	Inf	7.29 (0.96-55.24)
FOXG1	1	0	Inf	13.11 (1.67-102.9)
FOXH1	2	2	5.42 (0.76-38.7)	4.57 (1.11-18.82)
FOXO6	1	0	Inf	5.96 (0.54-66.06)
FRAT1	1	1	5.63 (0.35-90.39)	6.6 (0.85-51.04)
FST	1	0	Inf	6.18 (0.84-45.59)
FXYD4	1	0	Inf	5.43 (0.74-39.9)
FXYD7	1	0	Inf	25.6 (3.13-209.09)
GAS1	2	0	Inf	15.01 (3.27-68.98)
GATAD1	1	0	Inf	5.27 (0.72-38.74)
GDF1	2	0	Inf	4.93 (1.08-22.39)
GDF10	2	2	5.88 (0.82-42.04)	4.62 (1.12-19.01)
GDNF	1	1	5.88 (0.37-94.41)	4.5 (0.61-33)
GEMIN2	1	0	Inf	5.06 (0.69-37.13)
GFPT1	2	1	11.83 (1.07-131.1)	9.75 (2.33-40.79)
GGACT	1	0	Inf	12.47 (0.78-200.19)
GJA8	3	4	4.43 (0.98-19.95)	6.03 (1.89-19.21)
GLYR1	2	0	Inf	6.35 (1.54-26.23)
GPN2	2	1	11.56 (1.04-128.16)	4.73 (1.15-19.42)
GPR183	1	0	Inf	11.89 (1.56-90.48)

GRAMD4	2	1	11.46 (1.03-127.03)	4.51 (1.1-18.49)
GTF2B	1	1	5.88 (0.37-94.36)	4.7 (0.64-34.42)
H2AFZ	1	1	5.87 (0.37-94.19)	178.89 (11.15-2870.92)
HAND2	1	0	Inf	30.36 (3.53-261.17)
HAUS2	1	1	5.87 (0.37-94.32)	5.4 (0.73-39.73)
HBEGF	1	0	Inf	14.96 (1.92-116.44)
HCN1	1	1	5.76 (0.36-92.46)	4.03 (0.55-29.43)
HDAC3	1	0	Inf	8.53 (1.14-63.73)
HDGFL1	1	0	Inf	4.23 (0.57-31.23)
HES3	3	0	Inf	25.53 (7.46-87.42)
HES5	1	0	Inf	12.39 (1.58-97.28)
HIGD1B	1	0	Inf	7.09 (0.96-52.57)
HIST1H2AG	1	0	Inf	4.55 (0.62-33.31)
HIST1H3B	1	1	5.77 (0.36-92.59)	7.72 (1.04-57.48)
HIST1H3F	1	0	Inf	7.46 (1-55.45)
HIST1H4I	1	1	5.65 (0.35-90.74)	4.32 (0.59-31.6)
HIST2H2AB	2	0	Inf	14.78 (3.47-62.99)
HLA-DMB	1	1	5.88 (0.37-94.45)	5.39 (0.73-39.71)
HNRNPA3	1	0	Inf	24.99 (2.99-208.59)
HPGDS	2	1	11.82 (1.07-131.04)	4.49 (1.1-18.4)
HS3ST3B1	1	0	Inf	5.99 (0.8-44.68)
HSBP1	1	0	Inf	18.01 (2.16-150.33)
HTR1A	1	1	5.86 (0.36-94.1)	6.14 (0.83-45.29)
IFFO2	2	1	7.82 (0.71-86.71)	4.66 (1.1-19.82)
IGF2BP1	1	0	Inf	4.5 (0.61-32.91)
IL20RB	1	0	Inf	5.1 (0.7-37.42)
IL36A	1	0	Inf	6.64 (0.9-49.1)
IL5	1	0	Inf	6.88 (0.93-50.99)
IMMP2L	1	0	Inf	4.81 (0.66-35.24)
IMPAD1	1	1	5.88 (0.37-94.49)	5.58 (0.76-41.17)
INSL3	2	0	Inf	5.84 (1.4-24.38)
ITPA	3	1	17.83 (1.84-172.37)	11.18 (3.45-36.21)
ITPK1	3	2	7.84 (1.3-47.25)	6.86 (2.14-22.03)
JUNB	1	0	Inf	6.59 (0.88-49.35)
KAT8	1	0	Inf	7.73 (1.04-57.65)
KATNAL1	1	0	Inf	4.56 (0.62-33.33)
KBTBD4	3	3	4.27 (0.86-21.34)	5.77 (1.81-18.37)
KCNH1	3	0	Inf	7.28 (2.27-23.29)
KCNMA1	4	2	5.86 (1.07-32.28)	7.17 (2.6-19.76)
KCTD4	1	0	Inf	8.52 (1.14-63.69)
KLF13	1	0	Inf	8.31 (1.1-62.74)
KNG1	3	1	17.83 (1.84-172.36)	4.11 (1.3-13.02)
KPNB1	1	0	Inf	13.6 (1.77-104.48)
KRTAP20-1	1	0	Inf	3.97 (0.54-28.92)
KRTAP29-1	2	0	Inf	7.24 (1.32-39.79)
LAMTOR3	1	0	Inf	13.67 (1.77-105.65)
LBX1	1	1	5.78 (0.36-92.9)	4.27 (0.58-31.23)
LCN6	2	1	11.78 (1.06-130.56)	5.35 (1.3-22.03)
LEMD1	1	0	Inf	5.29 (0.72-38.91)
LGI4	4	3	4.21 (0.93-18.98)	5.46 (1.97-15.07)

LHX9	1	0	Inf	4.81 (0.66-35.25)
LMO2	1	0	Inf	6.68 (0.9-49.64)
LMO7DN	1	0	Inf	Inf
LOC730159	1	0	Inf	9.42 (0.59-151.28)
LPCAT4	3	4	4.44 (0.99-20.01)	6.45 (2.02-20.6)
LST1	2	0	Inf	5.37 (1.3-22.26)
LYPD2	2	2	5.81 (0.81-41.48)	4.3 (1.04-17.73)
MAB21L2	1	1	5.85 (0.36-93.89)	6.1 (0.83-45.02)
MAGEF1	3	2	6.25 (1.04-37.69)	11.83 (3.64-38.43)
MAP2K7	2	2	5.67 (0.79-40.49)	5.35 (1.3-22.08)
MAPKAPK2	1	1	5.62 (0.35-90.22)	6.37 (0.86-47.06)
MAPRE1	1	0	Inf	8.13 (1.09-60.62)
MB21D1	4	5	4.72 (1.26-17.76)	4.76 (1.74-12.99)
MBTD1	1	1	5.88 (0.37-94.49)	8.14 (1.09-60.82)
MEA1	1	0	Inf	5.86 (0.8-43.21)
MED19	1	0	Inf	4.83 (0.66-35.42)
MLLT3	1	0	Inf	4.95 (0.67-36.27)
MMD	1	1	5.88 (0.37-94.45)	8.01 (1.07-59.74)
MNX1	1	0	Inf	4.14 (0.56-30.84)
MORN3	2	2	5.89 (0.82-42.05)	5.62 (1.36-23.2)
MRPL12	1	0	Inf	4.33 (0.59-31.74)
MRPL17	2	2	5.86 (0.82-41.88)	11.31 (2.69-47.62)
MRPS10	3	1	17.84 (1.85-172.41)	6.57 (2.06-21)
MRPS12	1	0	Inf	7.55 (1.01-56.22)
MRPS26	2	0	Inf	6.82 (1.64-28.35)
MS4A3	1	0	Inf	4.68 (0.64-34.24)
MS4A6E	1	1	5.88 (0.37-94.49)	9.96 (1.32-74.99)
MYLK2	3	3	5.92 (1.19-29.55)	6.93 (2.16-22.2)
NACC1	2	1	10.1 (0.91-112)	12.31 (2.91-52.13)
NAPB	1	0	Inf	6.36 (0.86-47.06)
NAT14	1	1	4.59 (0.29-73.66)	10.91 (1.27-93.84)
NBL1	1	0	Inf	4.78 (0.5-46.22)
NBPF3	3	1	17.82 (1.84-172.25)	4.32 (1.36-13.7)
NDFIP2	1	1	5.5 (0.34-88.28)	10.57 (1.4-80.15)
NDNL2	2	1	11.82 (1.07-130.98)	9.2 (2.2-38.55)
NDRG3	1	1	5.88 (0.37-94.45)	4.59 (0.63-33.58)
NDUFA12	2	1	11.82 (1.07-131.06)	8.77 (2.11-36.54)
NDUFB2	1	1	5.88 (0.37-94.45)	4.61 (0.63-33.91)
NDUFC2-KCTD14	1	0	Inf	Inf
NECAP2	2	0	Inf	7.45 (1.8-30.92)
NEUROD4	2	2	5.91 (0.83-42.2)	5.89 (1.43-24.25)
NFKBIL1	2	0	Inf	6.89 (1.66-28.67)
NGB	3	0	Inf	21.8 (6.3-75.46)
NKX2-5	1	0	Inf	6.48 (0.87-48.55)
NKX6-2	3	0	Inf	11.01 (3.39-35.81)
NOL7	2	2	5.27 (0.74-37.64)	8.71 (2.07-36.56)
NOVA1	1	0	Inf	4.77 (0.65-35.02)
NOXRED1	2	0	Inf	5.23 (1.27-21.49)
NPAS3	3	4	4.06 (0.9-18.28)	4.35 (1.36-13.84)
NPW	1	0	Inf	4.11 (0.55-30.52)

NR2E1	1	0	Inf	12.05 (1.57-92.55)
NRL	1	0	Inf	6.82 (0.91-50.96)
NTMT1	1	0	Inf	4.23 (0.58-30.91)
NUP54	2	1	11.83 (1.07-131.1)	10.72 (2.55-45.07)
NUSAP1	4	3	7.95 (1.76-35.8)	4.95 (1.81-13.56)
ONECUT1	2	2	5.13 (0.72-36.66)	4.35 (1.06-17.82)
OR2J2	4	1	23.92 (2.66-215.2)	6.59 (2.41-18.06)
OR5R1	4	3	7.96 (1.77-35.84)	5.64 (2.06-15.42)
OSR2	1	1	5.82 (0.36-93.5)	4.1 (0.56-30.04)
OTX1	1	0	Inf	5.19 (0.71-38.11)
OTX2	1	0	Inf	6.85 (0.92-50.75)
PARL	1	0	Inf	4.05 (0.55-29.59)
PAX6	1	0	Inf	4.61 (0.63-33.78)
PBK	2	2	5.9 (0.83-42.18)	4.68 (1.14-19.18)
PDCD7	2	1	5.93 (0.53-65.78)	4.77 (1.16-19.63)
PDHB	1	1	5.88 (0.37-94.45)	4.66 (0.64-34.12)
PDPK1	2	2	5.37 (0.75-38.37)	13.22 (3.12-55.99)
PDX1	1	0	Inf	6.4 (0.85-47.91)
PDZD9	1	0	Inf	10.09 (1.34-76.22)
PELO	1	1	5.87 (0.37-94.28)	9.86 (1.31-74.25)
PEX11B	3	1	15.86 (1.64-153.32)	5.41 (1.7-17.24)
PEX3	1	0	Inf	6.34 (0.86-46.82)
PF4	2	2	5.91 (0.83-42.2)	25.79 (5.78-115.09)
PGF	3	4	4.39 (0.97-19.78)	13.84 (4.23-45.28)
PHIP	2	1	11.82 (1.07-131.06)	4.22 (1.03-17.29)
PIGC	1	0	Inf	3.96 (0.54-28.91)
PNPLA8	3	4	4.44 (0.99-20.02)	6.07 (1.9-19.36)
POLR3A	3	1	17.84 (1.85-172.41)	4.04 (1.28-12.82)
POLR3D	2	1	6.62 (0.6-73.44)	6.64 (1.61-27.47)
PPIH	2	2	5.9 (0.83-42.17)	8.97 (2.15-37.4)
PPM1L	2	1	11.83 (1.07-131.1)	10.55 (2.52-44.23)
PPP2R5A	2	1	11.82 (1.07-130.98)	8.14 (1.95-33.95)
PRLR	2	2	4.2 (0.59-30)	5.61 (1.36-23.09)
PRPSAP2	1	0	Inf	4.65 (0.63-34.04)
PRR23C	1	0	Inf	4.88 (0.65-36.29)
PSMB1	1	1	5.88 (0.37-94.45)	6.15 (0.83-45.44)
RAB13	1	1	5.88 (0.37-94.36)	5.56 (0.76-40.87)
RAB29	1	1	5.88 (0.37-94.36)	4.84 (0.66-35.5)
RAB2A	1	0	Inf	36.42 (4.05-327.43)
RAB6A	1	0	Inf	11 (1.45-83.4)
RAD21L1	3	0	Inf	23.75 (4.76-118.49)
RARRES2	1	0	Inf	6.04 (0.81-45.14)
RASL10B	1	1	5.71 (0.36-91.69)	24.57 (3.01-200.68)
RCHY1	1	1	5.71 (0.36-91.65)	4.21 (0.58-30.76)
RCN2	1	0	Inf	9.16 (1.22-68.97)
REEP3	1	1	5.88 (0.37-94.41)	9.14 (1.2-69.56)
RFX6	3	0	Inf	4.35 (1.37-13.79)
RGS17	1	0	Inf	5.17 (0.7-38)
RGS2	2	2	5.9 (0.83-42.17)	6.65 (1.61-27.47)
RLBP1	3	3	5.85 (1.17-29.2)	5.75 (1.8-18.33)

RND3	2	1	11.82 (1.07-131.06)	7.33 (1.77-30.36)
RNF141	4	4	5.96 (1.48-24.05)	38.28 (12.9-113.62)
RPL15	1	0	Inf	11.02 (1.42-85.81)
RPL18A	1	0	Inf	4.21 (0.58-30.79)
RPL5	1	1	5.88 (0.37-94.36)	5.22 (0.71-38.33)
RPL8	1	0	Inf	6.24 (0.85-46.13)
RPP38	1	1	5.88 (0.37-94.45)	4.23 (0.58-30.88)
RPRM	1	1	5.76 (0.36-92.55)	12.23 (1.6-93.45)
RRAGA	1	0	Inf	14.93 (1.93-115.4)
RSPO3	1	1	5.88 (0.37-94.45)	5.66 (0.77-41.68)
RXRA	2	0	Inf	12.15 (2.88-51.29)
SAC3D1	3	1	9.74 (1.01-94.22)	11.94 (3.65-39.08)
SAP30L	1	1	5.88 (0.37-94.45)	5.26 (0.71-38.79)
SERPINB2	2	2	5.91 (0.83-42.2)	4.18 (1.02-17.12)
SERPINF2	1	0	Inf	5.28 (0.72-38.79)
SFRP2	1	0	Inf	6.83 (0.92-50.66)
SGCZ	1	1	5.88 (0.37-94.45)	4.43 (0.61-32.43)
SHISA7	1	0	Inf	Inf
SIRT7	1	0	Inf	4.46 (0.61-32.7)
SLBP	3	0	Inf	22.61 (6.75-75.75)
SLC25A25	2	2	5.85 (0.82-41.79)	4.89 (1.19-20.09)
SLC25A48	3	2	8.91 (1.48-53.69)	11.43 (3.52-37.19)
SLC35F6	2	0	Inf	7.42 (1.79-30.83)
SLC39A14	2	1	6.6 (0.6-73.2)	4.49 (1.09-18.41)
SLC40A1	1	1	5.88 (0.37-94.45)	5.11 (0.7-37.48)
SLC48A1	1	0	Inf	5.31 (0.72-39.16)
SLX4IP	2	1	11.83 (1.07-131.1)	8.51 (2.05-35.43)
SMARCA4	4	3	6.39 (1.42-28.78)	5.02 (1.83-13.72)
SMEK2	2	2	5.87 (0.82-41.91)	4.46 (1.09-18.3)
SMIM11	1	0	Inf	19.63 (2.47-155.7)
SMIM14	2	1	11.83 (1.07-131.1)	15.63 (3.66-66.79)
SMIM18	1	0	Inf	Inf
SMR3A	1	1	5.88 (0.37-94.45)	5.96 (0.81-43.94)
SMR3B	2	2	5.91 (0.83-42.2)	4.22 (1.03-17.27)
SNAP25	1	0	Inf	16.63 (2.12-130.6)
SNN	1	0	Inf	10.4 (1.38-78.52)
SNX27	2	1	11.82 (1.07-130.98)	5.8 (1.4-23.98)
SNX8	3	2	8.63 (1.43-52.01)	12 (3.67-39.17)
SOWAHA	1	0	Inf	10.32 (1.33-80.32)
SP9	1	0	Inf	3.91 (0.48-31.97)
SPINK1	1	0	Inf	8.57 (1.14-64.22)
SPINK4	1	1	5.88 (0.37-94.45)	10.86 (1.43-82.3)
SPTY2D1	3	3	5.93 (1.19-29.61)	8.19 (2.55-26.29)
SRR	1	0	Inf	4.71 (0.64-34.48)
SRSF11	1	0	Inf	4.05 (0.56-29.57)
SRSF5	3	1	17.84 (1.85-172.41)	4.79 (1.51-15.22)
ST8SIA2	1	0	Inf	4.96 (0.68-36.46)
STAG1	1	0	Inf	4.31 (0.59-31.52)
STAM2	2	0	Inf	4.92 (1.2-20.21)
STIP1	1	0	Inf	3.89 (0.53-28.43)

STK24	1	0	Inf	5.24 (0.71-38.46)
STK4	2	1	11.83 (1.07-131.1)	7.8 (1.88-32.36)
SUPT16H	1	0	Inf	4.58 (0.63-33.54)
SVIP	1	0	Inf	4.94 (0.67-36.4)
TAC3	1	0	Inf	7.31 (0.98-54.35)
TADA2B	1	1	5.89 (0.37-94.54)	8.31 (1.11-62.24)
TAGLN3	1	0	Inf	16.25 (2.09-126.49)
TAPT1	3	3	5.93 (1.19-29.63)	8.25 (2.55-26.76)
TBC1D22A	3	4	4.17 (0.92-18.77)	4.86 (1.53-15.48)
TCTN3	5	4	7.45 (1.98-28.01)	9.88 (3.96-24.64)
TDP2	1	0	Inf	4.81 (0.66-35.27)
TGFBR2	3	1	14.18 (1.47-137.1)	6.95 (2.17-22.24)
TIRAP	2	2	5.85 (0.82-41.81)	5.6 (1.36-23.07)
TLX1	1	0	Inf	4.01 (0.54-29.46)
TMBIM1	3	2	8.91 (1.48-53.68)	5.45 (1.71-17.38)
TMEM116	2	1	11.82 (1.07-131.02)	5.88 (1.43-24.23)
TMEM14E	1	0	Inf	5.36 (0.73-39.43)
TMEM223	1	0	Inf	4.07 (0.55-30.1)
TMEM256	1	0	Inf	5.42 (0.73-40.11)
TMEM65	1	1	5.88 (0.37-94.45)	15.22 (1.94-119.5)
TMEM69	1	0	Inf	4.23 (0.58-30.91)
TMEM86A	2	2	5.88 (0.82-41.99)	6.31 (1.53-26.08)
TMEM8B	6	5	6.9 (2.09-22.86)	8.05 (3.51-18.49)
TMEM92	2	0	Inf	14.75 (3.46-62.87)
TNFRSF11B	1	1	5.88 (0.37-94.45)	6.63 (0.9-49.03)
TNFRSF25	2	1	10.57 (0.95-117.15)	6.43 (1.55-26.78)
TNFSF14	1	1	5.88 (0.37-94.41)	6.31 (0.85-46.76)
TNFSF15	1	0	Inf	5.24 (0.71-38.55)
TNFSF18	2	2	5.9 (0.83-42.16)	6.06 (1.47-24.98)
TNIP2	2	1	11.68 (1.05-129.42)	5.56 (1.35-22.93)
TPH2	2	2	5.91 (0.83-42.19)	3.99 (0.98-16.33)
TRIB1	2	1	11.83 (1.07-131.1)	4.58 (1.12-18.79)
TRIM44	1	0	Inf	4.35 (0.59-31.82)
TRMT12	2	1	11.83 (1.07-131.14)	4.91 (1.2-20.18)
TRMT5	1	0	Inf	4.13 (0.57-30.17)
TSPAN18	2	0	Inf	5.98 (1.45-24.63)
TSPAN3	1	1	5.88 (0.37-94.36)	8.78 (1.17-66.08)
TTLL1	2	0	Inf	4.41 (1.08-18.09)
TXNDC12	3	0	Inf	18.09 (5.46-59.92)
TYROBP	1	0	Inf	6.03 (0.82-44.61)
UBIAD1	1	0	Inf	9.43 (1.26-70.78)
UBP1	3	2	8.91 (1.48-53.69)	5.74 (1.8-18.3)
UBXN7	1	1	5.88 (0.37-94.45)	7.73 (1.04-57.57)
UCHL5	2	0	Inf	18.67 (4.28-81.4)
UFC1	2	0	Inf	5.28 (1.28-21.71)
UNC5D	4	2	8.45 (1.54-46.48)	4.37 (1.6-11.92)
USE1	1	0	Inf	5.03 (0.68-37.1)
UTP18	4	0	Inf	7.2 (2.62-19.8)
VAX1	1	1	5.63 (0.35-90.48)	8.2 (1.09-61.58)
VDAC3	1	1	5.88 (0.37-94.45)	4.44 (0.61-32.49)

WDYHV1	1	1	5.88 (0.37-94.45)	5.32 (0.72-39.16)
XRCC2	2	0	Inf	6.27 (1.52-25.88)
YBEY	1	0	Inf	5.71 (0.77-42.12)
YRDC	1	0	Inf	6.81 (0.91-50.9)
YWHAB	1	0	Inf	4.05 (0.55-29.53)
YWHAE	1	1	5.48 (0.34-88.06)	17.8 (2.27-139.78)
ZBED2	3	0	Inf	6.39 (2-20.4)
ZBTB34	1	0	Inf	7.68 (1.03-57.14)
ZBTB44	3	2	8.91 (1.48-53.67)	4.56 (1.43-14.49)
ZIC2	1	0	Inf	6.74 (0.9-50.39)
ZMYND19	1	0	Inf	5.34 (0.73-39.26)
ZNF146	2	1	11.84 (1.07-131.22)	15.64 (3.66-66.8)
ZNF205	3	3	5.27 (1.06-26.33)	4.72 (1.49-15)
ZNF511	2	1	11.23 (1.01-124.51)	4.24 (1.03-17.38)
ZNF716	6	0	Inf	5.04 (2.21-11.5)
ZNF768	2	1	11.38 (1.03-126.13)	7.48 (1.8-31.01)
ZPBP2	2	1	11.82 (1.07-131.02)	5.41 (1.32-22.27)
