# Supplementary material for: Whole exome sequencing implicates eye development, the unfolded protein response and plasma membrane homeostasis in primary open-angle glaucoma
Source: PLoS One. 2017 Mar 6;12(3):e0172427. doi: 10.1371/journal.pone.0172427 (PMC5338784; doi:10.1371/journal.pone.0172427)
Supplement: S2 Table — (PDF) [file pone.0172427.s004.pdf]

S2 Table: List of enriched genes for POAG cohort under a loss of function model

Headings:
Gene: HGNC gene name
POAG: Number of cases in POAG cohort
CTRL: Number of cases in local and AOGC controls
POAG CTRL OR (95% CI): Odds ratio of POAG cohort compared to controls
POAG NFE OR (95% CI): Odds ratio of POAG cohort compared to non-Finnish European ExAC public domain data

Gene	POAG	CTRL	POAG CTRL OR (95% CI)	POAG NFE OR (95%CI)
AARS	1	0	Inf	14.47 (1.87-111.82)
ABCA2	1	0	Inf	12.35 (1.57-97.01)
ABCC3	1	0	Inf	5.08 (0.69-37.3)
ABLIM1	1	1	5.87 (0.37-94.32)	18.57 (2.34-147.34)
ACADVL	2	0	Inf	20.06 (4.6-87.45)
ACD	1	0	Inf	6.54 (0.88-48.41)
ACOX1	1	0	Inf	29.84 (3.57-249.09)
ADAM17	1	0	Inf	18.41 (2.32-146.05)
ADAM9	2	0	Inf	72.04 (13.89-373.69)
ADD1	1	0	Inf	6.77 (0.91-50.3)
ADPRHL1	1	1	5.87 (0.37-94.32)	5.19 (0.71-38.14)
ADTRP	1	0	Inf	14.12 (1.78-112.03)
AFAP1L2	1	0	Inf	8.49 (1.12-64.15)
AGL	2	2	5.9 (0.83-42.16)	5.03 (1.23-20.68)
AGPAT2	1	0	Inf	16.73 (2.11-132.73)
AHSG	1	0	Inf	11.06 (1.46-83.84)
AKAP12	1	1	5.88 (0.37-94.45)	15.8 (2.03-122.97)
ALB	1	0	Inf	16.14 (2.07-125.68)
ALKBH1	1	1	5.88 (0.37-94.49)	9.43 (1.26-70.77)
AMBP	1	0	Inf	11.08 (1.46-83.95)
AMFR	1	0	Inf	35.09 (4.08-301.84)
ANKDD1A	1	0	Inf	7.13 (0.96-52.97)
ANO3	1	1	5.88 (0.37-94.45)	9.79 (1.3-73.68)
ANXA9	1	1	5.88 (0.37-94.36)	5.86 (0.8-43.21)
AP3B1	1	0	Inf	85.31 (7.7-944.96)
APIP	1	0	Inf	12.42 (1.62-94.92)
APMAP	1	1	5.88 (0.37-94.45)	16.9 (2.15-132.7)
APOBEC1	1	0	Inf	58.31 (6.04-563.14)
APOBR	1	1	5.85 (0.36-93.93)	5.01 (0.68-37.19)
APOH	1	0	Inf	17.73 (2.26-139.25)
ARHGEF25	1	0	Inf	9.42 (1.25-70.9)
ARHGEF26	1	0	Inf	4.98 (0.68-36.69)
ARID1B	1	0	Inf	21.1 (2.63-169.57)
ARMC8	1	0	Inf	79.92 (7.22-885.21)
ARRDC2	1	0	Inf	10.09 (1.33-76.46)
ARSK	1	0	Inf	18.73 (2.36-148.62)
ATAD3B	1	0	Inf	5.18 (0.71-38.09)
ATAD5	1	1	5.88 (0.37-94.45)	19.69 (2.48-156.24)
ATG3	1	0	Inf	44.34 (4.93-398.58)
ATG4D	1	0	Inf	7.03 (0.94-52.42)
ATG7	2	0	Inf	15.56 (3.64-66.46)
ATP5G2	1	0	Inf	25.42 (3.11-207.66)
ATP6V0A2	2	0	Inf	23.65 (5.37-104.14)
ATP6V1E2	1	0	Inf	13.76 (1.79-105.7)
ATP9A	1	0	Inf	35.06 (4.08-301.53)
ATXN2L	1	0	Inf	7.54 (1.01-56.26)
AVPR1A	1	1	5.67 (0.35-91.04)	25.14 (3.08-205.36)
BAAT	1	0	Inf	19.79 (2.49-157)
BCAT1	1	0	Inf	18.78 (2.37-148.99)

BCAT2	1	0	Inf	15.3 (1.97-119.11)
BDH2	1	1	5.88 (0.37-94.45)	6.42 (0.87-47.57)
BIN3	1	0	Inf	17.86 (2.25-141.67)
BRD9	1	0	Inf	35.71 (4.15-307.13)
BRF2	1	0	Inf	6.84 (0.92-50.67)
BSCL2	1	1	5.88 (0.37-94.49)	10.3 (1.36-77.79)
C11orf1	1	0	Inf	5.46 (0.74-40.23)
C11orf53	1	0	Inf	12.74 (1.67-97.35)
C12orf40	1	1	5.88 (0.37-94.45)	8.89 (1.18-66.79)
C17orf67	1	0	Inf	19.8 (2.5-157.11)
C1orf109	1	0	Inf	15.44 (1.98-120.24)
C3orf30	2	1	11.82 (1.07-131.04)	21.04 (4.83-91.73)
C7orf31	2	1	11.82 (1.07-131.06)	9.43 (2.26-39.39)
CABS1	1	0	Inf	12.69 (1.66-96.99)
CACNA1S	1	0	Inf	5.46 (0.74-40.19)
CAPRIN2	1	0	Inf	8.91 (1.19-66.76)
CASP14	1	0	Inf	8.11 (1.09-60.5)
CATSPER2	1	0	Inf	8.47 (1.13-63.26)
CATSPERG	3	1	17.8 (1.84-172.09)	5.68 (1.78-18.14)
CAV1	1	0	Inf	19.89 (2.51-157.82)
CBX2	1	0	Inf	15.86 (2.04-123.47)
CCBE1	1	0	Inf	9.91 (1.32-74.61)
CCDC57	3	1	17.74 (1.84-171.46)	13.59 (4.14-44.59)
CCL18	1	0	Inf	35.82 (4.16-308.11)
CCR5	4	3	7.96 (1.77-35.84)	9.31 (3.37-25.72)
CD200R1L	2	0	Inf	44.08 (9.3-208.97)
CD300LF	1	0	Inf	14.72 (1.9-113.81)
CD83	1	0	Inf	54.01 (5.59-521.66)
CDC42EP2	1	0	Inf	Inf
CDCP1	1	0	Inf	34.83 (4.05-299.61)
CDK15	1	0	Inf	6.83 (0.92-50.58)
CDKN2AIP	1	0	Inf	Inf
CEACAM18	1	0	Inf	11.02 (1.45-83.5)
CEMP1	1	0	Inf	58.84 (6.09-568.32)
CEP128	1	1	5.88 (0.37-94.45)	5.99 (0.81-44.28)
CEP72	1	0	Inf	5.05 (0.69-37.08)
CES3	1	0	Inf	13.55 (1.76-104.08)
CH25H	1	1	5.88 (0.37-94.49)	27.17 (3.26-226.82)
CHAC2	1	1	5.88 (0.37-94.41)	5.43 (0.74-39.93)
CHRNB2	1	0	Inf	41.94 (4.67-377.04)
CIB1	1	0	Inf	25.57 (3.13-208.87)
CLEC4D	2	0	Inf	28.82 (6.41-129.69)
CLEC4M	1	0	Inf	25.45 (3.12-207.92)
CLPSL2	1	0	Inf	48.45 (4.37-536.7)
CNBD1	1	1	5.88 (0.37-94.36)	8.78 (1.16-66.33)
CNDP2	1	0	Inf	19.82 (2.5-157.24)
COG2	1	0	Inf	28.02 (3.36-233.87)
CPD	2	0	Inf	40.02 (8.59-186.49)
CRYBB1	1	0	Inf	29.82 (3.57-248.88)
CTSH	1	0	Inf	7.08 (0.95-52.58)

CTSZ	1	0	Inf	7.74 (1.04-57.69)
CUX1	1	0	Inf	13.46 (1.75-103.46)
CYP21A2	1	0	Inf	36.69 (3.8-354.41)
CYP2E1	1	0	Inf	9.8 (1.3-73.82)
CYP46A1	1	0	Inf	89.65 (8.09-993.03)
DCLRE1C	4	1	23.92 (2.66-215.2)	15.3 (5.46-42.91)
DEDD2	1	0	Inf	4.9 (0.65-36.86)
DEFB135	1	0	Inf	8.88 (1.19-66.5)
DENND6B	1	1	5.85 (0.36-93.97)	37.32 (4.15-335.48)
DEPTOR	1	0	Inf	19.7 (2.48-156.29)
DERL3	1	0	Inf	8.92 (1.17-67.87)
DGKZ	1	0	Inf	26.36 (2.93-237)
DHX32	1	1	5.88 (0.37-94.45)	8.36 (1.12-62.51)
DKK4	1	0	Inf	22.17 (2.76-178.17)
DMRT3	1	1	5.88 (0.37-94.45)	14.91 (1.93-115.23)
DNAJC16	1	0	Inf	5.4 (0.74-39.71)
DNAJC27	1	0	Inf	35.28 (4.1-303.47)
DNASE1L3	1	0	Inf	16.2 (2.08-126.12)
DNASE2B	1	0	Inf	6.14 (0.83-45.43)
DPP6	1	1	5.88 (0.37-94.49)	23.65 (2.83-197.42)
DSN1	1	0	Inf	5.53 (0.75-40.65)
DSP	1	0	Inf	9.4 (1.25-70.59)
DUSP16	1	0	Inf	44.71 (4.97-401.89)
DUSP4	1	0	Inf	40.37 (4.49-362.93)
ECH1	1	1	5.88 (0.37-94.41)	7.81 (1.04-58.61)
ECHDC1	1	1	5.88 (0.37-94.45)	5.96 (0.81-43.92)
ECI1	1	0	Inf	8.47 (1.13-63.61)
EGFL8	1	0	Inf	21.6 (2.69-173.6)
EPG5	1	0	Inf	6.13 (0.83-45.21)
ERLIN2	1	0	Inf	89.4 (8.07-990.25)
EXOC4	1	1	5.88 (0.37-94.45)	11.85 (1.56-90.15)
EXOG	1	0	Inf	7.76 (1.04-57.74)
EXOSC3	1	0	Inf	84.82 (7.66-939.5)
FAM118B	1	0	Inf	29.78 (3.57-248.55)
FAM120B	2	0	Inf	8.65 (2.07-36.12)
FAM124A	1	1	5.88 (0.37-94.36)	18.64 (2.35-147.84)
FAM178A	1	0	Inf	10.74 (1.42-81.41)
FAM32A	1	0	Inf	132.27 (8.24-2122.7)
FAM46B	1	0	Inf	8.43 (1.13-63.16)
FAM69C	2	1	11.47 (1.03-127.15)	72.02 (13.88-373.59)
FAM71C	1	0	Inf	8.05 (1.08-60.05)
FAM71E2	1	0	Inf	Inf
FAM83B	1	0	Inf	16.25 (2.09-126.5)
FAM84B	1	0	Inf	37.89 (4.21-340.62)
FAR2	1	0	Inf	89.44 (8.07-990.67)
FARSA	1	0	Inf	28.41 (3.4-237.12)
FASTKD2	1	0	Inf	12.57 (1.64-96.09)
FBLN2	1	0	Inf	15.56 (1.96-123.47)
FBXO24	1	0	Inf	12.21 (1.59-93.84)
FBXO39	1	0	Inf	16.27 (2.09-126.64)

FCN1	2	1	11.81 (1.07-130.86)	5.17 (1.26-21.27)
FLOT2	1	0	Inf	20.95 (2.61-168.33)
FMN1	1	1	5.87 (0.37-94.32)	7.65 (1.02-57.15)
FMO5	1	0	Inf	5.23 (0.71-38.43)
FNDC9	1	0	Inf	17.66 (2.25-138.66)
FREM1	1	1	5.88 (0.37-94.41)	5.01 (0.68-36.88)
FXYD7	1	0	Inf	179.4 (11.18-2879.12)
FZD2	1	0	Inf	89.27 (8.06-988.8)
FZD6	1	0	Inf	9.32 (1.24-70)
GAL3ST3	1	0	Inf	18.15 (2.22-148.24)
GALNS	1	0	Inf	14.13 (1.82-110.04)
GALNT14	2	2	5.91 (0.83-42.2)	14.94 (3.5-63.84)
GALNT3	1	0	Inf	11.63 (1.53-88.51)
GAS2L2	1	1	5.88 (0.37-94.49)	7.88 (1.05-58.85)
GBA2	1	0	Inf	10.98 (1.45-83.21)
GCNT2	2	0	Inf	13.33 (3.15-56.45)
GFPT2	1	0	Inf	5.31 (0.72-39.06)
GIT2	1	0	Inf	42.77 (4.76-384.51)
GJA8	1	0	Inf	19.43 (2.45-154.13)
GLE1	1	1	5.88 (0.37-94.45)	10.41 (1.38-78.66)
GLIPR2	1	0	Inf	5.45 (0.74-40.1)
GML	2	0	Inf	35.93 (7.82-165.12)
GOLIM4	1	0	Inf	6.55 (0.89-48.48)
GPBAR1	1	0	Inf	7.59 (1.02-56.71)
GPR149	1	0	Inf	8.43 (1.13-63)
GPR37L1	1	0	Inf	21.81 (2.71-175.25)
GPX5	1	0	Inf	44.77 (4.98-402.51)
GRAMD2	1	0	Inf	16.27 (2.09-126.64)
GRIN2C	1	0	Inf	11.5 (1.49-88.9)
GUCA1C	1	0	Inf	19.59 (2.47-155.44)
HAGH	1	1	5.48 (0.34-88.06)	9.84 (1.31-74.13)
HAUS2	1	0	Inf	24.72 (3.03-201.96)
HCN1	1	0	Inf	35.02 (4.07-301.24)
HECW1	1	0	Inf	29.48 (3.53-246.06)
HIRIP3	1	1	5.88 (0.37-94.45)	19.68 (2.48-156.09)
HIST1H4A	1	0	Inf	7.7 (1.04-57.35)
HMGCLL1	1	0	Inf	13.26 (1.73-101.92)
HPS4	1	1	5.88 (0.37-94.45)	6.75 (0.91-50.03)
HPX	1	0	Inf	12.66 (1.66-96.8)
HSPA4L	1	1	5.88 (0.37-94.41)	15.73 (2.02-122.42)
ICE1	1	0	Inf	23.91 (2.93-195.32)
IDH3A	1	0	Inf	89.64 (8.09-992.89)
IFI44L	1	0	Inf	10.86 (1.43-82.33)
IFT88	1	0	Inf	5.66 (0.77-41.7)
IGFBP6	1	0	Inf	11.17 (1.47-84.69)
IL1RL1	1	1	5.88 (0.37-94.49)	17.75 (2.26-139.4)
IL20RB	1	0	Inf	13.69 (1.78-105.17)
IMPG2	2	0	Inf	13.58 (3.2-57.64)
INO80E	1	0	Inf	9.27 (1.18-72.8)
INSM2	1	0	Inf	29.14 (3.39-250.67)

INSRR	2	1	11.82 (1.07-130.98)	10.33 (2.46-43.36)
INTS1	1	1	5.78 (0.36-92.81)	6.8 (0.91-50.62)
INTU	1	1	5.88 (0.37-94.45)	7.41 (1-55.06)
IRGM	1	0	Inf	22.86 (1.42-366.92)
KAT8	1	0	Inf	43 (4.78-386.52)
KATNB1	1	0	Inf	14.68 (1.9-113.49)
KCNH1	1	0	Inf	25.36 (3.11-207.19)
KCNH5	2	1	11.82 (1.07-131.04)	11.07 (2.63-46.55)
KCNJ1	1	0	Inf	6.59 (0.89-48.72)
KCNJ14	2	1	11.64 (1.05-129)	53.23 (10.67-265.45)
KCNQ1	1	1	5.88 (0.37-94.41)	5.43 (0.74-39.91)
KCTD19	1	1	5.88 (0.37-94.45)	7.45 (1-55.38)
KHDC1L	1	0	Inf	7.08 (0.92-54.39)
KIAA1549	1	1	5.88 (0.37-94.45)	41.57 (4.62-373.72)
KIAA1551	1	0	Inf	5.72 (0.78-42.12)
KIF12	1	0	Inf	13.16 (1.71-101.09)
KLHDC2	1	0	Inf	12.76 (1.66-98.07)
KLHL21	1	0	Inf	32.04 (3.56-287.99)
KLHL25	1	0	Inf	7.08 (0.95-52.53)
KLHL28	1	0	Inf	171.61 (10.69-2754.1)
KLK1	1	0	Inf	9.38 (1.25-70.42)
KNG1	1	0	Inf	11.15 (1.47-84.48)
KRT75	2	2	5.91 (0.83-42.18)	5.53 (1.34-22.75)
KRT76	1	0	Inf	10.88 (1.43-82.43)
KY	1	0	Inf	10.38 (1.36-79.01)
LAMTOR3	1	0	Inf	172.6 (10.76-2770.02)
LDHAL6A	1	0	Inf	16.27 (2.09-126.67)
LDLRAD2	1	0	Inf	26.33 (3.06-226.5)
LEMD1	1	0	Inf	25.94 (3.11-216.56)
LHX4	1	0	Inf	44.78 (4.98-402.53)
LIG1	1	0	Inf	7.43 (1-55.32)
LIPI	1	0	Inf	11.56 (1.52-87.96)
LMBRD2	1	0	Inf	15.76 (2.02-122.72)
LMOD2	1	0	Inf	12.54 (1.58-99.51)
LNX1	1	1	5.88 (0.37-94.45)	5.47 (0.74-40.26)
LPIN3	3	3	5.93 (1.19-29.63)	5.89 (1.85-18.77)
LRRC23	1	0	Inf	6.83 (0.92-50.62)
LRRC66	2	0	Inf	5.41 (1.32-22.25)
LRRFIP2	1	1	5.88 (0.37-94.45)	6.62 (0.89-48.97)
LRSAM1	1	0	Inf	10.24 (1.36-77.37)
LTBP2	1	0	Inf	9.7 (1.28-73.25)
MAG	1	0	Inf	21.29 (2.65-171.1)
MAP3K13	2	0	Inf	25.41 (5.74-112.61)
MAP3K9	1	1	5.86 (0.36-94.06)	35.3 (4.1-303.65)
MAP4K3	1	0	Inf	11.56 (1.52-87.97)
MAPRE1	1	0	Inf	179.39 (11.18-2878.86)
MATN3	1	0	Inf	15.25 (1.96-118.74)
MB21D1	1	0	Inf	8.07 (1.07-60.96)
MBOAT1	1	0	Inf	9.13 (1.22-68.56)
MCAT	1	0	Inf	9.98 (1.32-75.4)

METTL4	1	0	Inf	8.8 (1.17-65.9)
MICU1	1	0	Inf	6.68 (0.9-49.83)
MICU2	1	0	Inf	12.43 (1.62-95.53)
MMS22L	1	0	Inf	14.46 (1.87-111.81)
MNX1	1	0	Inf	Inf
MOV10L1	2	2	5.91 (0.83-42.2)	8.62 (2.07-35.92)
MRC2	1	0	Inf	28.22 (3.28-242.73)
MRPL39	1	1	5.88 (0.37-94.45)	11.17 (1.47-84.63)
MRPS7	1	0	Inf	43.86 (4.88-394.27)
MRRF	1	0	Inf	12.78 (1.67-97.67)
MS4A7	1	0	Inf	44.75 (4.98-402.25)
MSR1	1	0	Inf	5.32 (0.72-39.12)
MT1F	1	0	Inf	35.83 (4.17-308.16)
MTERF4	2	0	Inf	13.3 (3.14-56.34)
MTHFS	1	0	Inf	25.62 (3.14-209.31)
MTHFSD	1	1	5.88 (0.37-94.45)	5.07 (0.69-37.37)
MTIF2	1	0	Inf	6.38 (0.86-47.12)
MTMR9	1	0	Inf	44.71 (4.97-401.89)
MUC15	1	1	5.88 (0.37-94.45)	11.02 (1.45-83.55)
MYCBPAP	1	0	Inf	12.35 (1.61-94.91)
MYH10	1	1	5.88 (0.37-94.45)	10.44 (1.38-79.17)
MYLK2	1	0	Inf	Inf
MYO16	1	0	Inf	11.68 (1.53-89.26)
NAA38	1	0	Inf	10.71 (1.41-81.14)
NARS	1	0	Inf	13.26 (1.73-101.89)
NARS2	1	0	Inf	22.31 (2.78-179.24)
NBEA	1	0	Inf	15.74 (2.02-122.54)
NCAPH	1	0	Inf	30.95 (3.6-266.17)
NCF4	2	0	Inf	5.32 (1.29-21.89)
NCOR2	1	0	Inf	6.58 (0.88-49.04)
NDE1	1	0	Inf	25.51 (3.12-208.41)
NDST4	1	1	5.88 (0.37-94.41)	29.29 (3.51-244.5)
NDUFAF6	1	0	Inf	6.72 (0.91-49.87)
NEK8	1	0	Inf	9.93 (1.32-74.74)
NEU2	1	1	5.7 (0.35-91.47)	21.68 (2.7-174.21)
NEXN	1	0	Inf	6.04 (0.82-44.58)
NGB	1	0	Inf	Inf
NLRP11	1	0	Inf	10.51 (1.39-79.36)
NLRP4	1	1	5.87 (0.37-94.28)	13.63 (1.77-104.72)
NLRX1	2	1	11.64 (1.05-129.06)	7.42 (1.79-30.73)
NMNAT3	1	0	Inf	5.09 (0.69-37.4)
NOB1	1	0	Inf	17.3 (2.2-135.82)
NOL7	1	1	5.84 (0.36-93.8)	12.83 (1.66-99.19)
NOSTRIN	1	1	5.88 (0.37-94.41)	7.4 (1-54.97)
NPBWR1	1	0	Inf	5.68 (0.77-42.14)
NPHS2	1	0	Inf	12.67 (1.66-96.85)
NPR1	1	0	Inf	13.59 (1.76-105.02)
NPTX2	1	0	Inf	171.79 (10.7-2756.94)
NRL	1	0	Inf	21.52 (2.68-172.93)
NSUN7	1	0	Inf	10.04 (1.31-77.18)

NUP214	1	0	Inf	5.39 (0.73-39.63)
NXPE1	1	0	Inf	25.36 (3.1-207.13)
OGFOD1	1	1	5.88 (0.37-94.45)	10.51 (1.39-79.41)
OGFR	1	0	Inf	14.2 (1.77-114.11)
OR10AD1	1	0	Inf	13.73 (1.79-105.53)
OR11H6	1	0	Inf	5.56 (0.76-40.92)
OR12D3	1	0	Inf	12.97 (1.69-99.62)
OR13A1	1	0	Inf	59.29 (6.14-572.64)
OR2AE1	1	0	Inf	22.09 (2.75-177.53)
OR51Q1	1	0	Inf	16.3 (2.09-126.86)
OR52E4	1	0	Inf	25.58 (3.13-208.92)
OR6Q1	1	0	Inf	16.15 (2.07-125.71)
OR6T1	1	0	Inf	16.3 (2.09-126.89)
OR9I1	1	0	Inf	12.77 (1.67-97.59)
ORC6	1	0	Inf	13.95 (1.81-107.86)
OSBPL1A	1	1	5.88 (0.37-94.45)	9.4 (1.25-70.55)
OSMR	1	0	Inf	5.56 (0.76-40.88)
OTOP3	1	0	Inf	21.69 (2.7-174.32)
OVGP1	2	0	Inf	8.42 (2.02-35.05)
OVOL1	1	0	Inf	125.31 (7.81-2011.1)
P2RX1	1	0	Inf	13.87 (1.79-107.24)
PAG1	1	0	Inf	29.66 (3.55-247.6)
PARK7	1	0	Inf	5.04 (0.69-37)
PAX4	1	0	Inf	7.09 (0.95-53.12)
PBK	1	0	Inf	9.2 (1.22-69.07)
PCCB	1	0	Inf	8.84 (1.18-66.21)
PCDHB10	1	1	5.88 (0.37-94.45)	8.01 (1.07-59.73)
PCDHGA8	1	0	Inf	10.44 (1.38-78.86)
PCDHGC5	1	1	5.88 (0.37-94.49)	12.31 (1.61-94.09)
PCNX	1	0	Inf	12.71 (1.66-97.17)
PCSK7	1	0	Inf	18.6 (2.34-147.52)
PDE6H	1	0	Inf	24.83 (3.04-202.82)
PDK4	1	0	Inf	8 (1.07-59.77)
PDZD8	1	0	Inf	178.15 (11.1-2859.01)
PGLS	1	0	Inf	31.44 (3.5-282.61)
PHIP	1	0	Inf	24.27 (2.97-198.26)
PHKB	2	0	Inf	9.09 (2.18-37.92)
PIBF1	1	0	Inf	5.36 (0.73-39.4)
PIGC	1	0	Inf	16.23 (2.08-126.37)
PIGL	1	1	5.88 (0.37-94.45)	17.92 (2.28-140.71)
PLCB4	1	0	Inf	21.83 (2.72-175.43)
PLCH1	2	0	Inf	22.23 (5.08-97.37)
PLOD1	1	0	Inf	9.17 (1.22-68.87)
PLVAP	1	1	5.88 (0.37-94.45)	59.23 (6.13-572.08)
PODN	1	0	Inf	7.31 (0.98-54.65)
POT1	1	0	Inf	9.12 (1.21-68.5)
PPP3CC	1	0	Inf	11.74 (1.54-89.37)
PRMT3	1	1	5.88 (0.37-94.49)	7.13 (0.96-52.96)
PRR16	1	0	Inf	60.79 (5.49-673.39)
PRR30	1	0	Inf	13.45 (1.75-103.34)

PRSS36	2	0	Inf	4.87 (1.18-20.02)
PSAT1	1	0	Inf	16.29 (2.09-126.78)
PTDSS2	1	0	Inf	25.39 (3.11-207.43)
PTK2	1	0	Inf	89.44 (8.07-990.64)
PUSL1	2	0	Inf	6.9 (1.64-28.98)
PYGM	1	0	Inf	6.8 (0.92-50.38)
RAB3GAP2	1	0	Inf	14.64 (1.89-113.13)
RBM14	1	0	Inf	Inf
RBM23	1	0	Inf	7.14 (0.96-53.03)
RCN3	1	1	5.79 (0.36-92.98)	10.26 (1.35-78.07)
RFX6	1	0	Inf	13.78 (1.79-105.88)
RHOT1	1	0	Inf	12.67 (1.66-96.83)
RMDN3	2	2	5.9 (0.83-42.17)	38.38 (8.24-178.84)
RNASE12	1	0	Inf	6.39 (0.87-47.24)
RNF112	1	0	Inf	50.76 (5.26-490.2)
RNF141	1	0	Inf	35.8 (4.16-307.93)
RNLS	1	0	Inf	7.44 (1-55.3)
ROBO1	1	0	Inf	8.81 (1.17-66.16)
RPP38	1	1	5.88 (0.37-94.45)	17.84 (2.27-140.07)
RRH	1	0	Inf	6.58 (0.89-48.67)
RSF1	1	0	Inf	Inf
RTN4IP1	2	0	Inf	8.92 (2.14-37.17)
SCN9A	1	0	Inf	4.98 (0.68-36.62)
SDR39U1	1	0	Inf	8.31 (1.11-62.28)
SEC22C	1	0	Inf	8 (1.07-59.66)
SELL	1	0	Inf	39.41 (4.08-380.63)
SEMA3D	1	0	Inf	17.38 (2.21-136.49)
SETD6	1	0	Inf	7.2 (0.97-53.48)
SETX	1	1	5.88 (0.37-94.45)	7.64 (1.03-56.89)
SFRP4	1	0	Inf	16.24 (2.09-126.42)
SGCZ	1	0	Inf	14.58 (1.87-113.49)
SGSM2	1	1	5.68 (0.35-91.26)	8.46 (1.13-63.35)
SH3TC2	1	1	5.88 (0.37-94.41)	6.39 (0.87-47.24)
SHB	1	0	Inf	82.82 (5.16-1329.08)
SHC1	1	0	Inf	25.44 (3.11-207.82)
SIAH3	1	0	Inf	88.92 (8.03-984.95)
SIRT5	1	1	5.88 (0.37-94.45)	9.81 (1.3-73.88)
SKA1	1	1	5.87 (0.37-94.23)	5.39 (0.73-39.65)
SLC12A9	2	1	11.59 (1.05-128.52)	15.75 (3.67-67.66)
SLC15A3	2	0	Inf	10.88 (2.59-45.75)
SLC17A5	1	0	Inf	5.23 (0.71-38.47)
SLC22A15	1	1	5.88 (0.37-94.36)	14.67 (1.88-114.19)
SLC25A26	1	1	5.88 (0.37-94.45)	6.31 (0.84-47.23)
SLC26A7	1	0	Inf	11.7 (1.54-89.02)
SLC28A3	1	0	Inf	9.36 (1.25-70.3)
SLC36A2	2	1	11.83 (1.07-131.1)	12.78 (3.02-54.05)
SLC41A3	1	0	Inf	12.54 (1.64-95.88)
SLC47A1	1	0	Inf	8.86 (1.18-66.38)
SLC4A1AP	1	0	Inf	6.62 (0.89-48.95)
SLC5A9	2	2	5.9 (0.83-42.15)	6.63 (1.6-27.38)

SLC6A5	1	0	Inf	11.2 (1.47-85.2)
SLC7A9	1	0	Inf	5.59 (0.76-41.09)
SLC9A5	1	0	Inf	5.57 (0.76-40.98)
SLX4IP	1	0	Inf	12.7 (1.66-97.11)
SMIM11	1	0	Inf	87.35 (7.89-967.57)
SMOC2	2	0	Inf	31 (6.82-140.85)
SMPD2	1	0	Inf	4.96 (0.68-36.38)
SNAPC1	1	0	Inf	19.76 (2.49-156.76)
SNX1	2	1	11.82 (1.07-130.98)	88.28 (16.07-484.98)
SPAG4	2	0	Inf	21.78 (4.95-95.93)
SPHKAP	1	0	Inf	25.51 (3.12-208.35)
SPINK1	1	0	Inf	17.61 (2.22-139.74)
SPINK4	1	0	Inf	178.81 (11.14-2869.54)
SRMS	1	0	Inf	10.96 (1.43-84.22)
SRPRB	1	0	Inf	17.77 (2.26-139.54)
ST6GALNAC2	1	0	Inf	4.89 (0.67-35.92)
STAM2	2	0	Inf	27.55 (6.17-122.93)
STEAP4	1	0	Inf	7.07 (0.95-52.49)
STRADA	1	1	5.88 (0.37-94.45)	12.47 (1.63-95.31)
STRN3	1	1	5.88 (0.37-94.36)	22.19 (2.76-178.3)
SULF1	1	0	Inf	35.68 (4.15-306.93)
SULT1B1	1	0	Inf	10.26 (1.36-77.49)
SULT1C3	1	1	5.89 (0.37-94.54)	6.83 (0.92-50.58)
SULT2A1	1	0	Inf	13.74 (1.79-105.6)
SYT5	1	1	5.88 (0.37-94.45)	5.88 (0.79-43.65)
SYT6	1	0	Inf	59 (6.11-569.8)
SYVN1	1	0	Inf	Inf
TACC3	1	0	Inf	12.59 (1.65-96.26)
TATDN2	1	0	Inf	13.56 (1.76-104.18)
TBX19	1	1	5.88 (0.37-94.36)	7.77 (1.04-57.81)
TCF12	1	0	Inf	35.78 (4.16-307.79)
TEDDM1	1	0	Inf	17.9 (2.28-140.52)
TEK	1	0	Inf	89.42 (8.07-990.48)
TFR2	2	0	Inf	26.32 (5.85-118.45)
TGFBR2	1	0	Inf	59.49 (6.16-574.6)
TGS1	1	0	Inf	8.77 (1.17-65.86)
TM4SF1	1	1	5.88 (0.37-94.45)	22.36 (2.78-179.7)
TMBIM1	1	1	5.88 (0.37-94.41)	9.73 (1.28-73.74)
TMED3	1	0	Inf	6.19 (0.84-45.74)
TMEM116	1	0	Inf	12.68 (1.66-96.94)
TMEM132B	1	0	Inf	31.04 (3.61-267)
TMEM144	2	1	11.83 (1.07-131.1)	10.88 (2.59-45.69)
TMEM161A	1	0	Inf	25.67 (3.08-214.27)
TMEM256	1	0	Inf	19.85 (2.5-157.48)
TMEM5	1	0	Inf	19.12 (2.38-153.62)
TMEM70	1	0	Inf	6.16 (0.84-45.48)
TMEM86A	1	0	Inf	22.18 (2.76-178.22)
TMEM8C	1	0	Inf	44.59 (4.96-400.84)
TNFAIP6	1	1	5.88 (0.37-94.36)	5.26 (0.72-38.6)
TNFRSF18	1	0	Inf	4.9 (0.66-36.26)

TNRC6C	1	0	Inf	27.42 (3.19-235.87)
TOM1	1	0	Inf	8.54 (1.14-64.14)
TP53	1	0	Inf	65.67 (5.93-727.42)
TP53I3	1	0	Inf	5.27 (0.72-38.7)
TPP1	1	0	Inf	6.64 (0.9-49.09)
TRDMT1	1	0	Inf	16.85 (2.15-132.27)
TREML1	1	1	5.88 (0.37-94.45)	14.77 (1.91-114.17)
TRIM52	1	0	Inf	19.69 (2.48-156.24)
TRIP11	4	3	7.93 (1.76-35.7)	15.91 (5.66-44.7)
TRPC3	1	0	Inf	11.86 (1.55-90.63)
TRPM8	1	0	Inf	7.67 (1.03-57.12)
TSNAXIP1	4	1	23.92 (2.66-215.19)	12.28 (4.41-34.25)
TSSK4	1	0	Inf	5.95 (0.81-43.87)
TTC14	1	1	5.87 (0.37-94.28)	6.71 (0.9-49.75)
TTLL1	1	0	Inf	7.13 (0.96-52.92)
TTLL12	1	0	Inf	8.73 (1.17-65.39)
TULP3	2	1	11.83 (1.07-131.1)	7.35 (1.76-30.65)
TXN2	1	0	Inf	25.5 (3.12-208.29)
TXNDC12	2	0	Inf	110.6 (18.37-665.77)
UBE4B	1	0	Inf	35.42 (4.12-304.61)
UCP2	1	0	Inf	6.54 (0.88-48.44)
UFC1	1	0	Inf	44.83 (4.99-403.01)
UGDH	1	0	Inf	6.77 (0.91-50.14)
UGGT1	1	0	Inf	10.11 (1.34-76.35)
UIMC1	2	2	5.91 (0.83-42.22)	45.02 (9.5-213.44)
UNKL	1	1	5.69 (0.35-91.3)	5.93 (0.76-46.15)
UPP2	1	0	Inf	6.12 (0.83-45.19)
URB1	2	1	11.55 (1.04-128.02)	14.23 (2.36-85.66)
UROS	1	0	Inf	12.54 (1.64-95.83)
USE1	1	0	Inf	42.6 (4.74-382.97)
USP20	1	0	Inf	9.53 (1.27-71.78)
USP44	2	0	Inf	13.25 (3.13-56.12)
USP6NL	1	0	Inf	13.06 (1.68-101.71)
UTS2	1	0	Inf	8.14 (1.09-60.67)
VCL	1	1	5.88 (0.37-94.49)	16.13 (2.07-125.54)
VIM	1	0	Inf	Inf
VIPR1	2	2	5.9 (0.83-42.15)	27.5 (6.11-123.72)
VNN1	1	1	5.88 (0.37-94.45)	14.8 (1.92-114.44)
VPREB1	1	0	Inf	29.35 (3.52-244.97)
VPS16	1	0	Inf	16.23 (2.08-126.37)
VPS9D1	1	0	Inf	18.19 (2.26-146.2)
VSTM2B	1	0	Inf	9.73 (0.88-107.85)
WBSCR27	1	0	Inf	11.62 (1.52-88.82)
WDR38	3	3	5.88 (1.18-29.36)	14.59 (4.46-47.73)
XRCC2	1	0	Inf	8.43 (1.13-62.97)
XRCC6BP1	1	0	Inf	13.66 (1.78-104.93)
ZBTB9	1	0	Inf	19.57 (2.47-155.29)
ZCCHC10	1	0	Inf	58.32 (6.04-563.23)
ZCCHC7	2	0	Inf	32.61 (7.18-148.16)
ZIM2	1	0	Inf	16.29 (2.09-126.83)

ZKSCAN8	1	1	5.88 (0.37-94.45)	19.79 (2.49-156.96)
ZMYM6	1	0	Inf	7.19 (0.96-53.73)
ZMYND8	1	0	Inf	177.47 (11.06-2848.16)
ZNF146	1	0	Inf	Inf
ZNF177	1	0	Inf	7.55 (1.01-56.57)
ZNF234	1	1	5.88 (0.37-94.49)	5.58 (0.76-41.02)
ZNF256	1	0	Inf	4.96 (0.68-36.4)
ZNF300	1	0	Inf	10.65 (1.4-80.72)
ZNF354A	1	1	5.88 (0.37-94.45)	25.44 (3.11-207.77)
ZNF391	1	1	5.88 (0.37-94.45)	9.94 (1.32-74.88)
ZNF484	1	0	Inf	11.9 (1.56-90.57)
ZNF500	1	0	Inf	21.54 (2.68-173.05)
ZNF567	1	0	Inf	17.62 (2.24-138.34)
ZNF607	2	2	5.91 (0.83-42.2)	6.42 (1.56-26.5)
ZNF716	2	0	Inf	12.12 (2.86-51.35)
