# Supplementary material for: Whole exome sequencing implicates eye development, the unfolded protein response and plasma membrane homeostasis in primary open-angle glaucoma
Source: PLoS One. 2017 Mar 6;12(3):e0172427. doi: 10.1371/journal.pone.0172427 (PMC5338784; doi:10.1371/journal.pone.0172427)
Supplement: S3 Table — (PDF) [file pone.0172427.s005.pdf]

S3 Table: List of enriched genes for high-tension glaucoma cohort under a predicted pathogenic model

Headings:
Gene: HGNC gene name
HTG: Number of cases in high-tension glaucoma cohort
CTRL: Number of cases in local and AOGC controls
HTG CTRL OR (95%CI): Odds ratio of high-tension glaucoma cohort compared to controls
HTG NFE OR (95% CI): Odds ratio of high-tension glaucoma cohort compared to non-Finnish European ExAC public domain data

Gene	HTG	CTRL	HTG CTRL OR (95% CI)	HTG NFE OR (95% CI)
ACD	5	2	20.7 (3.97-107.89)	6.22 (2.52-15.38)
ACP1	1	2	4.52 (0.41-50.18)	9.01 (1.22-66.61)
ACTA1	1	0	Inf	12.29 (1.64-91.93)
ADPRHL2	2	3	6.06 (1-36.64)	4.36 (1.07-17.85)
AES	1	0	Inf	12.63 (1.65-96.78)
AGO1	1	1	9.03 (0.56-145.34)	7.79 (1.06-57.36)
AGPAT3	1	1	8.52 (0.53-137.17)	7.7 (1.05-56.66)
AGPAT6	1	1	9.04 (0.56-145.48)	6.38 (0.87-46.69)
AKTIP	1	1	9.04 (0.56-145.48)	7.21 (0.98-52.97)
ALDOC	3	4	6.87 (1.52-31.06)	8.53 (2.66-27.27)
AMMECR1L	1	0	Inf	17.09 (2.25-129.88)
ANAPC10	1	0	Inf	21.18 (2.75-163.19)
ANKRD13C	1	2	4.35 (0.39-48.37)	6.14 (0.84-44.9)
AP1M1	1	0	Inf	4.75 (0.65-34.67)
APOM	1	0	Inf	9.85 (1.33-73.08)
ARIH1	1	1	5.69 (0.35-91.52)	18.13 (2.32-141.52)
ARL5B	1	1	9.04 (0.56-145.48)	10.14 (1.37-75.2)
ARL6IP5	1	0	Inf	5.94 (0.81-43.45)
ARMC1	2	4	4.55 (0.82-25.08)	9.16 (2.21-37.9)
ARMC8	1	1	9.04 (0.56-145.48)	4.85 (0.67-35.39)
ASB8	2	3	6.07 (1-36.67)	6.68 (1.62-27.46)
ATAD2	2	3	5.85 (0.97-35.38)	6.17 (1.5-25.36)
ATF2	1	1	9.04 (0.56-145.41)	7.7 (1.05-56.68)
ATG3	2	0	Inf	11.72 (2.82-48.8)
ATP5I	1	1	8.11 (0.5-130.46)	6.82 (0.93-50.12)
ATP6V1G1	1	1	9.03 (0.56-145.34)	8.05 (1.07-60.59)
ATXN10	1	0	Inf	5.43 (0.74-39.69)
AVPR1A	2	3	5.92 (0.98-35.79)	5.96 (1.45-24.49)
B3GNT7	1	0	Inf	7.14 (0.97-52.52)
BAD	2	1	14.03 (1.26-155.95)	4.23 (1.03-17.31)
BPIFA1	1	0	Inf	6.06 (0.83-44.35)
BTBD3	1	1	7.86 (0.49-126.47)	6.35 (0.87-46.5)
BZW2	1	0	Inf	9.69 (1.31-71.89)
C10orf95	1	0	Inf	19.31 (2.47-150.71)
C11orf86	1	1	8.4 (0.52-135.11)	27.65 (1.72-444.64)
C15orf48	1	1	9.03 (0.56-145.34)	19.18 (2.5-147.04)
C1orf226	1	1	8.98 (0.56-144.48)	6.2 (0.84-45.83)
C1orf52	1	1	7.91 (0.49-127.27)	10.24 (1.37-76.28)
C4A	2	0	Inf	38.81 (8.73-172.64)
C4orf32	1	0	Inf	6.85 (0.93-50.67)
C6orf120	2	0	Inf	10.34 (2.48-43.12)
C6orf52	1	0	Inf	12.13 (1.25-117.42)
C7orf25	1	1	6.33 (0.39-101.93)	5.84 (0.8-42.75)
C8orf37	1	0	Inf	7.85 (1.07-57.73)
C8orf59	1	1	9.04 (0.56-145.41)	5.8 (0.79-42.56)
CACNG4	1	1	8.83 (0.55-142.09)	8.57 (1.16-63.35)
CALU	1	1	9.04 (0.56-145.48)	7.81 (1.06-57.62)
CAMK1	3	2	13.77 (2.28-83.27)	6.53 (2.05-20.79)
CBFB	1	0	Inf	14.5 (1.93-109.21)

CBLL1	1	1	9.04 (0.56-145.48)	8.31 (1.13-61.27)
CCL7	1	2	4.51 (0.41-50.13)	8.87 (1.2-65.48)
CCRN4L	1	0	Inf	8.6 (1.17-63.41)
CDC23	1	1	9.04 (0.56-145.48)	10.94 (1.47-81.36)
CDC42EP2	1	2	4.48 (0.4-49.83)	9.63 (1.3-71.36)
CDKN2AIP	2	1	18.23 (1.64-202.58)	9.71 (2.34-40.28)
CDYL	1	1	9.04 (0.56-145.48)	6.05 (0.83-44.3)
CEACAM18	2	0	Inf	5.54 (1.35-22.73)
CEBPD	1	0	Inf	13.78 (1.81-105.11)
CELF2	1	2	4.51 (0.41-50.11)	8.53 (1.16-62.9)
CEP76	2	4	4.48 (0.81-24.73)	10.6 (2.55-44.02)
CHAMP1	2	2	9.11 (1.27-65.25)	21.32 (5-90.83)
CHMP1B	1	0	Inf	9.06 (1.22-67.44)
CHP1	1	0	Inf	30.56 (3.84-243.09)
CHRAC1	1	0	Inf	25.65 (3.22-204.01)
CHST2	2	1	13.18 (1.19-146.51)	16.19 (3.81-68.83)
CIART	2	3	6.06 (1-36.64)	4.58 (1.12-18.74)
CLCN3	2	1	18.23 (1.64-202.58)	7 (1.7-28.82)
CLEC4D	2	2	9.11 (1.27-65.23)	14.63 (3.49-61.37)
CLVS1	1	0	Inf	11.9 (1.59-88.79)
CNNM4	2	4	4.47 (0.81-24.64)	4.09 (1-16.72)
CNOT3	2	0	Inf	4.65 (1.14-19.06)
COQ10B	1	0	Inf	10.43 (1.4-77.51)
COX19	1	0	Inf	5.23 (0.72-38.2)
COX5A	1	0	Inf	4.3 (0.59-31.24)
COX6A1	1	1	8.17 (0.51-131.39)	7.99 (1.08-58.8)
CPLX2	1	1	8.17 (0.51-131.52)	11.19 (1.49-84.25)
CPOX	2	1	7.21 (0.65-80.21)	7.45 (1.81-30.71)
CST7	2	2	8.53 (1.19-61.09)	16.8 (3.99-70.81)
CTDSP1	1	0	Inf	9.9 (1.33-73.68)
CYP46A1	1	1	9.04 (0.56-145.48)	9.73 (1.31-72.54)
DCAF5	3	2	13.78 (2.28-83.28)	7.01 (2.2-22.38)
DEFB135	2	2	9.11 (1.27-65.25)	8.62 (2.09-35.62)
DEFB136	1	0	Inf	4.06 (0.56-29.5)
DESI2	1	0	Inf	29.4 (3.7-233.88)
DGCR6L	2	3	4.21 (0.7-25.46)	5.27 (1.28-21.66)
DHX40	1	2	4.52 (0.41-50.18)	5.45 (0.75-39.77)
DIRC2	1	1	9.04 (0.56-145.48)	4.64 (0.64-33.8)
DNAJC11	2	1	18.22 (1.64-202.44)	7.32 (1.78-30.16)
DPF2	1	1	9.04 (0.56-145.48)	8.69 (1.18-64.19)
DRGX	1	1	8.86 (0.55-142.62)	5.45 (0.74-39.91)
DUSP19	1	2	4.51 (0.41-50.12)	6.85 (0.93-50.2)
DYDC1	2	1	18.23 (1.64-202.58)	22.03 (5.16-94.06)
EBF3	1	2	4.5 (0.41-49.99)	5.45 (0.75-39.82)
EEF1E1	1	0	Inf	14.82 (1.96-112.22)
EFCAB1	1	1	9.04 (0.56-145.41)	5.48 (0.75-39.98)
EIF5A	1	2	4.52 (0.41-50.18)	13.24 (1.72-101.98)
ENTPD8	3	6	4.49 (1.11-18.2)	4.46 (1.4-14.16)
ERICH2	3	0	Inf	21.39 (4.27-107.12)
ERLIN2	2	0	Inf	19.8 (4.67-84.07)

EVA1B	1	0	Inf	4.89 (0.66-36.02)
FAM110A	2	0	Inf	8.2 (1.97-34.14)
FAM181B	1	0	Inf	8.03 (1.06-60.84)
FAM89B	1	0	Inf	13.9 (1.85-104.65)
FBXW12	3	3	9.17 (1.83-45.97)	7.32 (2.29-23.36)
FGF19	1	1	4.48 (0.28-72.12)	37.19 (4.54-304.61)
FGF9	1	0	Inf	25 (3.2-195.17)
FGL2	3	4	6.87 (1.52-31.05)	8.96 (2.8-28.67)
FKBP3	1	2	4.27 (0.38-47.49)	16.94 (2.23-128.76)
FLVCR1	2	3	5.99 (0.99-36.21)	9.29 (2.24-38.49)
FLVCR2	1	1	8.9 (0.55-143.22)	3.99 (0.55-28.94)
FOXD2	1	0	Inf	11.2 (1.47-85.15)
FRAT1	1	1	8.65 (0.54-139.23)	10.15 (1.31-78.68)
FXYD4	1	0	Inf	8.34 (1.13-61.5)
FXYD7	1	0	Inf	39.35 (4.8-322.26)
GAS1	2	0	Inf	23.14 (5.02-106.73)
GATA3	2	2	6.18 (0.86-44.28)	13.5 (3.22-56.56)
GATAD1	1	0	Inf	8.09 (1.1-59.72)
GCHFR	1	1	9.02 (0.56-145.08)	6.02 (0.82-44.26)
GDF1	2	0	Inf	7.6 (1.67-34.65)
GEMIN2	1	0	Inf	7.78 (1.06-57.24)
GFPT1	2	1	18.23 (1.64-202.58)	15.03 (3.58-63.13)
GGACT	1	0	Inf	19.17 (1.19-308.35)
GLTP	1	0	Inf	4.75 (0.65-34.6)
GLYR1	1	0	Inf	4.85 (0.67-35.36)
GPN2	2	1	17.83 (1.6-198.05)	7.29 (1.77-30.05)
GPR183	1	0	Inf	18.28 (2.4-139.46)
GPR20	3	3	8.54 (1.7-42.78)	5.51 (1.72-17.59)
GSPT1	1	1	4.94 (0.31-79.56)	8.28 (1.11-61.79)
GTF2B	1	1	9.03 (0.56-145.34)	7.23 (0.98-53.06)
H2AFZ	1	1	9.02 (0.56-145.08)	274.99 (17.1-4421.97)
HAND2	1	0	Inf	46.68 (5.41-402.5)
HAUS2	1	1	9.03 (0.56-145.28)	8.3 (1.13-61.24)
HBEGF	1	0	Inf	22.99 (2.95-179.47)
HDGFL1	1	0	Inf	6.5 (0.88-48.13)
HECA	1	2	4.52 (0.41-50.19)	5.77 (0.79-42.22)
HES5	1	0	Inf	19.04 (2.42-149.93)
HIGD1B	1	0	Inf	10.89 (1.46-81.04)
HIST1H2AG	1	0	Inf	7 (0.95-51.34)
HIST1H3F	1	0	Inf	11.47 (1.54-85.48)
HIST1H4I	1	1	8.69 (0.54-139.76)	6.65 (0.91-48.71)
HIST2H2AB	1	0	Inf	11.3 (1.52-84.19)
HNRNPA3	1	0	Inf	38.41 (4.59-321.48)
HPGDS	2	1	18.23 (1.64-202.49)	6.92 (1.68-28.48)
HS3ST3B1	1	0	Inf	9.21 (1.23-68.88)
HSDL1	1	0	Inf	5.85 (0.8-42.75)
HTR1A	1	1	9.01 (0.56-144.95)	9.43 (1.27-69.81)
IDH3A	1	1	9.03 (0.56-145.34)	4.91 (0.67-35.75)
IL1RN	1	1	9.04 (0.56-145.41)	5.17 (0.71-37.72)
IL5	1	0	Inf	10.58 (1.42-78.6)

IMPAD1	1	1	9.05 (0.56-145.54)	8.58 (1.16-63.46)
INSL3	2	0	Inf	9.01 (2.15-37.73)
INSM2	2	0	Inf	5.57 (1.36-22.86)
IPO13	1	1	9.03 (0.56-145.34)	4.69 (0.64-34.13)
IRF2BP2	1	0	Inf	5.63 (0.77-41.21)
JPH4	2	3	5.94 (0.98-35.93)	5.25 (1.28-21.57)
JUNB	1	0	Inf	10.13 (1.35-76.07)
KCTD4	1	0	Inf	13.1 (1.75-98.17)
KLF13	1	0	Inf	12.77 (1.69-96.72)
KRTAP29-1	2	0	Inf	11.16 (2.02-61.55)
LBX1	1	1	8.89 (0.55-143.08)	6.56 (0.89-48.14)
LCE6A	1	2	4.51 (0.41-50.11)	5.62 (0.67-47.07)
LCN6	2	1	18.16 (1.63-201.75)	8.24 (1.99-34.09)
LEMD1	1	0	Inf	8.14 (1.1-59.98)
LHX9	1	0	Inf	7.4 (1.01-54.34)
LMO2	1	0	Inf	10.27 (1.38-76.51)
LMO7DN	1	0	Inf	Inf
LOC730159	1	0	Inf	14.48 (0.9-233.01)
LPCAT4	3	4	6.87 (1.52-31.06)	9.97 (3.1-32.01)
LRP2BP	3	6	4.57 (1.13-18.53)	5.93 (1.86-18.88)
LYPD2	2	2	8.95 (1.25-64.14)	6.63 (1.6-27.44)
LYPD8	1	2	4.51 (0.41-50.13)	7.02 (0.81-60.51)
MAB21L2	1	1	8.99 (0.56-144.61)	9.38 (1.27-69.4)
MBNL2	1	2	4.5 (0.4-49.97)	7.21 (0.98-52.92)
MBTD1	1	1	9.05 (0.56-145.54)	12.51 (1.67-93.75)
METTL7A	2	1	18.21 (1.64-202.3)	5.31 (1.3-21.78)
MGAT2	1	0	Inf	4.43 (0.61-32.2)
MLLT3	1	0	Inf	7.6 (1.03-55.9)
MMP16	1	0	Inf	5.71 (0.78-41.74)
MOB3C	2	1	16.86 (1.52-187.32)	4.5 (1.1-18.41)
MRPL12	1	0	Inf	6.66 (0.91-48.92)
MRPL17	2	2	9.04 (1.26-64.75)	17.44 (4.13-73.7)
MRPL33	1	2	4.52 (0.41-50.18)	6.07 (0.83-44.39)
MRPS10	3	1	27.58 (2.85-267.25)	10.16 (3.16-32.63)
MRPS12	1	0	Inf	11.61 (1.56-86.66)
MS4A6E	1	1	9.05 (0.56-145.54)	15.31 (2.03-115.59)
MTA2	1	2	4.52 (0.41-50.18)	4.25 (0.58-30.88)
MYOT	3	5	5.49 (1.3-23.26)	7.42 (2.32-23.69)
N6AMT2	1	1	9.04 (0.56-145.48)	4.59 (0.63-33.42)
NACC1	2	1	15.58 (1.4-173.07)	18.97 (4.46-80.68)
NAPB	1	0	Inf	9.78 (1.32-72.54)
NAT14	1	1	7.05 (0.44-113.45)	16.77 (1.94-144.63)
NBL1	1	0	Inf	7.35 (0.76-71.22)
NDFIP2	1	1	8.45 (0.53-135.98)	16.25 (2.14-123.54)
NDRG3	1	1	9.04 (0.56-145.48)	7.05 (0.96-51.76)
NDUFA12	2	1	18.23 (1.64-202.52)	13.53 (3.23-56.56)
NFE2L2	2	2	9.11 (1.27-65.27)	5.94 (1.45-24.39)
NFKBIL1	2	0	Inf	10.63 (2.55-44.37)
NGB	3	0	Inf	33.71 (9.69-117.22)
NKX2-5	1	0	Inf	9.96 (1.33-74.83)

NKX6-2	1	0	Inf	5.58 (0.76-40.85)
NMI	2	1	18.23 (1.64-202.58)	4.84 (1.18-19.82)
NOL7	2	2	8.12 (1.13-58.2)	13.43 (3.19-56.59)
NOXRED1	2	0	Inf	8.06 (1.95-33.26)
NPW	1	0	Inf	6.32 (0.85-47.04)
NR2E1	1	0	Inf	18.52 (2.4-142.66)
NRGN	1	2	4.05 (0.36-44.95)	63.42 (6.55-614.03)
NRL	1	0	Inf	10.48 (1.4-78.55)
NUP54	2	1	18.23 (1.64-202.58)	16.53 (3.92-69.76)
NUSAP1	4	3	12.33 (2.73-55.74)	7.68 (2.79-21.15)
OTX1	1	0	Inf	7.98 (1.08-58.74)
OTX2	1	0	Inf	10.53 (1.42-78.23)
PAQR8	1	2	4.52 (0.41-50.18)	8.04 (1.09-59.18)
PBK	2	2	9.1 (1.27-65.21)	7.21 (1.75-29.69)
PDCD7	2	1	9.14 (0.82-101.64)	7.36 (1.78-30.38)
PDHB	1	1	9.04 (0.56-145.48)	7.16 (0.98-52.6)
PEBP1	1	0	Inf	4.21 (0.58-30.6)
PELO	1	1	9.02 (0.56-145.21)	15.16 (2.01-114.45)
PEX11B	2	1	16.21 (1.46-180.14)	5.53 (1.35-22.71)
PEX3	1	0	Inf	9.74 (1.31-72.16)
PF4	2	2	9.11 (1.27-65.25)	39.76 (8.88-178.1)
PGF	3	4	6.79 (1.5-30.69)	21.39 (6.51-70.35)
PIGC	1	0	Inf	6.09 (0.83-44.56)
PLSCR2	2	2	6.4 (0.89-45.85)	5.07 (1.24-20.76)
PNPLA8	3	4	6.87 (1.52-31.08)	9.39 (2.93-30.09)
POPDC2	3	5	5.49 (1.3-23.27)	5.1 (1.6-16.2)
PPARD	1	1	9.03 (0.56-145.34)	6.08 (0.83-44.48)
PPBP	1	2	4.52 (0.41-50.18)	13.12 (1.75-98.32)
PPIH	2	2	9.1 (1.27-65.19)	13.83 (3.31-57.88)
PPM1L	1	1	9.04 (0.56-145.48)	8.06 (1.1-59.39)
PPP2R5A	1	1	9.03 (0.56-145.34)	6.23 (0.85-45.65)
PPP3CA	1	2	4.47 (0.4-49.63)	7.95 (1.08-58.57)
PRIMA1	1	0	Inf	5.67 (0.77-41.59)
PRPSAP2	1	0	Inf	7.15 (0.97-52.47)
PSEN1	1	2	4.52 (0.41-50.18)	10.93 (1.47-81.29)
PSMB1	1	1	9.04 (0.56-145.48)	9.45 (1.28-70.05)
PSMD11	1	1	9.03 (0.56-145.34)	5.5 (0.75-40.15)
PTCHD4	4	3	12.34 (2.73-55.8)	5.64 (2.06-15.42)
RAB13	1	1	9.03 (0.56-145.34)	8.54 (1.16-63)
RAB15	1	1	6.37 (0.4-102.55)	4.1 (0.56-29.78)
RAB27A	2	2	9.1 (1.27-65.19)	4.71 (1.15-19.28)
RAB6A	1	0	Inf	16.91 (2.23-128.55)
RAD21L1	3	0	Inf	36.73 (7.34-183.86)
RARRES2	1	0	Inf	9.28 (1.24-69.57)
RCBTB1	2	4	4.54 (0.82-25.07)	4.6 (1.12-18.84)
RCN2	1	0	Inf	14.08 (1.86-106.31)
REEP3	1	1	9.04 (0.56-145.41)	14.05 (1.84-107.22)
RGS17	1	0	Inf	7.95 (1.08-58.58)
RGS2	2	2	9.1 (1.27-65.19)	10.25 (2.47-42.52)
RIC8B	1	1	9.04 (0.56-145.48)	5.84 (0.8-42.67)

RIPPLY2	1	0	Inf	4.51 (0.62-32.83)
RLBP1	2	3	5.98 (0.99-36.14)	5.88 (1.43-24.14)
RNF141	3	4	6.88 (1.52-31.09)	44.15 (12.89-151.17)
RNF144A	1	2	4.52 (0.41-50.18)	4.1 (0.56-29.76)
RNF166	1	0	Inf	5.7 (0.77-41.94)
RPL15	1	0	Inf	16.94 (2.17-132.26)
RPL18A	1	0	Inf	6.48 (0.88-47.46)
RPL39L	1	0	Inf	5.16 (0.71-37.6)
RPL5	1	1	9.03 (0.56-145.34)	8.02 (1.09-59.09)
RPP38	1	1	9.04 (0.56-145.48)	6.5 (0.89-47.59)
RPRM	1	1	8.86 (0.55-142.55)	18.79 (2.45-144.04)
RRAGA	1	0	Inf	22.95 (2.96-177.88)
RSPO3	1	1	9.04 (0.56-145.48)	8.7 (1.18-64.25)
RUVBL2	3	5	5.39 (1.27-22.86)	5.66 (1.78-18.01)
RXRA	2	0	Inf	18.73 (4.42-79.38)
S100A2	1	1	9.03 (0.56-145.34)	5.72 (0.78-41.78)
SERPINF2	1	0	Inf	8.11 (1.1-59.79)
SGCZ	1	1	9.04 (0.56-145.48)	6.81 (0.93-49.99)
SGMS2	1	0	Inf	5.08 (0.7-37)
SIK2	2	3	6.07 (1-36.67)	4.68 (1.14-19.14)
SIN3A	2	3	6.06 (1-36.62)	8.52 (2.06-35.21)
SIRT6	2	3	5.68 (0.94-34.35)	6.57 (1.58-27.3)
SIRT7	1	0	Inf	6.86 (0.93-50.4)
SLBP	2	0	Inf	23.11 (5.4-98.89)
SLC25A25	2	2	9.02 (1.26-64.61)	7.54 (1.83-31.09)
SLC35F6	2	0	Inf	11.44 (2.75-47.71)
SLC48A1	1	0	Inf	8.17 (1.1-60.36)
SLC6A3	2	2	9.05 (1.26-64.86)	4.74 (1.16-19.43)
SLX4IP	2	1	18.23 (1.64-202.58)	13.13 (3.14-54.84)
SMIM14	2	1	18.23 (1.64-202.58)	24.1 (5.62-103.37)
SMIM18	1	0	Inf	Inf
SMPD2	2	3	6.01 (1-36.36)	5.88 (1.43-24.14)
SMR3A	1	1	9.04 (0.56-145.48)	9.16 (1.24-67.73)
SNAP25	1	0	Inf	25.57 (3.25-201.3)
SNN	1	0	Inf	15.98 (2.11-121.03)
SNX27	2	1	18.22 (1.64-202.4)	8.94 (2.15-37.12)
SNX33	1	2	4.5 (0.41-50.04)	4.23 (0.58-30.74)
SNX7	1	0	Inf	4.05 (0.56-29.44)
SPINK1	1	0	Inf	13.18 (1.75-98.99)
SPINK4	1	1	9.04 (0.56-145.48)	16.69 (2.2-126.86)
SPTY2D1	2	3	6.06 (1-36.64)	8.38 (2.03-34.58)
SRF	1	1	4.94 (0.31-79.56)	4.05 (0.56-29.44)
SRR	1	0	Inf	7.24 (0.99-53.15)
SRSF11	1	0	Inf	6.23 (0.85-45.58)
STAG1	1	0	Inf	6.63 (0.9-48.59)
STK24	1	0	Inf	8.05 (1.09-59.29)
STK4	2	1	18.23 (1.64-202.58)	12.02 (2.89-50.08)
SUPT16H	1	0	Inf	7.05 (0.96-51.7)
SVIP	1	0	Inf	7.6 (1.03-56.11)
SYT9	2	3	6.07 (1-36.67)	6.85 (1.67-28.21)

TAC3	1	0	Inf	11.24 (1.51-83.77)
TBC1D22A	3	4	6.44 (1.42-29.13)	7.52 (2.35-24.06)
TCTN3	4	4	9.19 (2.27-37.25)	12.19 (4.4-33.78)
TFAP2C	1	0	Inf	5.67 (0.78-41.44)
TGFB1	2	2	9.1 (1.27-65.16)	5.42 (1.32-22.29)
TLX1	1	0	Inf	6.16 (0.83-45.42)
TMBIM1	2	2	9.11 (1.27-65.24)	5.57 (1.36-22.88)
TMEM106A	2	4	4.55 (0.82-25.09)	7.5 (1.82-30.88)
TMEM116	2	1	18.22 (1.64-202.46)	9.06 (2.19-37.51)
TMEM14E	1	0	Inf	8.25 (1.12-60.78)
TMEM63B	1	0	Inf	4.02 (0.55-29.21)
TMEM65	1	1	9.04 (0.56-145.48)	23.4 (2.97-184.19)
TMEM70	1	2	4.52 (0.41-50.21)	4.46 (0.61-32.4)
TMEM8B	5	5	8.9 (2.54-31.2)	10.38 (4.17-25.83)
TMEM92	1	0	Inf	11.28 (1.51-84.03)
TNFSF14	1	1	9.04 (0.56-145.41)	9.7 (1.31-72.08)
TNFSF18	2	2	9.1 (1.27-65.18)	9.33 (2.25-38.66)
TOX	1	2	4.52 (0.41-50.18)	14.41 (1.91-108.48)
TSLP	2	3	6.06 (1-36.65)	9.74 (2.35-40.33)
TSPAN18	2	0	Inf	9.21 (2.23-38.12)
TSPAN3	1	1	9.03 (0.56-145.34)	13.49 (1.79-101.86)
TTC9	1	2	4.52 (0.41-50.18)	15.56 (2.05-118.26)
UBA3	1	1	9.04 (0.56-145.48)	5.99 (0.82-43.79)
UBIAD1	1	0	Inf	14.49 (1.92-109.1)
UBP1	2	2	9.11 (1.27-65.25)	5.87 (1.43-24.1)
UBXN7	1	1	9.04 (0.56-145.48)	11.89 (1.59-88.73)
UCHL5	2	0	Inf	28.79 (6.58-125.97)
UCK1	1	2	4.49 (0.4-49.93)	5.05 (0.69-36.83)
UNC5D	3	2	9.74 (1.61-58.92)	5.04 (1.59-16.02)
USP30	1	0	Inf	4.45 (0.61-32.4)
VDAC3	1	1	9.04 (0.56-145.48)	6.83 (0.93-50.08)
WBSCR16	1	2	4.52 (0.41-50.18)	9.14 (1.22-68.46)
WDR45B	1	2	4.52 (0.41-50.18)	6.44 (0.88-47.14)
WDR61	1	1	9.03 (0.56-145.34)	5.48 (0.75-40.01)
WIPI2	1	1	9.04 (0.56-145.48)	5.85 (0.8-42.78)
YRDC	1	0	Inf	10.47 (1.4-78.46)
YWHAB	1	0	Inf	6.22 (0.85-45.52)
YWHAE	1	1	8.43 (0.52-135.64)	27.37 (3.48-215.45)
ZBTB34	1	0	Inf	11.8 (1.58-88.09)
ZBTB44	3	2	13.77 (2.28-83.26)	7.05 (2.21-22.51)
ZCCHC9	1	2	4.52 (0.41-50.18)	5.08 (0.7-36.99)
ZDHHC17	1	2	4.43 (0.4-49.19)	5 (0.68-36.67)
ZIC2	1	0	Inf	10.36 (1.38-77.67)
ZMYND19	1	0	Inf	8.21 (1.11-60.52)
ZNF131	1	1	9.04 (0.56-145.48)	5.22 (0.72-38.06)
ZNF146	2	1	18.25 (1.64-202.77)	24.11 (5.62-103.39)
ZNF605	1	2	4.52 (0.41-50.18)	13.72 (1.83-103.05)
ZNF716	6	0	Inf	7.86 (3.42-18.07)
ZNF768	2	1	17.54 (1.58-194.9)	11.53 (2.77-47.99)
ZPBP2	2	1	18.22 (1.64-202.46)	8.35 (2.02-34.46)
