# Supplementary material for: Whole exome sequencing implicates eye development, the unfolded protein response and plasma membrane homeostasis in primary open-angle glaucoma
Source: PLoS One. 2017 Mar 6;12(3):e0172427. doi: 10.1371/journal.pone.0172427 (PMC5338784; doi:10.1371/journal.pone.0172427)
Supplement: S4 Table — (PDF) [file pone.0172427.s006.pdf]

S4 Table: List of enriched genes for high-tension glaucoma cohort under a loss of function model

Headings:
Gene: HGNC gene name
HTG: Number of cases in high-tension glaucoma cohort
CTRL: Number of cases in local and AOGC controls
HTG CTRL OR (95% CI): Odds ratio of high-tension glaucoma cohort compared to controls
HTG NFE OR (95% CI): Odds ratio of high-tension glaucoma cohort compared to non-Finnish European ExAC public domain data

Gene	HTG	CTRL	HTG CTRL OR (95% CI)	HTG NFE OR (95% CI)
ABCA2	1	0	Inf	18.99 (2.41-149.52)
ABCC3	1	0	Inf	7.81 (1.06-57.5)
ACADVL	2	0	Inf	30.93 (7.07-135.34)
ACD	1	0	Inf	10.05 (1.35-74.63)
ACOX1	1	0	Inf	45.87 (5.48-383.9)
ADAM9	2	0	Inf	111.06 (21.34-578.07)
ADPRHL1	1	1	9.03 (0.56-145.28)	7.98 (1.08-58.79)
ADTRP	1	0	Inf	21.71 (2.73-172.67)
AFAP1L2	1	0	Inf	13.06 (1.72-98.88)
AGPAT2	1	0	Inf	25.72 (3.23-204.58)
AHSG	1	0	Inf	17 (2.24-129.24)
AKAP12	1	1	9.04 (0.56-145.48)	24.28 (3.11-189.55)
ALB	1	0	Inf	24.82 (3.18-193.71)
ANKDD1A	1	0	Inf	10.96 (1.47-81.64)
ANKRD27	1	1	9.04 (0.56-145.48)	5.74 (0.79-41.99)
ANO3	1	1	9.04 (0.56-145.48)	15.04 (1.99-113.58)
AP3B1	1	0	Inf	131.14 (11.81-1455.94)
APMAP	1	1	9.04 (0.56-145.48)	25.98 (3.3-204.53)
APOBR	1	1	8.99 (0.56-144.68)	7.71 (1.04-57.32)
ARMC8	1	0	Inf	122.85 (11.07-1363.87)
ARRDC2	1	0	Inf	15.51 (2.04-117.85)
ARSK	1	0	Inf	28.8 (3.62-229.07)
ASB8	1	0	Inf	5.73 (0.78-41.86)
ATAD3B	1	0	Inf	7.97 (1.08-58.71)
ATG3	1	0	Inf	68.15 (7.56-614.24)
ATG4D	1	0	Inf	10.8 (1.44-80.8)
ATG7	1	0	Inf	11.89 (1.59-88.76)
ATP6V0A2	1	0	Inf	18.08 (2.37-137.93)
ATP6V1E2	1	0	Inf	21.15 (2.74-162.92)
AVPR1A	1	1	8.71 (0.54-140.23)	38.65 (4.72-316.51)
BAAT	1	0	Inf	30.42 (3.82-241.98)
BCAT1	1	0	Inf	28.87 (3.63-229.64)
BDH2	1	1	9.04 (0.56-145.48)	9.87 (1.33-73.33)
BIN3	1	0	Inf	27.45 (3.45-218.36)
BNIP1	1	1	9.05 (0.56-145.54)	5.17 (0.71-37.68)
BRD9	1	0	Inf	54.89 (6.37-473.33)
BSCL2	1	1	9.05 (0.56-145.54)	15.83 (2.09-119.9)
C11orf53	1	0	Inf	19.58 (2.55-150.06)
C12orf56	1	0	Inf	7.09 (0.96-52.61)
C14orf177	1	1	9.03 (0.56-145.34)	24.11 (3.09-188.23)
C1orf109	1	0	Inf	23.74 (3.04-185.33)
C3orf30	1	1	9.04 (0.56-145.41)	16.09 (2.12-121.84)
C7orf31	2	1	18.23 (1.64-202.52)	14.53 (3.46-60.97)
CABS1	1	0	Inf	19.51 (2.54-149.5)
CACNA1S	1	0	Inf	8.4 (1.14-61.95)
CAPN13	1	1	9.05 (0.56-145.54)	5.68 (0.77-41.6)
CAPS2	1	0	Inf	6.14 (0.84-45.09)
CATSPER2	1	0	Inf	13.01 (1.74-97.51)
CATSPERG	3	1	27.53 (2.84-266.76)	8.78 (2.74-28.18)

CAV1	1	0	Inf	30.58 (3.84-243.24)
CBX2	1	0	Inf	24.38 (3.12-190.32)
CCBE1	1	0	Inf	15.23 (2.02-115.01)
CCDC57	2	1	18.13 (1.63-201.47)	13.9 (3.3-58.43)
CCR5	3	3	9.18 (1.83-45.98)	10.74 (3.34-34.53)
CCT8L2	1	0	Inf	19.67 (2.57-150.77)
CD200R1L	1	0	Inf	33.7 (4.18-271.49)
CD83	1	0	Inf	83.03 (8.58-803.85)
CDC42EP2	1	0	Inf	Inf
CDK15	1	0	Inf	10.5 (1.41-77.96)
CDK5RAP2	1	0	Inf	5.81 (0.79-42.44)
CDKN2AIP	1	0	Inf	Inf
CEACAM18	1	0	Inf	16.94 (2.23-128.71)
CEMP1	1	0	Inf	90.45 (9.34-875.76)
CEP70	1	0	Inf	7.26 (0.99-53.33)
CES3	1	0	Inf	20.82 (2.7-160.43)
CHAC2	1	1	9.04 (0.56-145.41)	8.34 (1.13-61.55)
CHRNB2	1	0	Inf	64.47 (7.15-581.05)
CIB1	1	0	Inf	39.31 (4.8-321.93)
CLEC4D	2	0	Inf	44.44 (9.84-200.68)
CLPSL2	1	0	Inf	74.48 (6.71-826.91)
CLUL1	1	1	9.04 (0.56-145.52)	6.88 (0.94-50.5)
CNBD1	1	1	9.03 (0.56-145.34)	13.5 (1.78-102.24)
CNDP2	1	0	Inf	30.47 (3.83-242.35)
COL22A1	2	3	6.05 (1-36.56)	7.98 (1.93-32.98)
CPD	1	0	Inf	30.59 (3.85-243.36)
CPVL	1	1	9.04 (0.56-145.48)	7.39 (1.01-54.28)
CRYBB1	1	0	Inf	45.83 (5.48-383.58)
CTSE	1	1	9.03 (0.56-145.34)	13.73 (1.83-103.15)
CTSH	1	0	Inf	10.88 (1.46-81.05)
CTSZ	1	0	Inf	11.89 (1.59-88.92)
CUX1	1	0	Inf	20.7 (2.69-159.47)
CYP21A2	1	0	Inf	56.41 (5.83-546.12)
CYP46A1	1	0	Inf	137.81 (12.41-1530)
DCLRE1C	2	1	18.24 (1.64-202.63)	11.67 (2.8-48.58)
DEDD2	1	0	Inf	7.53 (1-56.82)
DEFB135	1	0	Inf	13.65 (1.82-102.5)
DENND6B	1	1	9 (0.56-144.75)	57.37 (6.37-517.01)
DEPTOR	1	0	Inf	30.28 (3.81-240.89)
DERL3	1	0	Inf	13.71 (1.8-104.62)
DNAJC27	1	0	Inf	54.24 (6.29-467.7)
DNASE1L3	1	0	Inf	24.9 (3.19-194.4)
DNASE2B	1	0	Inf	9.44 (1.27-70.03)
DPP6	1	1	9.05 (0.56-145.54)	36.36 (4.34-304.27)
DSN1	1	0	Inf	8.49 (1.15-62.66)
DSP	1	0	Inf	14.45 (1.92-108.81)
DUSP16	1	0	Inf	68.72 (7.63-619.34)
ECHDC1	1	1	9.04 (0.56-145.48)	9.16 (1.24-67.7)
ECI1	1	0	Inf	13.02 (1.73-98.06)
EPG5	1	0	Inf	9.42 (1.27-69.69)

ERAP1	1	1	9.04 (0.56-145.48)	5.24 (0.72-38.24)
ERLIN2	1	0	Inf	137.43 (12.38-1525.71)
EXOC4	1	1	9.04 (0.56-145.48)	18.21 (2.39-138.95)
EXOG	1	0	Inf	11.92 (1.6-89)
EXOSC3	1	0	Inf	130.38 (11.74-1447.52)
FAM120B	1	0	Inf	6.61 (0.9-48.55)
FAM186B	1	1	9.03 (0.56-145.34)	8.4 (1.14-61.96)
FAM198A	1	1	8.87 (0.55-142.69)	6.68 (0.77-57.64)
FAM46B	1	0	Inf	12.96 (1.73-97.36)
FAM69C	1	1	8.77 (0.54-141.09)	55.06 (6.38-474.78)
FAM71C	1	0	Inf	12.38 (1.66-92.56)
FAM83B	1	0	Inf	24.98 (3.2-194.98)
FBLN2	1	0	Inf	23.92 (3.01-190.3)
FBXO39	1	0	Inf	25 (3.2-195.19)
FEZ2	1	0	Inf	16.92 (2.22-129.12)
FMN1	1	1	9.03 (0.56-145.28)	11.76 (1.57-88.1)
FNDC9	1	0	Inf	27.15 (3.45-213.73)
FOXRED1	1	0	Inf	5.92 (0.81-43.26)
FREM1	1	1	9.04 (0.56-145.41)	7.71 (1.04-56.84)
FXYD7	1	0	Inf	275.78 (17.15-4434.6)
FZD2	1	0	Inf	137.23 (12.36-1523.48)
FZD6	1	0	Inf	14.33 (1.9-107.9)
GALNT3	1	0	Inf	17.88 (2.34-136.42)
GAS2L2	1	1	9.05 (0.56-145.54)	12.11 (1.62-90.71)
GCC2	1	0	Inf	6.76 (0.92-49.55)
GCNT2	1	0	Inf	10.19 (1.37-75.57)
GFPT2	1	0	Inf	8.16 (1.11-60.21)
GLIPR1L2	1	0	Inf	5.11 (0.7-37.27)
GLIPR2	1	0	Inf	8.38 (1.14-61.81)
GOLIM4	1	0	Inf	10.07 (1.36-74.73)
GPR149	1	0	Inf	12.96 (1.73-97.1)
GPR37L1	1	0	Inf	33.52 (4.16-270.1)
GPSM2	1	1	9.03 (0.56-145.34)	5.72 (0.78-41.81)
GRAMD2	1	0	Inf	25 (3.2-195.19)
GRIN2C	1	0	Inf	17.68 (2.28-137.03)
GSTA5	1	0	Inf	6.4 (0.87-46.87)
GUCA1C	1	0	Inf	30.12 (3.79-239.59)
HAGH	1	1	8.43 (0.52-135.64)	15.13 (2-114.27)
HAUS2	1	0	Inf	38.01 (4.64-311.28)
HECW1	1	0	Inf	45.31 (5.41-379.23)
HIRIP3	1	1	9.04 (0.56-145.48)	30.24 (3.8-240.58)
HMGCLL1	1	0	Inf	20.39 (2.65-157.09)
HPS4	1	1	9.04 (0.56-145.48)	10.38 (1.4-77.11)
HPX	1	0	Inf	19.47 (2.54-149.21)
ICE1	1	0	Inf	36.76 (4.49-301.04)
IDH3A	1	0	Inf	137.79 (12.41-1529.79)
IFT88	1	0	Inf	8.7 (1.18-64.28)
IL1RL1	1	1	9.05 (0.56-145.54)	27.29 (3.47-214.87)
IL20RB	1	0	Inf	21.04 (2.73-162.11)
IMPG2	2	0	Inf	20.94 (4.91-89.21)

INO80E	1	0	Inf	14.25 (1.81-112.22)
INSM2	1	0	Inf	44.8 (5.19-386.31)
INSRR	2	1	18.22 (1.64-202.4)	15.93 (3.78-67.12)
INTS1	1	1	8.88 (0.55-142.95)	10.45 (1.4-78.02)
INTU	1	1	9.04 (0.56-145.48)	11.39 (1.53-84.87)
IQUB	1	1	9.05 (0.56-145.54)	5.84 (0.8-42.71)
IRGM	1	0	Inf	35.14 (2.18-565.15)
KATNB1	1	0	Inf	22.57 (2.91-174.93)
KCNH1	1	0	Inf	38.99 (4.76-319.33)
KCNH5	1	1	9.04 (0.56-145.41)	8.47 (1.15-62.45)
KCNJ1	1	0	Inf	10.12 (1.36-75.1)
KCNJ14	1	1	8.9 (0.55-143.15)	40.69 (4.86-340.55)
KCNQ1	1	1	9.04 (0.56-145.41)	8.34 (1.13-61.52)
KCTD19	1	1	9.04 (0.56-145.48)	11.46 (1.54-85.36)
KDELR3	1	1	8.62 (0.54-138.7)	5 (0.69-36.4)
KIAA1551	1	0	Inf	8.79 (1.19-64.93)
KLHDC2	1	0	Inf	19.62 (2.55-151.16)
KLHL21	1	0	Inf	49.24 (5.46-443.81)
KLHL25	1	0	Inf	10.88 (1.46-80.97)
KLHL28	1	0	Inf	263.8 (16.41-4242.04)
KLK1	1	0	Inf	14.42 (1.91-108.55)
KY	1	0	Inf	15.96 (2.09-121.79)
LDHAL6A	1	0	Inf	25.01 (3.2-195.25)
LDLRAD2	1	0	Inf	40.48 (4.69-349.06)
LEMD1	1	0	Inf	39.88 (4.77-333.77)
LIG1	1	0	Inf	11.42 (1.53-85.27)
LIG4	1	1	9.04 (0.56-145.48)	5.37 (0.74-39.18)
LIPI	1	0	Inf	17.77 (2.33-135.59)
LMOD2	1	0	Inf	19.28 (2.42-153.38)
LNX1	1	1	9.04 (0.56-145.48)	8.41 (1.14-62.07)
LRRFIP2	1	1	9.04 (0.56-145.48)	10.18 (1.37-75.49)
LTBP2	1	0	Inf	14.91 (1.97-112.9)
MAG	1	0	Inf	32.73 (4.06-263.72)
MAN1B1	1	1	9.05 (0.56-145.54)	6.05 (0.83-44.31)
MAP2K3	1	0	Inf	6.65 (0.91-48.75)
MAP3K13	2	0	Inf	39.18 (8.81-174.27)
MAP3K9	1	1	9 (0.56-144.88)	54.27 (6.29-467.97)
MATN3	1	0	Inf	23.45 (3-183.02)
MBOAT1	1	0	Inf	14.03 (1.86-105.68)
MCAT	1	0	Inf	15.34 (2.03-116.22)
MCMDC2	1	0	Inf	6.91 (0.94-50.79)
MEI1	1	0	Inf	5.19 (0.71-37.93)
METTL4	1	0	Inf	13.53 (1.8-101.59)
MIIP	1	1	8.35 (0.52-134.38)	5.48 (0.75-40.06)
MMS22L	1	0	Inf	22.23 (2.87-172.34)
MOV10L1	2	2	9.11 (1.27-65.25)	13.29 (3.18-55.6)
MRC2	1	0	Inf	43.38 (5.03-374.08)
MRPL39	1	1	9.04 (0.56-145.48)	17.16 (2.26-130.45)
MS4A14	1	0	Inf	5.33 (0.73-38.89)
MS4A7	1	0	Inf	68.78 (7.63-619.9)

MSH5	1	1	9.04 (0.56-145.48)	6.11 (0.83-44.68)
MSR1	1	0	Inf	8.18 (1.11-60.31)
MT1F	1	0	Inf	55.07 (6.39-474.93)
MTERF4	1	0	Inf	10.17 (1.37-75.43)
MTIF2	1	0	Inf	9.8 (1.32-72.63)
MTMR9	1	0	Inf	68.72 (7.63-619.34)
MUC15	1	1	9.04 (0.56-145.48)	16.95 (2.23-128.79)
MYCBPAP	1	0	Inf	18.99 (2.46-146.3)
MYO16	1	0	Inf	17.95 (2.34-137.59)
MYO3B	2	2	9.11 (1.27-65.25)	7.15 (1.74-29.45)
NAA38	1	0	Inf	16.46 (2.17-125.07)
NARS	1	0	Inf	20.38 (2.65-157.05)
NARS2	1	0	Inf	34.29 (4.26-276.25)
NBEA	1	0	Inf	24.2 (3.1-188.88)
NCAPH	1	0	Inf	47.57 (5.52-410.2)
NCF4	2	0	Inf	8.21 (1.99-33.88)
NCOR2	1	0	Inf	10.11 (1.35-75.6)
NDST4	1	1	9.04 (0.56-145.41)	45.03 (5.38-376.83)
NDUFAF6	1	0	Inf	10.33 (1.39-76.88)
NEK8	1	0	Inf	15.26 (2.02-115.2)
NEU2	1	1	8.76 (0.54-140.89)	33.33 (4.14-268.51)
NEXN	1	0	Inf	9.29 (1.25-68.71)
NGB	1	0	Inf	Inf
NLRP11	1	0	Inf	16.15 (2.13-122.33)
NLRP4	1	1	9.02 (0.56-145.21)	20.95 (2.72-161.41)
NLRX1	2	1	17.95 (1.62-199.44)	11.43 (2.75-47.57)
NMNAT3	1	0	Inf	7.83 (1.06-57.65)
NOL7	1	1	8.98 (0.56-144.48)	19.72 (2.54-152.88)
NOSTRIN	1	1	9.04 (0.56-145.41)	11.37 (1.53-84.73)
NPBWR1	1	0	Inf	8.73 (1.17-64.96)
NPR1	1	0	Inf	20.88 (2.69-161.88)
NRL	1	0	Inf	33.08 (4.11-266.54)
NUPL2	1	0	Inf	7.25 (0.98-53.31)
NXPE1	1	0	Inf	38.98 (4.76-319.24)
OGFOD1	1	1	9.04 (0.56-145.48)	16.16 (2.13-122.41)
OGFR	1	0	Inf	21.83 (2.71-175.88)
OR11H6	1	0	Inf	8.55 (1.16-63.08)
OR12D3	1	0	Inf	19.93 (2.59-153.55)
OR13A1	1	0	Inf	91.14 (9.41-882.41)
OR2AE1	1	0	Inf	33.96 (4.22-273.62)
OR51Q1	1	0	Inf	25.05 (3.21-195.54)
OR52E4	1	0	Inf	39.32 (4.8-322.01)
OR52E6	1	0	Inf	22.93 (2.96-177.7)
OR6Q1	1	0	Inf	24.82 (3.18-193.77)
OR9I1	1	0	Inf	19.63 (2.56-150.43)
OSBPL1A	1	1	9.04 (0.56-145.48)	14.44 (1.92-108.74)
OSMR	1	0	Inf	8.54 (1.16-63.01)
PABPC4	1	0	Inf	5.95 (0.81-43.47)
PAG1	1	0	Inf	45.6 (5.45-381.61)
PALLD	1	1	9.04 (0.56-145.48)	7.28 (0.99-53.54)

PAX4	1	0	Inf	10.9 (1.45-81.88)
PBK	1	0	Inf	14.14 (1.88-106.46)
PCCB	1	0	Inf	13.59 (1.81-102.05)
PCDHB10	1	1	9.04 (0.56-145.48)	12.31 (1.65-92.08)
PCDHGB6	1	0	Inf	7.04 (0.96-51.71)
PCDHGC5	1	1	9.05 (0.56-145.54)	18.92 (2.47-145.03)
PCNX	1	0	Inf	19.54 (2.55-149.77)
PDK4	1	0	Inf	12.29 (1.64-92.13)
PDZD8	1	0	Inf	273.85 (17.03-4403.63)
PGLS	1	0	Inf	48.32 (5.36-435.52)
PHIP	1	0	Inf	37.31 (4.56-305.57)
PHKB	2	0	Inf	14.01 (3.35-58.69)
PIBF1	1	0	Inf	8.24 (1.12-60.73)
PIGC	1	0	Inf	24.95 (3.2-194.78)
PIGL	1	1	9.04 (0.56-145.48)	27.55 (3.5-216.89)
PLCH1	2	0	Inf	34.27 (7.79-150.68)
PLVAP	1	1	9.04 (0.56-145.48)	91.05 (9.4-881.55)
POT1	1	0	Inf	14.02 (1.86-105.59)
PPP1R32	1	1	9.05 (0.56-145.54)	5.7 (0.78-41.74)
PPP3CC	1	0	Inf	18.05 (2.37-137.75)
PRMT3	1	1	9.05 (0.56-145.54)	10.96 (1.47-81.64)
PRR16	1	0	Inf	93.45 (8.42-1037.52)
PRR30	1	0	Inf	20.68 (2.68-159.29)
PYGM	1	0	Inf	10.45 (1.41-77.66)
RAB3GAP2	1	0	Inf	22.5 (2.9-174.38)
RBM14	1	0	Inf	Inf
RBM23	1	0	Inf	10.97 (1.47-81.74)
RFX6	1	0	Inf	21.18 (2.75-163.21)
RGS12	1	0	Inf	5.09 (0.7-37.09)
RHOT1	1	0	Inf	19.47 (2.54-149.26)
RNASE12	1	0	Inf	9.83 (1.33-72.82)
RNF141	1	0	Inf	55.03 (6.38-474.57)
RNLS	1	0	Inf	11.44 (1.54-85.25)
ROBO1	1	0	Inf	13.54 (1.8-101.97)
RPP38	1	1	9.04 (0.56-145.48)	27.42 (3.48-215.9)
RRH	1	0	Inf	10.11 (1.36-75.02)
RSF1	1	0	Inf	Inf
SCN9A	1	0	Inf	7.65 (1.04-56.45)
SDR39U1	1	0	Inf	12.78 (1.7-95.99)
SEC31B	2	2	9.11 (1.27-65.25)	5.16 (1.26-21.13)
SELL	1	0	Inf	60.58 (6.26-586.53)
SEMA3D	1	0	Inf	26.72 (3.39-210.38)
SETD6	1	0	Inf	11.06 (1.48-82.44)
SFRP4	1	0	Inf	24.96 (3.2-194.85)
SGCZ	1	0	Inf	22.41 (2.87-174.92)
SGSM2	1	1	8.74 (0.54-140.56)	13 (1.73-97.65)
SHB	1	0	Inf	127.3 (7.92-2047.13)
SHC1	1	0	Inf	39.11 (4.78-320.3)
SIRT5	1	1	9.04 (0.56-145.48)	15.08 (2-113.88)
SKA1	1	1	9.02 (0.56-145.14)	8.29 (1.13-61.12)

SLC12A9	1	1	8.86 (0.55-142.62)	12.04 (1.61-90.22)
SLC15A3	1	0	Inf	8.32 (1.13-61.38)
SLC17A5	1	0	Inf	8.05 (1.09-59.3)
SLC22A15	1	1	9.03 (0.56-145.34)	22.55 (2.89-176)
SLC25A26	1	1	9.04 (0.56-145.48)	9.69 (1.29-72.8)
SLC26A7	1	0	Inf	17.98 (2.36-137.22)
SLC28A3	1	0	Inf	14.39 (1.91-108.36)
SLC36A2	2	1	18.23 (1.64-202.58)	19.71 (4.64-83.65)
SLC41A3	1	0	Inf	19.28 (2.52-147.79)
SLC4A1AP	1	0	Inf	10.17 (1.37-75.46)
SLC6A16	1	1	9.04 (0.56-145.41)	6.52 (0.89-47.72)
SLC6A5	1	0	Inf	17.21 (2.26-131.32)
SLC7A9	1	0	Inf	8.59 (1.16-63.34)
SLC9A5	1	0	Inf	8.56 (1.16-63.17)
SLX4	1	1	9.02 (0.56-145.21)	7.27 (0.99-53.4)
SLX4IP	1	0	Inf	19.53 (2.55-149.69)
SMOC2	2	0	Inf	47.8 (10.48-217.96)
SMPD2	1	0	Inf	7.63 (1.04-56.08)
SNAPC1	1	0	Inf	30.38 (3.82-241.62)
SNX1	1	1	9.03 (0.56-145.34)	67.49 (7.49-608.25)
SPAG4	2	0	Inf	33.58 (7.6-148.46)
SPHKAP	1	0	Inf	39.21 (4.79-321.11)
SPINK1	1	0	Inf	27.08 (3.4-215.38)
SPINK4	1	0	Inf	274.86 (17.09-4419.85)
SRMS	1	0	Inf	16.85 (2.19-129.82)
SRPRB	1	0	Inf	27.32 (3.47-215.07)
ST6GALNAC2	1	0	Inf	7.52 (1.02-55.37)
STEAP2	1	1	9.04 (0.56-145.48)	6.19 (0.85-45.32)
STEAP4	1	0	Inf	10.88 (1.46-80.9)
SULF1	1	0	Inf	54.85 (6.36-473.02)
SULT1B1	1	0	Inf	15.77 (2.08-119.45)
SYT5	1	1	9.04 (0.56-145.48)	9.04 (1.22-67.28)
SYT6	1	0	Inf	90.69 (9.37-878.03)
SYVN1	1	0	Inf	Inf
SZT2	1	1	9.03 (0.56-145.34)	4.98 (0.68-36.3)
TATDN2	1	0	Inf	20.84 (2.71-160.58)
TBC1D32	1	1	9.03 (0.56-145.34)	5.57 (0.76-40.69)
TBX19	1	1	9.03 (0.56-145.34)	11.94 (1.6-89.1)
TEDDM1	1	0	Inf	27.51 (3.49-216.59)
TEK	1	0	Inf	137.46 (12.38-1526.07)
TEKT5	1	0	Inf	5.17 (0.71-37.67)
TGFBR2	1	0	Inf	91.45 (9.45-885.42)
TMC2	1	0	Inf	5.72 (0.78-41.8)
TMED3	1	0	Inf	9.52 (1.28-70.51)
TMEM116	1	0	Inf	19.49 (2.54-149.42)
TMEM132B	1	0	Inf	47.72 (5.53-411.49)
TMEM144	2	1	18.23 (1.64-202.58)	16.78 (3.98-70.71)
TMEM161A	1	0	Inf	39.46 (4.71-330.24)
TMEM5	1	0	Inf	29.39 (3.65-236.78)
TMEM70	1	0	Inf	9.47 (1.28-70.11)

TMEM8C	1	0	Inf	68.54 (7.61-617.72)
TNFAIP6	1	1	9.03 (0.56-145.34)	8.08 (1.1-59.5)
TOM1	1	0	Inf	13.13 (1.74-98.86)
TP53	1	0	Inf	100.95 (9.09-1120.76)
TP53I3	1	0	Inf	8.09 (1.1-59.66)
TPP1	1	0	Inf	10.2 (1.38-75.67)
TREML1	1	1	9.04 (0.56-145.48)	22.7 (2.93-175.98)
TRIM5	1	1	9.04 (0.56-145.48)	7 (0.95-51.34)
TRIP11	4	3	12.29 (2.72-55.58)	24.68 (8.74-69.71)
TRPA1	3	5	5.49 (1.3-23.27)	12.02 (3.73-38.74)
TSNAXIP1	4	1	37.09 (4.11-334.6)	19.05 (6.79-53.41)
TTC14	1	1	9.02 (0.56-145.21)	10.31 (1.39-76.68)
TTC37	1	1	9.04 (0.56-145.41)	7.15 (0.97-52.52)
TTLL1	1	0	Inf	10.97 (1.47-81.58)
TTLL12	1	0	Inf	13.42 (1.79-100.79)
TULP3	2	1	18.23 (1.64-202.58)	11.33 (2.71-47.43)
TXNDC12	1	0	Inf	84.55 (8.73-818.57)
UBE4B	1	0	Inf	54.44 (6.31-469.46)
UCP2	1	0	Inf	10.05 (1.35-74.67)
UGGT1	1	0	Inf	15.54 (2.05-117.68)
UIMC1	2	2	9.11 (1.27-65.27)	69.41 (14.59-330.25)
UNKL	1	1	8.74 (0.54-140.63)	9.11 (1.17-71.14)
URB1	2	1	17.81 (1.6-197.83)	21.93 (3.63-132.47)
URB2	1	1	9.03 (0.56-145.34)	5.6 (0.77-40.89)
UROS	1	0	Inf	19.27 (2.51-147.72)
USP20	1	0	Inf	14.65 (1.94-110.64)
USP6NL	1	0	Inf	20.08 (2.57-156.77)
UTS2	1	0	Inf	12.51 (1.67-93.52)
VIM	1	0	Inf	Inf
VNN1	1	1	9.04 (0.56-145.48)	22.76 (2.94-176.4)
VPS16	1	0	Inf	24.95 (3.2-194.78)
VSTM2B	1	0	Inf	14.96 (1.35-166.16)
XRCC2	1	0	Inf	12.95 (1.73-97.07)
XRCC6BP1	1	0	Inf	20.99 (2.72-161.73)
ZBTB9	1	0	Inf	30.09 (3.78-239.35)
ZCCHC7	1	0	Inf	24.93 (3.19-194.62)
ZIM2	1	0	Inf	25.04 (3.21-195.48)
ZKSCAN8	1	1	9.04 (0.56-145.48)	30.41 (3.82-241.92)
ZMYND8	1	0	Inf	272.81 (16.97-4386.92)
ZNF146	1	0	Inf	Inf
ZNF154	1	0	Inf	18.35 (2.4-140.01)
ZNF177	1	0	Inf	11.61 (1.55-87.19)
ZNF224	2	3	6.06 (1-36.66)	13.74 (3.28-57.48)
ZNF234	1	1	9.05 (0.56-145.54)	8.57 (1.16-63.23)
ZNF300	1	0	Inf	16.37 (2.15-124.42)
ZNF302	1	0	Inf	17.35 (2.27-132.42)
ZNF354A	1	1	9.04 (0.56-145.48)	39.1 (4.77-320.23)
ZNF391	1	1	9.04 (0.56-145.48)	15.29 (2.02-115.42)
ZNF484	1	0	Inf	18.3 (2.4-139.61)
ZNF567	1	0	Inf	27.08 (3.44-213.23)

ZNF607	2	2	9.11 (1.27-65.25)	9.9 (2.39-41.02)
ZNF716	2	0	Inf	18.69 (4.4-79.48)
