# Supplementary material for: Whole exome sequencing implicates eye development, the unfolded protein response and plasma membrane homeostasis in primary open-angle glaucoma
Source: PLoS One. 2017 Mar 6;12(3):e0172427. doi: 10.1371/journal.pone.0172427 (PMC5338784; doi:10.1371/journal.pone.0172427)
Supplement: S5 Table — (PDF) [file pone.0172427.s007.pdf]

S5 Table: List of enriched genes for normal-tension glaucoma cohort under a predicted pathogenic model

Headings:
Gene: HGNC gene name
NTG: Number of cases in normal-tension glaucoma cohort
CTRL: Number of cases in local and AOGC controls
NTG CTRL OR (95% CI): Odds ratio of normal-tension glaucoma cohort compared to controls
NTG NFE OR (95% CI): Odds ratio of normal-tension glaucoma cohort compared to non-Finnish European ExAC public domain data

Gene	NTG	CTRL	NTG CTRL OR (95% CI)	NTG NFE OR (95% CI)
A1CF	1	1	17.08 (1.06-276.19)	5.96 (0.82-43.41)
ABHD6	1	0	Inf	12.08 (1.64-89.04)
ACKR1	1	4	4.25 (0.47-38.62)	6.66 (0.91-48.58)
ACOX1	2	7	4.93 (1-24.24)	4.93 (1.2-20.3)
ADAT2	1	1	17.09 (1.06-276.44)	8.25 (1.13-60.36)
AGGF1	2	2	12.39 (1.72-89.42)	8.05 (1.95-33.26)
AKT1S1	1	3	5.04 (0.52-49.16)	13.53 (1.8-101.49)
ALDOA	2	5	6.91 (1.31-36.31)	7.36 (1.78-30.41)
AMBP	2	3	11.35 (1.86-69.15)	7.07 (1.71-29.15)
AMFR	2	0	Inf	8.39 (2.03-34.68)
ANKRA2	1	4	4.17 (0.46-37.85)	6.44 (0.88-47)
ANKRD34A	1	0	Inf	15.32 (2.06-113.91)
ANKRD46	1	0	Inf	28.37 (3.72-216.4)
AP1B1	2	3	11.4 (1.87-69.44)	5.41 (1.31-22.28)
AP1G1	1	0	Inf	9.72 (1.32-71.38)
AP1S3	1	1	17.09 (1.06-276.44)	15.08 (2.03-111.85)
APOBEC3A	1	0	Inf	18.33 (2.45-136.93)
APOH	1	2	8.54 (0.76-95.4)	7.72 (1.06-56.44)
ARHGAP26	1	2	8.54 (0.76-95.43)	8.04 (1.1-58.84)
ARL14EPL	2	0	Inf	11.35 (2.16-59.62)
ARMC2	2	3	11.54 (1.89-70.32)	5.73 (1.39-23.62)
ATG9A	2	8	4.27 (0.89-20.54)	8.86 (2.14-36.64)
ATP5G2	1	0	Inf	11.01 (1.49-81.18)
ATP6AP1L	1	0	Inf	14.86 (2.01-110.13)
ATP6V1D	1	1	17.09 (1.06-276.44)	7.3 (1-53.38)
ATP9A	2	2	17.23 (2.39-124.38)	6.38 (1.55-26.29)
AURKAIP1	1	0	Inf	8.94 (1.22-65.59)
AURKB	1	3	5.68 (0.58-55.42)	4.56 (0.63-33.18)
AZIN1	1	2	8.38 (0.75-93.59)	12.34 (1.67-91.04)
BHLHA9	1	0	Inf	Inf
BLOC1S4	1	0	Inf	21.58 (2.81-165.78)
BMPR1A	2	1	35.01 (3.13-391.34)	14.98 (3.59-62.4)
BRD3	1	2	7.02 (0.63-78.5)	11.12 (1.51-81.88)
BTBD2	1	0	Inf	4.29 (0.59-31.19)
BTG4	1	0	Inf	9.73 (1.33-71.43)
C10orf95	1	0	Inf	36.5 (4.64-286.89)
C11orf91	1	0	Inf	13.16 (1.18-147.38)
C12orf4	2	5	6.92 (1.32-36.36)	7.67 (1.86-31.68)
C12orf43	1	0	Inf	7.82 (1.07-57.28)
C16orf45	2	1	33.54 (3-374.9)	11.16 (2.68-46.38)
C16orf70	1	3	5.69 (0.58-55.45)	9.13 (1.24-66.91)
C17orf105	1	1	17.09 (1.06-276.44)	21.82 (2.24-212.58)
C17orf49	1	1	17.09 (1.06-276.44)	7.13 (0.98-52.14)
C19orf52	1	2	5.63 (0.5-62.97)	6.63 (0.9-48.65)
C1orf186	1	2	8.54 (0.76-95.38)	8.52 (1.16-62.45)
C20orf197	1	3	5.66 (0.58-55.21)	6.4 (0.88-46.82)
C20orf62	2	0	Inf	15.58 (2.97-81.82)
C21orf59	1	1	17.09 (1.06-276.44)	5.16 (0.71-37.6)
C3orf14	2	1	34.71 (3.11-388.02)	55.12 (12.57-241.63)

C3orf18	1	0	Inf	8.7 (1.18-63.94)
C4orf32	1	0	Inf	12.96 (1.74-96.47)
CA11	1	2	8.52 (0.76-95.25)	7.48 (1.02-54.8)
CA13	2	2	17.3 (2.4-124.86)	12.72 (3.06-52.84)
CADM2	1	0	Inf	23.49 (3.12-176.91)
CAPZB	1	0	Inf	11.27 (1.53-83.17)
CARM1	1	2	8.33 (0.74-93.04)	20.51 (2.74-153.68)
CATSPER4	2	7	4.93 (1-24.21)	10.15 (2.45-42.04)
CBLL1	1	1	17.09 (1.06-276.44)	15.72 (2.12-116.65)
CBR4	1	1	17.05 (1.05-275.81)	5.25 (0.72-38.26)
CCDC127	1	0	Inf	9.44 (1.29-69.24)
CCDC179	1	0	Inf	Inf
CCDC6	1	1	17.09 (1.06-276.44)	15.31 (2.06-113.74)
CCDC94	1	1	17 (1.05-274.93)	15.42 (2.07-115.06)
CCL18	1	2	8.53 (0.76-95.37)	43.37 (5.56-338.52)
CCL5	2	6	5.76 (1.14-29.13)	10.97 (2.64-45.5)
CCNE1	1	2	8.54 (0.76-95.43)	10.52 (1.43-77.44)
CCNE2	1	0	Inf	6.55 (0.9-47.78)
CCSAP	1	0	Inf	21.02 (2.79-158.63)
CDKN2AIP	1	1	17.09 (1.06-276.44)	9.1 (1.24-66.81)
CDS1	1	4	4.26 (0.47-38.67)	5.2 (0.71-37.88)
CH25H	1	2	8.54 (0.76-95.45)	18.25 (2.44-136.55)
CHMP4A	2	2	17.33 (2.4-125.05)	8.74 (2.11-36.12)
CHRM4	1	4	4.26 (0.47-38.67)	7.97 (1.09-58.39)
CHST10	1	2	8.54 (0.76-95.43)	9.44 (1.29-69.26)
CHST13	1	0	Inf	10 (1.34-74.63)
CHSY3	1	0	Inf	6.21 (0.85-45.32)
CIAO1	1	2	8.54 (0.76-95.43)	5.93 (0.81-43.21)
CLEC4C	1	0	Inf	5.52 (0.76-40.2)
CLRN2	1	4	4.24 (0.47-38.51)	10.38 (1.41-76.28)
CNNM3	2	3	7 (1.15-42.81)	10.89 (2.62-45.2)
CNR1	1	2	8.54 (0.76-95.43)	8.48 (1.16-62.09)
CNRIP1	2	2	9.12 (1.26-65.86)	31.2 (7.24-134.53)
COG2	2	2	17.33 (2.4-125.09)	10.19 (2.46-42.21)
COMMD2	1	1	17.09 (1.06-276.44)	9.34 (1.27-68.53)
COQ10A	1	4	4.26 (0.47-38.69)	5.67 (0.78-41.34)
CORO1C	1	2	4.79 (0.43-53.59)	8.95 (1.22-65.7)
COX17	1	0	Inf	122.67 (13.52-1112.7)
COX5A	1	0	Inf	8.13 (1.11-59.48)
CPSF4L	1	4	4.26 (0.47-38.69)	4.14 (0.54-31.72)
CREG1	1	1	17.08 (1.06-276.19)	13.74 (1.86-101.76)
CRIPT	1	1	17.09 (1.06-276.44)	15.16 (2.04-112.42)
CRISPLD1	2	0	Inf	7.27 (1.76-30.02)
CRTC1	1	2	8.54 (0.76-95.43)	4.23 (0.58-30.74)
CRX	1	1	16.63 (1.03-268.99)	7.17 (0.98-52.37)
CST3	1	0	Inf	35.57 (4.38-288.54)
CST6	1	2	6.01 (0.54-67.16)	87.06 (10.03-755.73)
CTDSP1	1	0	Inf	18.73 (2.5-140.29)
CTSD	1	0	Inf	7.38 (1.01-53.98)
CTXN3	1	1	17.09 (1.06-276.44)	40.08 (5.17-310.9)

CXCR6	1	4	4.26 (0.47-38.69)	11.08 (1.51-81.51)
CYGB	1	0	Inf	5.88 (0.81-42.88)
CYP17A1	1	4	4.26 (0.47-38.68)	6.36 (0.87-46.43)
DAPK3	2	2	16.9 (2.34-121.94)	6.94 (1.68-28.69)
DBR1	2	7	4.93 (1-24.23)	8.28 (2-34.21)
DCD	1	1	17.09 (1.06-276.44)	13.28 (1.79-98.78)
DCK	1	0	Inf	21.5 (2.86-161.3)
DCTN3	1	0	Inf	15.31 (2.06-113.88)
DCTN5	1	0	Inf	26.81 (3.52-204.51)
DCTPP1	1	2	8.42 (0.75-94.09)	15.34 (2.07-113.83)
DEFB135	1	2	8.54 (0.76-95.43)	8.08 (1.1-59.15)
DGKA	1	4	4.26 (0.47-38.67)	5.46 (0.75-39.78)
DMRTA2	1	0	Inf	10.79 (1.44-80.94)
DNAJA2	1	1	17.08 (1.06-276.19)	12.89 (1.75-95.2)
DNAJB14	2	1	24.84 (2.22-277.68)	9.79 (2.36-40.53)
DNAJC5G	1	3	5.69 (0.58-55.45)	10.39 (1.41-76.37)
DPM1	1	1	17.08 (1.06-276.19)	16.24 (2.19-120.68)
DRG1	1	0	Inf	25.99 (3.44-196.54)
DUSP14	1	1	17.09 (1.06-276.44)	7.16 (0.98-52.29)
DUSP4	1	0	Inf	6.26 (0.86-45.77)
DYNC1I1	1	2	8.54 (0.76-95.43)	7.31 (1-53.51)
DYNLRB1	1	0	Inf	47.47 (5.99-376.29)
EBF3	1	2	8.51 (0.76-95.08)	10.31 (1.4-75.82)
ECH1	2	5	6.91 (1.31-36.32)	13.22 (3.17-55.05)
ECHDC3	2	1	22.76 (2.04-254.52)	8.56 (2.07-35.4)
EFR3B	1	1	17.1 (1.06-276.57)	4.81 (0.62-37.14)
EGFL8	2	2	16.25 (2.25-117.27)	11.82 (2.85-49.07)
EGLN1	1	0	Inf	25.86 (3.38-197.96)
EHD1	1	4	4.25 (0.47-38.6)	4.7 (0.65-34.2)
EHD4	2	6	5.72 (1.13-28.94)	8.77 (2.12-36.29)
EID1	1	0	Inf	24.01 (3.18-181.2)
EIF2S1	1	0	Inf	18.52 (2.48-138.16)
EIF4H	1	0	Inf	12.72 (1.72-93.92)
ELP6	1	4	4.26 (0.47-38.69)	6.56 (0.9-47.85)
EN1	1	4	4.1 (0.45-37.22)	9.08 (1.23-66.7)
ENTPD1	1	3	5.69 (0.58-55.45)	5.67 (0.78-41.31)
EVI2A	1	0	Inf	11.5 (1.56-84.7)
EZH2	1	2	8.54 (0.76-95.43)	13.52 (1.83-99.94)
F10	1	2	8.37 (0.75-93.54)	6.71 (0.92-48.95)
FAM118B	1	2	8.54 (0.76-95.4)	7.84 (1.07-57.33)
FAM131A	1	2	8.15 (0.73-91.06)	6.42 (0.88-46.89)
FAM132B	1	0	Inf	5.01 (0.59-42.21)
FAM219A	1	2	8.34 (0.75-93.16)	16.27 (2.19-120.99)
FAM32A	2	2	17.34 (2.4-125.16)	28.23 (6.5-122.6)
FAM91A1	1	2	8.53 (0.76-95.29)	4.61 (0.63-33.52)
FAR2	1	3	5.69 (0.58-55.45)	7.98 (1.09-58.4)
FARSA	3	4	13.18 (2.89-60.16)	7.99 (2.49-25.67)
FBL	1	4	4.2 (0.46-38.12)	4.9 (0.67-35.71)
FBXO21	1	1	6.81 (0.42-110.19)	9.17 (1.25-67.39)
FBXO22	1	1	15.23 (0.94-246.27)	8.72 (1.19-64.05)

FBXO46	1	0	Inf	6.14 (0.84-44.85)
FBXW7	1	1	17.08 (1.06-276.19)	13.22 (1.79-97.78)
FIS1	1	2	8.51 (0.76-95.05)	13.89 (1.88-102.84)
FKBP2	1	2	8.52 (0.76-95.16)	7.26 (0.99-53.02)
FKBP7	1	4	4.26 (0.47-38.69)	6.18 (0.85-45.09)
FLOT2	2	5	6.9 (1.31-36.28)	8.29 (2-34.24)
FLRT2	1	2	8.54 (0.76-95.45)	6.17 (0.85-45)
FNTA	1	1	17.09 (1.06-276.32)	15.99 (2.15-119.18)
FOXG1	1	0	Inf	38.09 (4.8-301.91)
FOXH1	1	2	7.83 (0.7-87.51)	6.6 (0.9-48.37)
FOXO6	1	0	Inf	17.33 (1.55-193.57)
FRG2C	1	2	8.54 (0.76-95.47)	Inf
FST	1	0	Inf	17.95 (2.41-133.8)
GAL3ST3	1	0	Inf	4.67 (0.64-34.04)
GAPVD1	3	12	4.37 (1.2-15.87)	6.24 (1.95-20)
GCLM	1	1	17.08 (1.06-276.19)	7.22 (0.99-52.81)
GCNT1	1	2	8.54 (0.76-95.43)	8.36 (1.14-61.21)
GDNF	1	1	17.09 (1.06-276.32)	13.07 (1.76-96.86)
GET4	1	3	5.54 (0.57-53.99)	6.84 (0.94-49.97)
GIT2	1	4	4.26 (0.47-38.66)	5.18 (0.71-37.69)
GJA5	1	4	4.26 (0.47-38.65)	16.25 (2.19-120.73)
GJA8	2	4	8.62 (1.55-47.99)	11.73 (2.83-48.69)
GLRA1	1	3	5.68 (0.58-55.42)	7.43 (1.02-54.29)
GLYR1	1	0	Inf	9.18 (1.25-67.34)
GM2A	1	3	5.67 (0.58-55.25)	10.17 (1.38-74.86)
GNL3	1	3	5.69 (0.58-55.45)	5.88 (0.81-42.84)
GP9	1	0	Inf	8.97 (1.22-66.03)
GPAT2	1	1	16.99 (1.05-274.8)	6.17 (0.85-45.03)
GPATCH11	1	2	8.45 (0.76-94.42)	6.97 (0.94-51.43)
GPR132	1	4	4.23 (0.47-38.37)	8.76 (1.2-64.23)
GRAMD4	1	1	16.56 (1.02-267.86)	6.52 (0.89-47.57)
GRHL2	1	4	4.26 (0.47-38.69)	5.22 (0.72-38.01)
GRM7	1	3	5.68 (0.58-55.42)	4.7 (0.65-34.19)
GRP	1	0	Inf	8.45 (1.15-62.28)
GRXCR2	2	6	5.76 (1.14-29.12)	11.3 (2.73-46.87)
GUCD1	1	2	8.25 (0.74-92.17)	7.24 (0.99-52.98)
HCAR1	2	7	4.93 (1-24.22)	6.99 (1.69-28.82)
HCN1	1	1	16.73 (1.03-270.64)	11.72 (1.59-86.38)
HCST	1	2	8.41 (0.75-93.97)	50.82 (6.41-402.87)
HDAC3	1	0	Inf	24.78 (3.28-187.03)
HDAC9	3	4	13.19 (2.89-60.25)	6.57 (2.05-21.1)
HES6	1	0	Inf	10.74 (1.44-79.93)
HEXIM1	1	0	Inf	9.07 (1.24-66.53)
HIST1H2AI	1	0	Inf	8.02 (1.09-58.69)
HIST1H3B	1	1	16.76 (1.04-271.01)	22.44 (2.99-168.7)
HIST1H4A	1	1	17.09 (1.06-276.44)	6.2 (0.85-45.22)
HIST2H2AB	1	0	Inf	21.36 (2.85-160.3)
HLA-DMB	1	1	17.09 (1.06-276.44)	15.67 (2.11-116.55)
HLX	1	3	4.49 (0.46-43.83)	6.18 (0.84-45.2)
HMBS	2	4	8.66 (1.56-48.18)	10.27 (2.48-42.58)

HNRNPR	1	1	17.08 (1.06-276.19)	7.85 (1.07-57.39)
HOPX	1	1	13.2 (0.82-213.56)	9.04 (1.23-66.61)
HPGD	1	0	Inf	6.32 (0.87-46.17)
HSBP1	1	0	Inf	52.34 (6.21-440.96)
HSD17B3	1	2	8.54 (0.76-95.43)	6.46 (0.89-47.12)
IGF2BP1	1	0	Inf	13.07 (1.77-96.6)
IL10RA	1	0	Inf	6.92 (0.95-50.5)
IL17B	1	0	Inf	6.2 (0.85-45.27)
IL17F	2	0	Inf	14.22 (3.42-59.21)
IL34	1	3	5.62 (0.58-54.81)	7.66 (1.05-56.07)
IL36A	1	0	Inf	19.29 (2.58-144.09)
IMMP2L	1	0	Inf	13.98 (1.89-103.44)
IMPDH2	1	2	8.54 (0.76-95.4)	5.8 (0.8-42.29)
INS-IGF2	2	3	10.54 (1.73-64.26)	35.81 (8.3-154.4)
INTS7	2	1	34.7 (3.1-387.84)	9.74 (2.35-40.3)
ISL1	1	2	8.49 (0.76-94.9)	17.92 (2.41-133.57)
ITPA	2	1	34.72 (3.11-388.1)	21.76 (5.18-91.48)
KAT8	1	0	Inf	22.47 (2.98-169.19)
KBTBD2	1	0	Inf	6.63 (0.91-48.4)
KBTBD4	2	3	8.32 (1.36-50.7)	11.23 (2.71-46.56)
KBTBD6	1	1	16.8 (1.04-271.65)	6.83 (0.94-49.89)
KCNH1	2	0	Inf	14.17 (3.4-58.97)
KCNMA1	3	2	12.98 (2.13-79.2)	15.88 (4.89-51.58)
KEAP1	1	1	16.73 (1.03-270.64)	3.99 (0.55-29)
KIAA0040	1	1	17.08 (1.06-276.19)	9.14 (1.11-75.4)
KISS1	1	1	8.05 (0.5-130.23)	15.69 (2.05-120.1)
KLHL2	1	1	17.09 (1.06-276.44)	23.37 (3.1-175.98)
KNG1	3	1	52.92 (5.43-516.15)	12.19 (3.78-39.34)
KPNB1	1	0	Inf	39.52 (5.09-306.58)
KRTAP10-12	1	2	7.91 (0.71-88.36)	7.17 (0.98-52.42)
KRTAP20-1	1	0	Inf	11.53 (1.56-84.89)
L3MBTL3	1	1	17.09 (1.06-276.44)	7.92 (1.08-57.92)
LAMTOR3	1	0	Inf	39.72 (5.09-310.01)
LEPROT	1	1	9.88 (0.61-159.9)	8.28 (1.13-60.66)
LKAAEAR1	1	0	Inf	10.51 (1.37-80.43)
LMAN2L	1	2	8.54 (0.76-95.38)	5.21 (0.72-37.91)
LMBRD2	1	3	5.68 (0.58-55.39)	5.62 (0.77-40.94)
LMX1B	1	0	Inf	9.75 (1.32-71.88)
LOX	1	0	Inf	11.19 (1.52-82.55)
LPAR5	1	4	4.18 (0.46-37.98)	7.18 (0.97-52.97)
LPCAT2	3	9	5.48 (1.45-20.74)	9.04 (2.81-29.09)
LPPR1	1	2	8.54 (0.76-95.43)	19.28 (2.58-144.01)
LRRC14	1	0	Inf	5.53 (0.76-40.31)
LRRC25	1	0	Inf	7.31 (1-53.4)
LSM11	1	0	Inf	8.4 (1.15-61.56)
MAP2K7	1	2	8.19 (0.73-91.56)	7.73 (1.05-56.7)
MAP4K3	1	2	7.26 (0.65-81.19)	6.05 (0.83-44.09)
MAPKAPK2	1	1	16.33 (1.01-264.07)	18.51 (2.48-138.12)
MAPRE1	1	0	Inf	23.63 (3.14-177.92)
MARCH9	1	1	16.91 (1.05-273.41)	10.87 (1.47-80.27)

MB21D1	2	5	6.86 (1.31-36.07)	6.91 (1.67-28.52)
MDM4	1	2	8.53 (0.76-95.34)	6.01 (0.82-43.85)
MEA1	1	0	Inf	17.04 (2.29-126.83)
MED19	1	0	Inf	14.05 (1.9-103.96)
MED27	1	1	17.1 (1.06-276.57)	10.33 (1.4-76.16)
MEF2D	1	3	5.31 (0.54-51.73)	6.21 (0.85-45.31)
MEPCE	1	3	5.67 (0.58-55.31)	9.8 (1.33-72.13)
METTL14	1	2	8.53 (0.76-95.37)	9.55 (1.3-70.11)
METTL6	2	3	11.53 (1.89-70.27)	12.33 (2.96-51.31)
MFAP3L	1	2	8.54 (0.76-95.45)	13.64 (1.84-100.83)
MGAT4D	1	0	Inf	5.33 (0.61-46.31)
MICU2	1	1	17.07 (1.06-276.07)	7.12 (0.97-52.07)
MITF	1	2	8.54 (0.76-95.43)	12.16 (1.65-89.66)
MMD	1	1	17.09 (1.06-276.44)	23.28 (3.09-175.34)
MNX1	1	0	Inf	12.04 (1.6-90.5)
MRAP2	1	4	4.26 (0.47-38.69)	8.79 (1.2-64.4)
MRPS25	1	3	5.69 (0.58-55.45)	25.05 (3.3-190.49)
MRPS26	2	0	Inf	20.02 (4.75-84.43)
MRPS7	1	1	17.1 (1.06-276.57)	9.71 (1.32-71.29)
MS4A8	1	2	8.54 (0.76-95.43)	15.2 (2.05-112.71)
MYF5	1	3	5.66 (0.58-55.23)	15.17 (2.05-112.54)
MYLK2	2	3	11.53 (1.89-70.23)	13.49 (3.24-56.2)
MZB1	1	0	Inf	5.08 (0.69-37.33)
NAIP	1	3	5.75 (0.59-56.04)	8.17 (1.12-59.79)
NAT1	1	3	5.69 (0.58-55.45)	7.5 (1.02-54.95)
NDE1	1	1	17.09 (1.06-276.36)	5.03 (0.69-36.62)
NDNL2	1	1	17.08 (1.06-276.19)	13.3 (1.8-98.57)
NDUFB2	1	1	17.09 (1.06-276.44)	13.41 (1.81-99.52)
NDUFB8	2	3	11.56 (1.9-70.41)	14.73 (3.53-61.37)
NDUFC2-KCTD14	1	0	Inf	Inf
NEK6	1	4	4.23 (0.47-38.37)	7.84 (1.07-57.47)
NEUROD4	1	2	8.54 (0.76-95.43)	8.51 (1.16-62.31)
NFXL1	1	1	17.09 (1.06-276.32)	6.5 (0.89-47.49)
NHLRC1	1	2	6.16 (0.55-68.85)	11.75 (1.59-86.81)
NIT2	1	3	5.69 (0.58-55.45)	6.54 (0.9-47.73)
NKX2-6	1	1	13.23 (0.82-213.94)	4.61 (0.59-35.76)
NKX6-2	2	0	Inf	21.44 (5.08-90.4)
NOB1	1	0	Inf	6.19 (0.85-45.17)
NOS1AP	1	2	8.52 (0.76-95.16)	8.38 (1.14-61.4)
NOV	1	3	5.69 (0.58-55.45)	7.95 (1.09-58.16)
NOVA1	1	0	Inf	13.87 (1.87-102.79)
NPFFR1	1	1	17.09 (1.06-276.44)	6.21 (0.85-45.58)
NPTX2	1	2	8.32 (0.74-92.95)	7.6 (1.04-55.65)
NT5DC1	1	1	17.09 (1.06-276.44)	8.15 (1.11-59.65)
NT5DC3	1	3	5.69 (0.58-55.45)	4.43 (0.61-32.22)
NTMT1	1	0	Inf	12.3 (1.67-90.72)
NUB1	1	3	5.66 (0.58-55.14)	5.3 (0.73-38.69)
NUDT1	1	3	5.69 (0.58-55.45)	4.43 (0.61-32.21)
NUDT9	1	1	17.09 (1.06-276.44)	8.27 (1.13-60.58)
ONECUT1	1	2	7.42 (0.66-82.89)	6.28 (0.86-45.85)

ORAOV1	1	2	8.54 (0.76-95.38)	17.35 (2.33-129.15)
OSGIN2	1	1	17.09 (1.06-276.44)	13.29 (1.8-98.24)
OSTF1	1	2	8.52 (0.76-95.25)	6.21 (0.85-45.32)
OTUD3	1	1	9.67 (0.6-156.49)	14.86 (2-110.21)
OVOL1	1	3	5.63 (0.58-54.89)	12.99 (1.75-96.16)
PAGR1	1	3	5.17 (0.53-50.43)	6.97 (0.95-51.28)
PARK7	1	1	17.08 (1.06-276.19)	8.24 (1.13-60.33)
PARL	1	0	Inf	11.77 (1.59-86.86)
PAX6	1	0	Inf	13.41 (1.81-99.16)
PCBP4	1	3	5.59 (0.57-54.47)	5.13 (0.7-37.32)
PDE6H	1	0	Inf	11.11 (1.51-81.78)
PDPK1	2	2	15.77 (2.19-113.81)	38.82 (9.04-166.72)
PDS5B	1	2	8.54 (0.76-95.43)	8.17 (1.12-59.82)
PDX1	1	0	Inf	18.59 (2.46-140.61)
PDZD9	1	0	Inf	29.33 (3.85-223.67)
PEX11B	1	1	15.2 (0.94-245.82)	5.18 (0.71-37.77)
PHF21A	1	3	5.66 (0.58-55.2)	6.47 (0.89-47.25)
PHIP	1	1	17.09 (1.06-276.36)	6.1 (0.84-44.51)
PHOSPHO2	1	1	17.09 (1.06-276.44)	7.27 (0.99-53.1)
PHYHIP	1	1	16.31 (1.01-263.82)	11.41 (1.54-84.26)
PI4KB	1	2	8.53 (0.76-95.34)	10.73 (1.46-78.89)
PIK3CB	1	4	4.26 (0.47-38.65)	5.5 (0.76-40.07)
PIP4K2C	2	6	5.76 (1.14-29.13)	7.5 (1.82-30.96)
PKNOX1	1	1	17.09 (1.06-276.44)	10.96 (1.49-80.65)
PLAGL1	1	3	5.69 (0.58-55.45)	10.09 (1.37-74.12)
PLEKHO1	1	2	8.32 (0.74-93.01)	6 (0.82-43.78)
PNN	1	2	8.54 (0.76-95.45)	5.47 (0.75-39.83)
POLR2B	1	1	17.1 (1.06-276.57)	14.71 (1.99-109.03)
POLR3D	1	1	9.57 (0.59-154.85)	9.6 (1.31-70.49)
POLR3H	1	1	15.4 (0.95-249.04)	8.11 (1.11-59.35)
POU1F1	1	3	5.69 (0.58-55.45)	5.61 (0.77-40.87)
PPDPF	1	3	4.31 (0.44-42)	8.28 (1.13-60.75)
PPM1L	1	1	17.09 (1.06-276.44)	15.25 (2.06-113.08)
PPP2R5A	1	1	17.08 (1.06-276.19)	11.77 (1.59-86.93)
PRKG2	1	0	Inf	8.45 (1.15-61.91)
PRLR	1	2	6.07 (0.54-67.83)	8.11 (1.11-59.35)
PROP1	1	1	16.56 (1.02-267.86)	6.47 (0.89-47.22)
PRPF6	1	0	Inf	8.77 (1.2-64.28)
PRR16	1	4	4.26 (0.47-38.69)	8.72 (1.19-63.95)
PRR23A	1	3	5.61 (0.58-54.69)	12.31 (1.65-91.75)
PRSS8	1	2	8.46 (0.76-94.51)	9.02 (1.23-66.19)
PSMD12	1	2	8.53 (0.76-95.37)	17.72 (2.38-132.08)
PSMG2	1	1	17.09 (1.06-276.44)	9.89 (1.35-72.66)
PTAR1	1	3	5.69 (0.58-55.45)	6.32 (0.87-46.23)
PTBP1	1	2	8.46 (0.76-94.51)	10.74 (1.46-79.13)
PTPN12	1	4	4.26 (0.47-38.69)	5.11 (0.7-37.23)
PTRH2	1	3	5.69 (0.58-55.45)	22.6 (3.01-169.85)
PUSL1	2	2	16.97 (2.35-122.46)	7.23 (1.74-29.99)
PWWP2A	1	2	8.54 (0.76-95.48)	11.19 (1.51-82.77)
RAB29	1	1	17.08 (1.06-276.19)	14.07 (1.9-104.2)

RAB2A	1	0	Inf	105.85 (11.67-960.14)
RAB4B	1	2	8.39 (0.75-93.77)	4.12 (0.57-29.95)
RASD1	1	1	16.53 (1.02-267.35)	6.78 (0.93-49.66)
RASL10B	1	1	16.59 (1.03-268.36)	71.4 (8.66-588.71)
RBBP6	3	8	6.07 (1.57-23.46)	7.5 (2.33-24.07)
RCHY1	1	1	16.59 (1.03-268.24)	12.23 (1.66-90.28)
REC8	1	2	8.53 (0.76-95.34)	5.3 (0.73-38.57)
RGS21	1	2	8.52 (0.76-95.25)	6.53 (0.89-47.66)
RGS5	1	3	5.66 (0.58-55.2)	7.59 (1.04-55.5)
RIPK4	3	5	9.13 (2.13-39.1)	6.89 (2.15-22.11)
RMND1	1	4	4.26 (0.47-38.64)	4.29 (0.59-31.16)
RNASEL	4	8	8.9 (2.61-30.39)	6.86 (2.48-18.99)
RND3	1	1	17.09 (1.06-276.36)	10.59 (1.44-77.87)
RNF126	1	1	15.19 (0.94-245.64)	5.02 (0.68-36.8)
RNF130	1	2	8.54 (0.76-95.43)	15.42 (2.07-114.81)
RNF141	1	4	4.26 (0.47-38.69)	27.36 (3.61-207.47)
RNF19B	1	3	5.68 (0.58-55.4)	9.02 (1.23-66.18)
RNF34	1	2	8.54 (0.76-95.43)	10.27 (1.4-75.5)
RPL13	1	2	8.53 (0.76-95.34)	6.55 (0.9-47.89)
RPL8	1	0	Inf	18.15 (2.43-135.39)
RPS19BP1	1	2	8.26 (0.74-92.29)	18.45 (2.46-138.23)
RTN4IP1	2	3	11.56 (1.9-70.41)	7.79 (1.89-32.17)
S1PR2	1	0	Inf	6.92 (0.95-50.56)
SAAL1	1	2	8.54 (0.76-95.43)	5.74 (0.79-41.84)
SAP30L	1	1	17.09 (1.06-276.44)	15.28 (2.05-113.84)
SATB1	1	3	5.69 (0.58-55.45)	14.56 (1.96-107.9)
SDF4	2	1	33.41 (2.99-373.42)	11 (2.65-45.64)
SDS	1	4	4.15 (0.46-37.69)	4.69 (0.64-34.11)
SEC22C	1	2	8.52 (0.76-95.25)	9.4 (1.28-68.95)
SEPT12	4	10	7.08 (2.16-23.23)	8.67 (3.13-24.05)
SF3A1	1	2	5.99 (0.54-66.96)	9.01 (1.23-66.11)
SFRP2	1	0	Inf	19.85 (2.65-148.69)
SFT2D2	1	3	5.68 (0.58-55.41)	15.25 (2.05-113.27)
SIAH3	1	0	Inf	9.75 (1.33-71.58)
SKAP1	1	1	17.09 (1.06-276.44)	10.07 (1.37-73.99)
SLBP	1	0	Inf	21.9 (2.92-164.33)
SLC23A2	1	3	5.69 (0.58-55.44)	6.01 (0.82-43.78)
SLC30A2	1	0	Inf	6.67 (0.91-48.71)
SLC35B4	1	4	4.26 (0.47-38.69)	6.27 (0.86-45.74)
SLC35F3	1	3	5.68 (0.58-55.4)	12.24 (1.66-90.32)
SLC39A14	1	1	9.54 (0.59-154.35)	6.49 (0.89-47.35)
SLC40A1	1	1	17.09 (1.06-276.44)	14.85 (2-110.01)
SLC51B	1	3	5.68 (0.58-55.4)	14.39 (1.94-106.9)
SLCO3A1	1	3	5.68 (0.58-55.35)	5.09 (0.7-37.1)
SMARCA4	3	3	14.14 (2.8-71.52)	11.11 (3.44-35.85)
SMCO3	1	1	17.09 (1.06-276.44)	5.56 (0.76-40.5)
SMIM11	1	0	Inf	57.04 (7.12-456.81)
SMR3B	2	2	17.35 (2.4-125.2)	12.39 (2.98-51.44)
SNCG	1	1	16.87 (1.04-272.82)	9.19 (1.25-67.46)
SNX17	1	4	4.26 (0.47-38.69)	9.96 (1.36-73.16)

SNX22	1	3	5.68 (0.58-55.35)	23.58 (3.13-177.58)
SNX8	2	2	16.8 (2.33-121.27)	23.36 (5.52-98.82)
SOAT1	2	2	17.33 (2.4-125.06)	7.55 (1.83-31.15)
SP9	1	0	Inf	11.37 (1.38-93.8)
SPTY2D1	1	3	5.68 (0.58-55.41)	7.85 (1.07-57.44)
SRRM4	1	2	8.54 (0.76-95.43)	7.11 (0.97-52.03)
SRSF5	2	1	34.73 (3.11-388.19)	9.33 (2.26-38.59)
ST6GALNAC3	1	2	8.53 (0.76-95.34)	10.76 (1.46-79.15)
ST8SIA2	1	0	Inf	14.43 (1.95-107.01)
STAM2	2	0	Inf	14.45 (3.47-60.2)
STIP1	1	0	Inf	11.32 (1.54-83.45)
STRADA	1	1	17.09 (1.06-276.44)	5.84 (0.8-42.55)
STX18	1	0	Inf	9.39 (1.28-69.01)
SUDS3	1	2	8.52 (0.76-95.25)	11.04 (1.49-81.82)
SULT2A1	1	3	5.69 (0.58-55.45)	6.33 (0.87-46.18)
SUMO4	1	2	8.54 (0.76-95.43)	32.42 (4.24-248.11)
SYCN	1	1	11.28 (0.7-182.5)	6.61 (0.9-48.35)
SYNGAP1	2	6	5.75 (1.14-29.05)	12.65 (3.04-52.57)
SYT4	1	3	5.67 (0.58-55.25)	6.18 (0.85-45.06)
TADA2B	1	1	17.11 (1.06-276.7)	24.15 (3.19-182.66)
TAF2	1	4	4.26 (0.47-38.69)	5.39 (0.74-39.24)
TAGLN3	1	0	Inf	47.22 (6.01-371.15)
TAS2R39	1	1	17.08 (1.06-276.19)	8.67 (1.18-63.51)
TBC1D10A	2	3	11.54 (1.89-70.3)	5.67 (1.38-23.35)
TBX1	1	0	Inf	8.52 (1.16-62.56)
TBX20	1	4	4.26 (0.47-38.69)	6.26 (0.86-45.71)
TCTN3	1	4	4.24 (0.47-38.47)	5.62 (0.77-40.98)
TDP2	1	0	Inf	13.99 (1.89-103.51)
TM6SF1	2	4	8.65 (1.55-48.13)	10.09 (2.44-41.8)
TMBIM1	1	2	8.54 (0.76-95.4)	5.22 (0.72-38.07)
TMED10	1	2	8.51 (0.76-95.12)	47.24 (6.01-371.33)
TMEM121	1	0	Inf	11.02 (1.48-82.11)
TMEM140	1	4	4.24 (0.47-38.46)	12.65 (1.71-93.4)
TMEM256	1	0	Inf	15.76 (2.11-117.72)
TMEM41A	1	1	17.09 (1.06-276.44)	6.48 (0.89-47.48)
TMEM86A	2	2	17.26 (2.39-124.57)	18.54 (4.43-77.66)
TMEM92	1	0	Inf	21.32 (2.84-159.98)
TMIGD1	1	1	17.09 (1.06-276.44)	4.81 (0.66-34.97)
TMOD2	1	2	8.53 (0.76-95.34)	4.28 (0.59-31.11)
TNFRSF11B	1	1	17.09 (1.06-276.44)	19.26 (2.58-143.89)
TNFRSF25	1	1	15.27 (0.94-247.02)	9.3 (1.26-68.6)
TNFSF15	1	0	Inf	15.24 (2.05-113.14)
TNIP2	1	1	16.88 (1.04-272.91)	8.04 (1.1-58.9)
TOP2A	2	5	6.92 (1.32-36.36)	8.18 (1.98-33.83)
TPH2	1	2	8.54 (0.76-95.4)	5.77 (0.79-42.04)
TRA2A	1	1	17.09 (1.06-276.44)	10.15 (1.38-74.56)
TRIB1	1	1	17.09 (1.06-276.44)	6.62 (0.91-48.33)
TRIM25	1	4	4.18 (0.46-37.94)	8.65 (1.18-63.54)
TRIM32	1	4	4.26 (0.47-38.67)	9.1 (1.24-66.73)
TRIM44	1	0	Inf	12.64 (1.71-93.38)

TRIM52	1	0	Inf	6.47 (0.89-47.21)
TRMT12	1	1	17.1 (1.06-276.53)	7.1 (0.97-51.89)
TRPC3	1	0	Inf	6.96 (0.95-50.83)
TSPO	1	1	16.4 (1.01-265.21)	7.28 (0.99-53.77)
TTC23	1	2	8.53 (0.76-95.34)	5.32 (0.73-38.73)
TTYH3	3	9	5.23 (1.38-19.81)	7.75 (2.41-24.95)
TUBG2	1	0	Inf	4.37 (0.6-31.77)
TXN2	1	3	5.56 (0.57-54.2)	16.71 (2.25-124.29)
UBASH3B	1	0	Inf	8.29 (1.13-60.7)
UBP1	1	2	8.54 (0.76-95.43)	5.5 (0.76-40.09)
UBQLN1	1	1	17.09 (1.06-276.44)	9.14 (1.25-67.11)
UFC1	1	0	Inf	7.63 (1.04-55.82)
USE1	1	0	Inf	14.63 (1.96-108.89)
UTP11L	2	5	6.91 (1.32-36.33)	7.63 (1.85-31.51)
VAX1	1	1	16.38 (1.01-264.83)	23.83 (3.14-180.72)
VEGFC	1	1	17.09 (1.06-276.44)	9.01 (1.23-66.11)
VIMP	1	1	17.08 (1.06-276.19)	6.06 (0.83-44.37)
VPS54	1	4	4.26 (0.47-38.66)	6.82 (0.93-49.79)
VWC2	1	1	9.44 (0.58-152.7)	12.12 (1.59-92.43)
WBP11	1	2	8.53 (0.76-95.28)	8.61 (1.17-63.09)
WDR88	1	1	17.09 (1.06-276.44)	6.41 (0.88-46.78)
WNT4	1	1	16.63 (1.03-268.99)	7.19 (0.98-52.53)
XRCC2	1	0	Inf	9.06 (1.24-66.45)
YBEY	1	0	Inf	16.59 (2.23-123.61)
ZBTB5	1	1	17.09 (1.06-276.36)	9.08 (1.24-66.57)
ZC2HC1A	1	1	17.09 (1.06-276.44)	8.39 (1.14-61.45)
ZCCHC3	1	2	7.11 (0.64-79.42)	16.69 (2.24-124.56)
ZDHHC14	1	0	Inf	7.25 (0.99-53.16)
ZIC1	1	2	6.9 (0.62-77.15)	5.82 (0.8-42.46)
ZNF213	1	2	8.13 (0.73-90.89)	5.59 (0.77-40.74)
ZNF326	1	1	17.05 (1.05-275.81)	7.16 (0.98-52.36)
ZNF34	1	2	8.54 (0.76-95.43)	8.15 (1.11-59.67)
ZNF362	1	1	10.96 (0.68-177.39)	5.3 (0.73-38.64)
ZNF511	1	1	16.23 (1-262.55)	6.12 (0.84-44.7)
ZNF547	1	3	5.69 (0.58-55.45)	7.64 (1.04-55.86)
ZNF624	1	1	17.09 (1.06-276.44)	6.24 (0.86-45.54)
ZNF641	1	2	8.54 (0.76-95.4)	5.16 (0.71-37.54)
