# Supplementary material for: Whole exome sequencing implicates eye development, the unfolded protein response and plasma membrane homeostasis in primary open-angle glaucoma
Source: PLoS One. 2017 Mar 6;12(3):e0172427. doi: 10.1371/journal.pone.0172427 (PMC5338784; doi:10.1371/journal.pone.0172427)
Supplement: S6 Table — (PDF) [file pone.0172427.s008.pdf]

S6 Table: List of enriched genes for normal-tension glaucoma cohort under a loss of function model

Headings:
Gene: HGNC gene name
NTG: Number of cases in normal-tension glaucoma cohort
CTRL: Number of cases in local and AOGC controls
NTG CTRL OR (95% CI): Odds ratio of normal-tension glaucoma cohort compared to controls
NTG NFE OR (95% CI): Odds ratio of normal-tension glaucoma cohort compared to non-Finnish European ExAC public domain data

Gene	NTG	CTRL	NTG CTRL OR (95% CI)	NTG NFE OR (95% CI)
AARS	1	0	Inf	42.04 (5.39-328.12)
AASDH	1	1	17.09 (1.06-276.44)	12.25 (1.66-90.38)
ABCC6	1	0	Inf	9.54 (1.3-70.12)
ABLIM1	1	1	17.07 (1.06-276.07)	53.98 (6.74-432.28)
ACTL7A	1	2	8.54 (0.76-95.43)	47.18 (6-370.82)
ADAM17	1	0	Inf	53.5 (6.68-428.5)
ADD1	1	0	Inf	19.67 (2.62-147.63)
AGL	1	2	8.53 (0.76-95.32)	7.28 (1-53.17)
AKR1A1	1	0	Inf	10.4 (1.41-76.41)
ALKBH1	1	1	17.1 (1.06-276.57)	27.39 (3.61-207.7)
AMBP	1	0	Inf	32.19 (4.21-246.36)
AMFR	1	0	Inf	101.99 (11.75-885.28)
ANXA9	1	1	17.08 (1.06-276.19)	17.04 (2.29-126.82)
APIP	1	0	Inf	36.09 (4.68-278.54)
APOBEC1	1	0	Inf	169.46 (17.39-1650.88)
APOH	1	0	Inf	51.54 (6.5-408.56)
ARHGEF25	1	0	Inf	27.37 (3.6-208.07)
ARHGEF26	1	0	Inf	14.48 (1.95-107.69)
ARID1B	1	0	Inf	61.33 (7.56-497.47)
ARMC2	1	0	Inf	12.75 (1.72-94.26)
ATAD5	1	1	17.09 (1.06-276.44)	57.24 (7.15-458.39)
ATG7	1	0	Inf	22.48 (2.99-168.99)
ATP2C2	1	1	17.09 (1.06-276.44)	4.97 (0.68-36.16)
ATP5G2	1	0	Inf	73.88 (8.96-609.17)
ATP6V0A2	1	0	Inf	34.18 (4.45-262.6)
ATP8B4	1	1	17.09 (1.06-276.32)	7.3 (1-53.36)
ATP9A	1	0	Inf	101.88 (11.74-884.37)
ATP9B	1	2	8.54 (0.76-95.43)	19.74 (2.64-147.67)
ATXN2L	1	0	Inf	21.92 (2.91-165.11)
BCAT2	1	0	Inf	44.46 (5.66-349.49)
BEST3	1	2	8.54 (0.76-95.43)	6.35 (0.87-46.35)
BRF2	1	0	Inf	19.88 (2.66-148.73)
C11orf1	1	0	Inf	15.88 (2.13-118.07)
C12orf40	1	1	17.09 (1.06-276.44)	25.85 (3.41-196)
C17orf67	1	0	Inf	57.56 (7.19-460.96)
C3orf30	1	1	17.09 (1.06-276.32)	30.41 (3.99-231.97)
CAPRIN2	1	0	Inf	25.9 (3.42-195.92)
CARD6	1	3	5.65 (0.58-55.13)	5.31 (0.73-38.7)
CASP14	1	0	Inf	23.58 (3.13-177.56)
CASR	1	2	8.54 (0.76-95.38)	173.54 (17.81-1690.7)
CC2D2A	2	3	11.56 (1.9-70.41)	12.94 (3.1-54.01)
CCDC57	1	1	17 (1.05-274.93)	13.03 (1.76-96.54)
CCDC84	1	1	17.09 (1.06-276.44)	11.27 (1.53-83.12)
CCL18	1	0	Inf	104.11 (11.99-903.66)
CCR5	1	3	5.69 (0.58-55.45)	6.66 (0.91-48.61)
CD200R1L	1	0	Inf	63.71 (7.85-516.77)
CD300LF	1	0	Inf	42.79 (5.48-333.95)
CDAN1	1	2	8.34 (0.75-93.16)	20.47 (2.72-154.17)
CDCP1	1	0	Inf	101.23 (11.66-878.72)

CDON	1	1	17.09 (1.06-276.44)	13.24 (1.79-97.92)
CEP128	1	1	17.09 (1.06-276.44)	17.42 (2.33-129.96)
CEP72	1	0	Inf	14.68 (1.98-108.84)
CH25H	1	1	17.1 (1.06-276.57)	78.97 (9.37-665.32)
CLCN1	1	1	17.09 (1.06-276.44)	11.27 (1.53-83)
CLEC4C	1	0	Inf	6.48 (0.89-47.25)
CLEC4M	1	0	Inf	73.98 (8.97-609.96)
CLHC1	1	2	8.53 (0.76-95.31)	11.46 (1.56-84.41)
COG2	1	0	Inf	81.43 (9.66-686.01)
COL18A1	2	3	11.12 (1.82-67.75)	11.43 (2.74-47.66)
CORO7	1	1	15.98 (0.99-258.51)	12.52 (1.68-93)
CPD	1	0	Inf	57.84 (7.22-463.24)
CYP2E1	1	0	Inf	28.49 (3.75-216.63)
CYP2J2	1	1	16.84 (1.04-272.28)	14.41 (1.95-106.67)
DCLRE1C	2	1	34.74 (3.11-388.28)	22.22 (5.28-93.47)
DDX51	2	0	Inf	11.86 (2.86-49.25)
DGKZ	1	0	Inf	76.62 (8.45-694.98)
DHX32	1	1	17.09 (1.06-276.44)	24.31 (3.22-183.46)
DKK4	1	0	Inf	64.44 (7.94-522.7)
DMPK	1	1	15.76 (0.97-254.85)	8.42 (1.14-62.08)
DMRT3	1	1	17.09 (1.06-276.44)	43.32 (5.55-338.13)
DNAJC16	1	0	Inf	15.7 (2.12-116.55)
DNMT3A	1	3	5.69 (0.58-55.47)	5.8 (0.8-42.24)
DOPEY2	1	1	17.09 (1.06-276.36)	7.06 (0.97-51.6)
DUSP4	1	0	Inf	117.33 (12.94-1064.23)
ECH1	1	1	17.09 (1.06-276.32)	22.68 (2.99-172)
EGFL8	1	0	Inf	62.79 (7.74-509.31)
EHHADH	1	3	5.68 (0.58-55.37)	10.73 (1.46-78.95)
ELP2	1	2	8.54 (0.76-95.4)	18.54 (2.49-138.36)
FAM111A	1	2	8.54 (0.76-95.43)	17.78 (2.39-132.52)
FAM118B	1	0	Inf	86.53 (10.27-729.05)
FAM120B	1	0	Inf	12.5 (1.69-92.44)
FAM124A	1	1	17.08 (1.06-276.19)	54.16 (6.76-433.75)
FAM178A	1	0	Inf	31.21 (4.08-238.89)
FAM186A	1	1	17.09 (1.06-276.44)	5.32 (0.68-41.54)
FAM32A	1	0	Inf	384.41 (23.78-6212.96)
FAM69C	1	1	16.58 (1.03-268.11)	104.09 (11.99-903.54)
FAM71E2	1	0	Inf	Inf
FAM84B	1	0	Inf	110.12 (12.14-998.81)
FAR2	1	0	Inf	259.93 (23.28-2902.75)
FARSA	1	0	Inf	82.56 (9.8-695.53)
FASTKD2	1	0	Inf	36.54 (4.73-281.98)
FASTKD3	1	2	8.54 (0.76-95.43)	11.21 (1.52-82.56)
FBXO24	1	0	Inf	35.49 (4.58-275.37)
FCN1	1	1	17.06 (1.06-275.94)	7.48 (1.02-54.67)
FLOT2	1	0	Inf	60.88 (7.51-493.83)
FMO5	1	0	Inf	15.21 (2.05-112.78)
GABRR2	1	3	5.69 (0.58-55.45)	20.58 (2.74-154.44)
GAL3ST3	1	0	Inf	52.74 (6.4-434.88)
GALNS	1	0	Inf	41.08 (5.23-322.89)

GALNT14	1	2	8.54 (0.76-95.43)	21.6 (2.87-162.35)
GBA2	1	0	Inf	31.91 (4.17-244.19)
GCNT2	1	0	Inf	19.26 (2.58-143.89)
GIT2	1	0	Inf	124.31 (13.7-1127.54)
GJA8	1	0	Inf	56.47 (7.05-452.22)
GLE1	1	1	17.09 (1.06-276.44)	30.27 (3.97-230.84)
GLMN	1	2	8.49 (0.76-94.87)	22.98 (3.05-173.03)
GML	1	0	Inf	51.93 (6.55-411.65)
GNL3	1	0	Inf	12.57 (1.7-92.75)
GPBAR1	1	0	Inf	22.06 (2.92-166.44)
GPX5	1	0	Inf	130.13 (14.35-1180.3)
H6PD	1	2	8.53 (0.76-95.34)	18.59 (2.49-138.89)
HCN1	1	0	Inf	101.79 (11.73-883.51)
HEPHL1	2	5	6.92 (1.32-36.35)	14.96 (3.58-62.41)
HIST1H4A	1	0	Inf	22.39 (2.98-168.3)
HSPA4L	1	1	17.09 (1.06-276.32)	45.7 (5.81-359.21)
HYAL4	1	2	8.54 (0.76-95.43)	11.26 (1.53-82.87)
IFI44L	1	0	Inf	31.57 (4.12-241.6)
IGFBP6	1	0	Inf	32.47 (4.24-248.54)
IGFN1	2	4	8.65 (1.56-48.14)	13.37 (3.18-56.21)
IKBKAP	1	2	8.54 (0.76-95.47)	21.52 (2.87-161.47)
IQGAP2	1	3	5.69 (0.58-55.43)	6.74 (0.92-49.18)
KAT8	1	0	Inf	124.96 (13.78-1133.4)
KCNH5	1	1	17.09 (1.06-276.32)	16.01 (2.15-118.91)
KCNJ14	1	1	16.82 (1.04-272.02)	76.93 (9.13-648.14)
KCNU1	1	0	Inf	12.83 (1.74-94.89)
KHDC1L	1	0	Inf	20.57 (2.65-159.6)
KHNYN	1	1	16.87 (1.04-272.78)	6.13 (0.84-44.72)
KIAA1549	1	1	17.09 (1.06-276.44)	120.82 (13.32-1095.88)
KIAA1614	1	0	Inf	6.89 (0.94-50.36)
KIF12	1	0	Inf	38.24 (4.93-296.63)
KLHL38	1	3	5.69 (0.58-55.47)	12.05 (1.63-88.83)
KNG1	1	0	Inf	32.39 (4.23-247.9)
KRT75	1	2	8.54 (0.76-95.38)	7.99 (1.09-58.47)
KRT76	1	0	Inf	31.61 (4.13-241.89)
LAMTOR3	1	0	Inf	501.63 (31.04-8107.58)
LCE5A	1	0	Inf	64.35 (7.93-522.03)
LHX4	1	0	Inf	130.13 (14.35-1180.35)
LIMS2	1	2	8.47 (0.76-94.64)	17.22 (2.3-129.24)
LMBR1	1	1	17.09 (1.06-276.44)	8.94 (1.22-65.64)
LMBRD2	1	0	Inf	45.81 (5.83-360.09)
LPIN3	2	3	11.56 (1.9-70.41)	11.46 (2.76-47.57)
LRRC15	1	3	5.69 (0.58-55.45)	50.98 (6.43-404.13)
LRRC23	1	0	Inf	19.86 (2.65-148.58)
LRRC34	2	2	17.27 (2.39-124.63)	14.44 (3.47-60.13)
LRRC66	1	0	Inf	7.82 (1.07-57.21)
LRRC74A	1	0	Inf	12.67 (1.71-93.84)
LRRCC1	1	0	Inf	13.83 (1.87-102.52)
LRSAM1	1	0	Inf	29.77 (3.9-227.05)
MAP4K3	1	0	Inf	33.6 (4.37-258.14)

MAPRE1	1	0	Inf	521.34 (32.26-8426.15)
MB21D1	1	0	Inf	23.45 (3.08-178.88)
MCTP2	1	3	5.68 (0.58-55.38)	9.48 (1.29-69.57)
MIB1	1	3	5.69 (0.58-55.47)	5.79 (0.79-42.19)
MICALCL	1	1	17.09 (1.06-276.44)	5.05 (0.69-36.74)
MICU1	1	0	Inf	19.42 (2.58-146.24)
MICU2	1	0	Inf	36.13 (4.66-280.31)
MNX1	1	0	Inf	Inf
MRPS7	1	0	Inf	127.46 (14.05-1156.13)
MRRF	1	0	Inf	37.14 (4.81-286.61)
MTERF4	1	0	Inf	19.22 (2.57-143.61)
MTHFS	1	0	Inf	74.47 (9.03-614.01)
MTHFSD	1	1	17.09 (1.06-276.44)	14.75 (1.98-109.68)
MTNR1A	1	2	8.54 (0.76-95.43)	86.85 (10.31-731.74)
MUTYH	1	2	8.47 (0.76-94.64)	33.83 (4.4-259.9)
MYH10	1	1	17.09 (1.06-276.44)	30.35 (3.97-232.32)
MYLK2	1	0	Inf	Inf
NDE1	1	0	Inf	74.15 (8.99-611.37)
NOB1	1	0	Inf	50.27 (6.34-398.5)
NPHS2	1	0	Inf	36.82 (4.77-284.21)
NPTX2	1	0	Inf	499.27 (30.89-8069.32)
NSUN7	1	0	Inf	29.19 (3.76-226.47)
NUP214	1	0	Inf	15.66 (2.11-116.33)
OR10AD1	1	0	Inf	39.92 (5.15-309.67)
OR10G7	1	0	Inf	65.09 (8.02-528.01)
OR6T1	1	0	Inf	47.37 (6.03-372.33)
ORC6	1	0	Inf	40.55 (5.2-316.49)
OTOP3	1	0	Inf	63.05 (7.77-511.42)
OVGP1	2	0	Inf	24.71 (5.85-104.37)
OVOL1	1	0	Inf	364.2 (22.53-5886.31)
P2RX1	1	0	Inf	40.32 (5.17-314.67)
PALB2	1	1	17.09 (1.06-276.44)	12.02 (1.63-88.63)
PAPSS1	1	0	Inf	5.93 (0.81-43.24)
PARK7	1	0	Inf	14.65 (1.98-108.59)
PCDHGA5	1	1	17.09 (1.06-276.44)	7.11 (0.97-51.93)
PCDHGA8	1	0	Inf	30.34 (3.98-231.42)
PCSK7	1	0	Inf	54.04 (6.75-432.83)
PCSK9	1	0	Inf	18.36 (2.45-137.77)
PDE3B	1	2	8.18 (0.73-91.37)	6.53 (0.89-47.67)
PDE6H	1	0	Inf	72.16 (8.75-594.97)
PLA2G4E	1	2	8.53 (0.76-95.34)	16.03 (2.14-120.28)
PLCB4	1	0	Inf	63.45 (7.82-514.67)
PLCD1	1	3	5.68 (0.58-55.42)	12.11 (1.64-89.33)
PLD1	1	0	Inf	9.55 (1.3-70.11)
PLOD1	1	0	Inf	26.66 (3.52-202.12)
PODN	1	0	Inf	21.25 (2.82-160.38)
PROCA1	1	1	17.09 (1.06-276.44)	20.42 (2.73-152.95)
PRR23A	1	3	5.61 (0.58-54.69)	330.28 (20.43-5338.1)
PRSS33	1	2	8.18 (0.73-91.41)	29.57 (3.76-232.4)
PRSS36	1	0	Inf	7.04 (0.96-51.47)

PSAT1	1	0	Inf	47.33 (6.02-372.01)
PTDSS2	1	0	Inf	73.8 (8.95-608.5)
PTK2	1	0	Inf	259.93 (23.28-2902.68)
PUSL1	2	0	Inf	20.27 (4.76-86.29)
QRICH2	1	2	8.46 (0.76-94.5)	5.88 (0.81-42.85)
RAD51AP2	1	0	Inf	10.13 (1.38-74.58)
RCN3	1	1	16.83 (1.04-272.15)	29.82 (3.88-229.11)
RGL3	1	1	14.99 (0.93-242.48)	15.15 (2.03-112.76)
RMDN3	1	2	8.53 (0.76-95.34)	55.47 (6.93-444.23)
RNF112	1	0	Inf	147.51 (15.14-1437.05)
RSBN1	1	0	Inf	8.46 (1.15-62.03)
RTN4IP1	2	0	Inf	26.19 (6.2-110.7)
SAMD11	1	0	Inf	6.74 (0.92-49.38)
SAXO2	1	0	Inf	12.28 (1.66-90.66)
SCN7A	1	1	17.07 (1.06-276.02)	12.31 (1.66-91.34)
SEC22C	1	0	Inf	23.25 (3.09-175.09)
SETX	1	1	17.09 (1.06-276.44)	22.21 (2.95-166.95)
SH3TC2	1	1	17.09 (1.06-276.32)	18.58 (2.49-138.63)
SHPK	1	2	8.54 (0.76-95.38)	27.21 (3.58-206.91)
SIAH3	1	0	Inf	258.43 (23.14-2885.99)
SLC12A9	1	1	16.76 (1.04-271.01)	22.76 (3.02-171.78)
SLC15A3	1	0	Inf	15.73 (2.12-116.87)
SLC25A24	1	1	17.08 (1.06-276.19)	10.35 (1.41-76.13)
SLC26A8	1	1	17.09 (1.06-276.44)	6.53 (0.89-47.71)
SLC2A7	1	2	8.53 (0.76-95.34)	14.35 (1.94-106.45)
SLC47A1	1	0	Inf	25.76 (3.41-194.82)
SLC5A9	1	2	8.53 (0.76-95.29)	9.58 (1.3-70.28)
SMIM11	1	0	Inf	253.87 (22.73-2835.07)
SNX1	1	1	17.08 (1.06-276.19)	127.6 (14.07-1157.38)
STAM2	2	0	Inf	80.89 (17.89-365.85)
STKLD1	1	1	17.08 (1.06-276.19)	9.71 (1.32-71.46)
STRADA	1	1	17.09 (1.06-276.44)	36.24 (4.7-279.68)
STRN3	1	1	17.08 (1.06-276.19)	64.49 (7.95-523.1)
SULT1C3	1	1	17.11 (1.06-276.7)	19.85 (2.65-148.46)
SULT2A1	1	0	Inf	39.94 (5.15-309.87)
SYTL3	1	2	8.54 (0.76-95.43)	9.07 (1.24-66.6)
TACC3	1	0	Inf	36.6 (4.74-282.48)
TAS2R10	1	0	Inf	16.65 (2.24-123.81)
TCF12	1	0	Inf	104 (11.98-902.71)
TFR2	2	0	Inf	77.3 (16.95-352.48)
TGS1	1	0	Inf	25.49 (3.36-193.29)
TICRR	1	1	17.08 (1.06-276.19)	12.26 (1.66-90.53)
TLR10	1	2	8.54 (0.76-95.43)	9.53 (1.3-69.95)
TM4SF1	1	1	17.09 (1.06-276.44)	64.99 (8.01-527.19)
TMBIM1	1	1	17.09 (1.06-276.32)	28.28 (3.69-216.41)
TMEM256	1	0	Inf	57.69 (7.2-462.03)
TMEM86A	1	0	Inf	64.46 (7.95-522.87)
TMPRSS9	1	1	15.93 (0.98-257.63)	10.5 (1.42-77.47)
TNFRSF18	1	0	Inf	14.24 (1.91-106.41)
TNRC6C	1	0	Inf	79.7 (9.18-691.8)

TRDMT1	1	0	Inf	48.96 (6.18-388.1)
TRIM52	1	0	Inf	57.24 (7.15-458.4)
TRPC3	1	0	Inf	34.46 (4.46-265.94)
TRPM8	1	0	Inf	22.3 (2.97-167.64)
TRPV1	1	1	17.09 (1.06-276.44)	9.99 (1.36-73.63)
TSSK4	1	0	Inf	17.3 (2.32-128.75)
TXN2	1	0	Inf	74.11 (8.99-611.02)
TXNDC12	1	0	Inf	159.85 (16.41-1557.26)
UFC1	1	0	Inf	130.29 (14.36-1181.77)
UGDH	1	0	Inf	19.67 (2.63-147.15)
UGGT2	1	0	Inf	13.34 (1.8-98.67)
UPP2	1	0	Inf	17.8 (2.39-132.64)
USE1	1	0	Inf	123.81 (13.65-1123.01)
USP44	2	0	Inf	38.9 (9.06-167.09)
VARS2	1	0	Inf	6.59 (0.9-48.16)
VCL	1	1	17.1 (1.06-276.57)	46.86 (5.96-368.35)
VIPR1	1	2	8.53 (0.76-95.31)	39.74 (5.09-310.17)
VPREB1	1	0	Inf	85.29 (10.12-718.55)
VPS9D1	1	0	Inf	52.88 (6.52-428.93)
VWF	1	1	17.05 (1.05-275.69)	7.14 (0.98-52.21)
WBSCR27	1	0	Inf	33.77 (4.38-260.63)
WDR38	2	3	11.45 (1.88-69.77)	28.4 (6.7-120.38)
ZCCHC10	1	0	Inf	169.48 (17.4-1651.12)
ZCCHC7	1	0	Inf	47.14 (6-370.5)
ZMYM6	1	0	Inf	20.89 (2.77-157.67)
ZNF256	1	0	Inf	14.43 (1.95-106.84)
ZNF337	1	3	5.69 (0.58-55.45)	8 (1.09-58.56)
ZNF500	1	0	Inf	62.59 (7.72-507.69)
ZNF835	1	1	17.07 (1.06-276.07)	10.55 (1.43-77.66)
